# Live imaging of avian epiblast and anterior mesendoderm grafting reveals the complexity of cell dynamics during early brain development

**DOI:** 10.1242/dev.199999

**Published:** 2022-03-28

**Authors:** Koya Yoshihi, Kagayaki Kato, Hideaki Iida, Machiko Teramoto, Akihito Kawamura, Yusaku Watanabe, Mitsuo Nunome, Mikiharu Nakano, Yoichi Matsuda, Yuki Sato, Hidenobu Mizuno, Takuji Iwasato, Yasuo Ishii, Hisato Kondoh

**Affiliations:** 1 Faculty of Life Sciences, Kyoto Sangyo University, Motoyama, Kamigamo, Kita-ku, Kyoto 603-8555, Japan; 2 National Institutes of Natural Sciences, Exploratory Research Center on Life and Living Systems (ExCELLS), National Institute for Basic Biology, Okazaki, Aichi 444-8787, Japan; 3 Institute for Protein Dynamics, Kyoto Sangyo University, Motoyama, Kamigamo, Kita-ku, Kyoto 603-8555, Japan; 4 Avian Bioscience Research Center, Graduate School of Bioagricultural Sciences, Nagoya University, Furo-cho, Chikusa-ku, Nagoya 464-8601, Japan; 5 Department of Anatomy and Cell Biology, Graduate School of Medical Sciences, Kyushu University, 3-1-1 Maidashi, Higashi-ku, Fukuoka 812-8582, Japan; 6 Laboratory of Mammalian Neural Circuits, National Institute of Genetics (NIG), Mishima, Shizuoka 411-8540, Japan; 7 International Research Center for Medical Sciences (IRCMS), Kumamoto University, 2-2-1 Honjo, Chuo-ku, Kumamoto City 860-0811, Japan; 8 Department of Biology, School of Medicine, Tokyo Women's Medical University, Shinjuku-ku, Tokyo 162-8666, Japan; 9 Institute for Comprehensive Research, Kyoto Sangyo University, Motoyama, Kamigamo, Kita-ku, Kyoto 603-8555, Japan; 10 JT Biohistory Research Hall, 1-1 Murasaki-cho, Takatsuki, Osaka 569-1125, Japan

**Keywords:** Epiblast, Node, Anterior mesendoderm, Brain primordia, Live imaging, Chicken

## Abstract

Despite previous intensive investigations on epiblast cell migration in avian embryos during primitive streak development before stage (st.) 4, this migration at later stages of brain development has remained uninvestigated. By live imaging of epiblast cells sparsely labeled with green fluorescence protein, we investigated anterior epiblast cell migration to form individual brain portions. Anterior epiblast cells from a broad area migrated collectively towards the head axis during st. 5-7 at a rate of 70-110 µm/h, changing directions from diagonal to parallel and forming the brain portions and abutting head ectoderm. This analysis revised the previously published head portion precursor map in anterior epiblasts at st. 4/5. Grafting outside the brain precursor region of mCherry-expressing nodes producing anterior mesendoderm (AME) or isolated AME tissues elicited new cell migration towards ectopic AME tissues. These locally convergent cells developed into secondary brains with portions that depended on the ectopic AME position in the anterior epiblast. Thus, anterior epiblast cells are bipotent for brain/head ectoderm development with given brain portion specificities. A brain portion potential map is proposed, also accounting for previous observations.

## Abstract (precis)

**Summary:** The first high-resolution live imaging of anterior epiblast cells at the brain-forming stages in avian embryos is reported, revealing their long-distance migration and interaction with the anterior mesendoderm to form brain tissues.

## INTRODUCTION

Avian embryos around the gastrulation stages are flat, relatively large (several mm^2^), and amenable to live observation and manipulation, particularly owing to the development of *ex ovo* culture techniques pioneered by Waddington (1932) with subsequent refinements (New, 1955; Chapman et al., 2001; Uchikawa et al., 2003).

These features have been utilized to analyze the dynamic migrations of posterior epiblast cells during primitive streak formation before stage (st.) 4 (Rozbicki et al., 2015; Chuai et al., 2012; Voiculescu, 2020). The node (Hensen's node) is formed at the anterior streak end around the beginning of st. 4; then, at st. 5, a part of the node develops into the anteriorly extending process, referred to as the anterior mesendoderm (AME) in this study. However, there has been little investigation of epiblast cell migration in the brain-forming stages, presumably because anterior epiblast movements are minimal at earlier stages (Rozbicki et al., 2015).

In this study, a technique to sparsely label anterior epiblast cells with enhanced green fluorescent protein (EGFP) was established and dubbed Supernova (SN). Live SN-EGFP-labeled epiblast cell migration was recorded, followed by time-resolved and extended time-span analyses of epiblast cell migration. The analysis showed that the precursors of the brain and head ectoderm tissues in the broad anterior epiblast migrate long distances towards the midline before they form head tissues. This analysis provides a new brain portion precursor map, revising the map published by Fernández-Garre et al. (2002) (Fig. S1A).

Live imaging was extended to the analysis of the impact of grafting st. 4 nodes at various positions outside the brain precursor region but within the area pellucida, labeling the nodes with mCherry and the epiblast with SN-EGFP. Only when the node grafts were in the anterior half of the embryo did the nodes develop into the AME tissue, which further developed into the prechordal plate (PP) and anterior notochord (ANC). The st. 4 node-derived AME or directly grafted st. 5 AME, collectively called graft-derived AME (gAME), altered the migration pattern of the epiblast cells, making them converge on the ectopic gAME in addition to the midline underlain by the host AME (hAME). The convergent epiblast cells outside the brain precursor region developed into secondary brain tissues. Further analysis indicated that all anterior epiblast cells are bipotent for brain or head ectoderm development and bear brain portion specificities.

Integrating these new observations with those of previous studies with grafting nodes at the periphery of or outside the area pellucida (e.g. Dias and Schoenwolf, 1990; Knoetgen et al., 2000; Storey et al., 1992; Streit et al., 1997), a brain portion potential map is presented, which is supported by additional AME grafting data.

## RESULTS

### Random fluorescent labeling of epiblast cells using the SN technique reveals cell convergence from a broad area towards the embryo axis to form the brain primordia

**Fig. 1.**
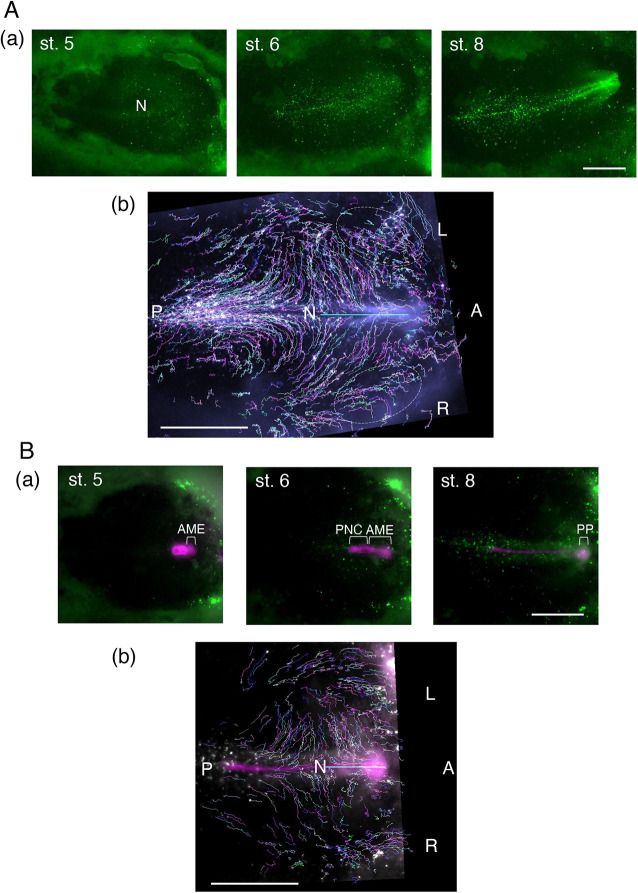
**Long-range convergence of SN-EGFP-labeled epiblast cells towards the midline.** (Aa) Snapshots of an SN-EGFP-labeled chicken embryo at different developmental stages; taken from Movie 1. (Ab) Trajectories with random colors of SN-EGFP-labeled cells during st. 5-8 of the same embryo; taken from Movie 2. Dashed ovals indicate the trajectories showing cell migration across the area pellucida/opaca boundary. (Ba) Snapshots of a chicken embryo with SN-EGFP labeling receiving the homotopic mCherry-labeled quail node; excerpts from Movie 3. Positions of AME, PNC and PP are indicated. (Bb) Trajectories of SN-EGFP-labeled cells during st. 5-8 of the same embryo; taken from Movie 4. N indicates the node position; cyan bar, the head axis. A, anterior; L, left; P, posterior; R, right. Scale bars: 1 mm.

The SN technique, originally developed to label cells randomly with fluorescent proteins in developing mouse brains (Luo et al., 2016; Mizuno et al., 2014), was adopted to randomly label chicken embryo epiblast cells more quickly (Fig. S1B). Two representative time-lapse recordings of SN-EGFP-labeled chicken embryos are shown, one with SN-EGFP labeling only (*n*=11) and the other with a homotopic node graft from an mCherry-transgenic Japanese quail (Huss et al., 2015) (*n*=2) (Fig. 1A,B, Movies 1 and 3). The labeled cells were widely distributed in the epiblast at the beginning and subsequently converged towards the embryo axis and were incorporated into the forming brain and head ectoderm. Immunostaining of electroporated embryo sections confirmed epiblast-specific labeling by SN-EGFP (Fig. S1C).

The node excised from a st. 4 embryo develops into the AME and posterior notochord (PNC) after grafting (Fig. 1Ba, Fig. S2). The AME further develops into the PP and the ANC (Fig. S2). In contrast, late st. 3 nodes also develop into the endoderm, whereas st. 5 nodes develop into the PNC and somites, without production of the AME (Fig. S2).

Following the electroporation of st. 4 epiblasts with SN vectors, EGFP fluorescence of sparsely labeled epiblast cells became detectable in 3-4 h. This was shortly before or around the time when the embryos reached st. 5, and the node started to extend the AME anteriorly, which later developed into the PP and ANC (Fig. 1Ba, Movie 3). Movie 3 also shows that the PNC extension initiates later.

To analyze the trajectories of these cells, embryo images in all frames were reoriented so that the node position at st. 5 was kept at the posterior end of the horizontal head axis. The trajectories of SN-EGFP-labeled cells in the embryos in Fig. 1Aa and 1Ba over the period of st. 5-8 covering ∼9 h are shown in Fig. 1Ab,Bb and Movies 2 and 4. These trajectories confirmed that the anterior epiblast cells in the wide area converged to the embryo axis underlain by the AME.

**Fig. 2.**
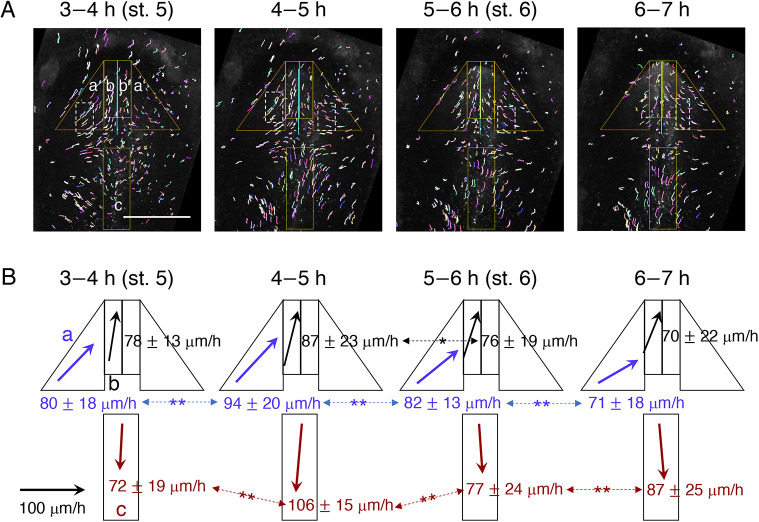
**Time-resolved migration profiles of SN-EGFP-labeled epiblast cells.** (A) One-hour cell migration tracks of an embryo (Fig. S3A) in the intervals shown at the top from the start of st. 5. a,a′, b,b′ and c on the panels indicate areas where cell migration rates and directions were scored. The track areas showing coherent cell migrations are enclosed in dashed boxes. Scale bar: 1 mm. (B) Schematics of the changes in cell migration rates and directions in areas a-c of the epiblast (color-coded by blue, black and red) at each developmental stage, averaged over three SN-EGFP–labeled embryos in Fig. S3. Mean±s.d. of the cell migration rate in each area is indicated. The statistical significance of the migration rate changes was assessed using Mann–Whitney *U*-test and is indicated on the double-headed arrows: *P<0.05; **P<0.01. The horizontal arrow on the left represents the 100 µm/h migration rate.

To characterize epiblast cell migration profiles more precisely during brain-forming stages, short cell trajectories were drawn every 1 h from mid st. 5 to late st. 6 (Fig. 2, Fig. S3). Data from three representative embryos with different label distributions were combined to minimize the heterogeneity in the spatial resolutions in the analysis (Fig. S3). They had dense SN-EGFP labeling close to the anterior axis (Fig. S3A), in the medium lateral positions (Fig. S3B) and further towards the periphery (Fig. S3C).

**Fig. 3.**
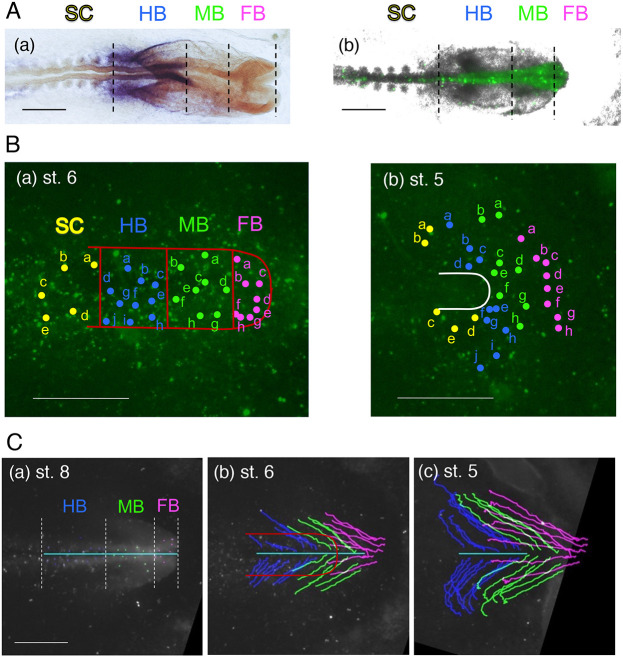
**Tracking the origin of head tissue portions at different axial levels back to st. 5.** (A) Determination of the brain portions in the SN-EGFP-labeled embryo at the endpoint of live imaging. (Aa) An embryo at st. 9 hybridized for *Gbx2* (expressed in HB, purple) and *Otx2* (expressed in FB and MB, orange). The FB/MB boundary was at the posterior end of the FB lateral bulging. (Ab) SN-EGFP image of the same embryo aligned with the image in Aa. The brain portion boundaries were traced back to st. 6 (Ba), using brightly labeled landmark cells as guides (Fig. S4). (B) The precursor distribution for individual brain portions of the same embryo at st. 6 (Ba) and st. 5 (Bb). (Ba) The precursor regions for individual brain portions, FB, MB, HB and SC, at st. 6. The estimated lateral limit of the brain precursors at st. 6 was 200 µm from the head axis, and the anterior limit was immediately posterior to the anterior ectodermal ridge. (Bb) The original positions at st. 5 of the cells in Ba, showing correspondences using alphabetical labeling and color-coding. White U-shaped line indicates the node contour. (C) Long tracks of SN-EGFP-labeled brain-abutting head ectoderm cells, with color-coding of brain portion axial levels using the same embryo. (Ca) The selected SN-EGFP-labeled cells at st. 8. (Cb) An intermediate of backtracking at st. 6. The track lines with their ends within the neural plate region (outlined in red) represent dorsal brain cells. (Cc) Track lines from st. 8 to st. 5. Scale bars: 500 µm.

Three epiblast regions with distinct SN-EGFP-labeled cell migration patterns were noted (Fig. 2, Fig. S3): (a,a′) bilateral right triangular areas where cells converged toward the axial direction at moderate angles; (b,b′) the bilateral axial 200-µm zone where cells migrated anteriorly, coinciding with the st. 6 head precursor zone (Fig. 3B, Fig. S5); and (c) the ∼400 µm-wide region at the post-node level where cells migrated posteriorly along the embryo axis.

The cell migration profiles in these three regions were grossly similar among the embryos (Fig. S3). However, variations existed among embryos and even between embryo sides concerning the time axis-directed cell convergence began (e.g. the cell migration in the embryo left side in Fig. 2A was ∼1 h earlier than the right side; Fig. S3). This variation in the cell dynamics presumably contributed to embryo-dependent variations in the brain portion precursor distributions (Fig. S5). In region a of any given embryo, areas of coherent cell migration (parallel trajectories with similar rates) covering several hundred µm^2^ were observed (Fig. 2A, dashed boxes), indicating that cells migrated collectively.

The statistics of cell migration in the three embryo areas and four timespans are detailed in Fig. S3D. A summary is given in Fig. 2B relating to the embryo trajectories shown in Fig. 2A. Cell migration proceeded at 70-110 µm/h, with moderate changes in the rate and direction. The cell migration rate towards the head axis in area a was the highest at late st. 5 (Fig. 2B, 4-5 h). The anterior cell migration along the head axis in area b slowed down after entering st. 6. Posterior epiblast cell migration at the post-node level in region c has not been characterized previously, but likely contributes to anterior spinal cord development, which will be reported elsewhere.

### Cell-tracking analysis revises the precursor map of brain regions and adjoining head ectoderm at st. 5

Tracking the long cell trajectories from the brain portions and abutting head ectoderm back to earlier stages would allow brain portion precursor maps to be created. Four embryos with high density SN-EGFP labeling were fixed at st. 8/9 immediately after the time-lapse recording and subjected to whole-mount *in situ* hybridization for *Otx2* [expressed in the forebrain (FB) and midbrain (MB), stained orange] and *Gbx2* [expressed in the hindbrain (HB), stained purple] (Fig. 3Aa). The FB/MB boundary was judged by FB lateral bulging. These boundaries were copied onto the SN-EGFP image of the same embryo using somites and other structural features to align the two images (Fig. 3Ab, Fig. S4) and traced back using brightly labeled cells as landmarks to st. 6 (when the neural plate was formed) and early st. 5 (when anterior epiblast cell migration was initiated) (Fig. 3B).

In the st. 6 embryos, the anterior limit of the neural plate was immediately posterior to the anterior ectodermal ridge. SN-EGFP backtracking allowed the FB/MB and MB/HB precursor boundaries to be determined (Fig. 3Ba, Fig. S5). The lateral limit of the neural plate was determined to be 200 µm from the embryo axis after testing the forward tracking of the labeled cells, coinciding with zone b in Fig. 2A where the cells migrated along the head axis. A previous study using isolated grafts of neural plate portions indicated that the anteroposterior (AP) pattern of the neural plate had been established by st. 6-7 (Rowan et al., 1999). The positions of SN-EGFP-labeled precursors for different brain portions in st. 6 neural plates are shown in Fig. 3Ba and Fig. S5.

**Fig. 4.**
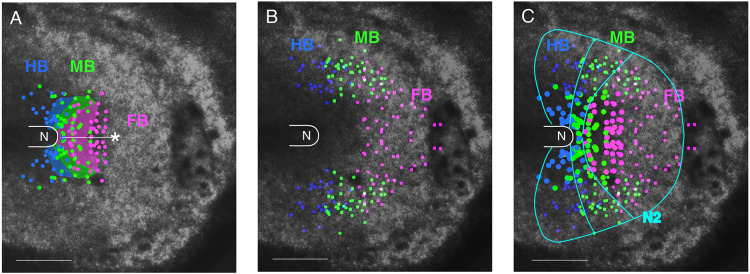
**Comparison of the distribution of head tissue precursors at st. 4/5.** Data for st. 5 embryos are shown. N, the node position. (A) Positions at st. 5 of the precursors for different brain portions, FB, MB and HB, indicated by color-coded dots, compiled from data for the four embryos shown in Fig. S4. The data are compared with the st. 4 brain portion precursor map by Fernández-Garre et al. (2002) shown as shaded areas. As the cell displacements in the anterior epiblast are minimal between st. 4 and st. 5 (Fig. S6, Movie 4), brain precursor distributions at these stages can be superimposed. The asterisk indicates the anterior limit of the FB precursor reported by Schoenwolf and Alvarez (1991). (B) The distribution at st. 5 of the precursors for dorsal brain/head ectoderm at st. 8/9 in three embryos. (C) Combination of the data in A and B with the dots for brain precursor positions enlarged. Rough boundaries of the above precursor regions and outer limit of *Sox2* N2 enhancer activity at st. 5 (Fig. S1A) are drawn in cyan. Scale bars: 500 µm.

These precursors were further traced back to st. 5 (Fig. 3Ba, Fig. S5). The distribution of brain portion precursors at st. 5 in four embryos (Fig. S5) indicated substantial embryo-to-embryo variations; the anterior extent of the precursor distribution from the node varied from 210 to 450 µm, whereas the AP order of the FB/MB/HB precursors st. 6 was more or less conserved. This variation presumably reflects embryo-dependent differences in the timing of lateral and axial cell migration (Fig. S3). Thus, the superimposition of the distribution of brain portion precursors of the four embryos in Fig. 4A reveals the overlapping regions of different brain portion precursors, reflecting the variability in the distribution among embryos (Fig. S4) but not mosaicism within an embryo.

The brain portion precursor map is considered to be similar between st. 4 and early st. 5 because the cell displacements are minimal between these stages (Fig. S6, Movie 4). The st. 5 brain portion precursor map was consistent with the previously published brain precursor map at st. 4 (Fernández-Garre et al., 2002) concerning the gross brain precursor positioning. However, the map in Fig. 4A, based on single-cell analyses, revises the tissue grafting-based map of Fernández-Garre et al. in the following ways (Fig. 4, Fig. S5): (1) the FB precursor anterior limits varied among embryos in the range 210-450 µm from the node tip; (2) these brain precursor anterior limit lines did not show an indentation on the embryo axis, which was drawn by Fernández-Garre et al.; and (3) MB precursors and some HB precursors were always present between the FB precursors and the node, which were missing in the map drawn by Fernández-Garre et al. The possible causes of these discrepancies are discussed below.

The precursor distribution analysis was extended to the brain-proximal head ectoderm. Long-distance tracking of SN-EGFP-labeled dorsal brain and proximal head ectoderm was performed in st. 8-9 embryos back to st. 5, using a protocol described in the Materials and Methods section. Fig. 3C shows an embryo case starting from st. 8 (Fig. 3A,Cc). The tracking lines with the termini in the brain portions at st. 6 (Fig. 3Cb) represent dorsal brain precursors and other head ectoderm precursors. Many trajectories ended >500 µm away from the head axis at st. 5 (Fig. 3Cc). The precursor map of the brain-abutting head ectoderm of the FB, MB and HB axial levels was drawn, representing the data of three embryos (Fig. 4B). The precursor distributions for the individual brain portions (node-proximal) and abutting head ectoderm (node-distal) at the same AP level (distinguished by the dot size) seamlessly overlapped at their margins (Fig. 4C).

### Grafting st. 4 quail nodes outside the brain precursor region reveals that only the anterior epiblast has the potential to develop into secondary brain tissues

The analysis described above indicated that the majority of anterior epiblast cells at st. 4-5 develop into brain or head ectoderm tissues. However, these cell fates do not appear to be fixed at these stages. The brain/head ectoderm precursor boundary was not sharply demarcated (Fig. 4C). Strong *Sox2* expression, suggesting neural developmental potential, extended outside the brain precursor regions at st. 5 (Fig. S1A). Moreover, *Sox2* N2 enhancer activity, covering the entire anterior epiblast, provides cells with *Sox2* activation potential (Iwafuchi-Doi et al., 2011; Fig. 4C, Fig. S1A). Furthermore, the head ectoderm-marking *Dlx5* expressed outside the *Sox2*-expressing domain of the anterior epiblast was downregulated shortly after the grafting of a st. 4 node therein, followed by the thickening of local tissue, indicative of neural plate development (Pera et al., 1999).

Based on the above information, the mechanisms underlying the differentiation of anterior epiblast cells towards brain/head ectoderm fates were investigated by node grafting at various positions of the epiblast. [C. H. Waddington's pioneering studies (Waddington, 1932; Waddington and Schmidt, 1933) showed that the grafting of anterior primitive streak pieces to anterior positions of the area pellucida elicited secondary brain tissue development. However, no isolated nodes were grafted. In addition, the developmental stages of embryos or graft positions were not precisely determined; in those studies, it was argued that there were significant differences between the avian nodes and amphibian organizers, a notion recently re-evaluated from modern perspectives (Martinez Airas and Steventon, 2018)].

First, the development of mCherry-labeled node grafts at various positions of host embryos was compared with that of a homotopic node graft (*n*=2) (Fig. S7). The homotopically grafted nodes extended the AME tissue anteriorly during the 5-10 h after node grafting, and the AME further developed into the anterior-most PP and narrower ANC (Fig. S7A). In contrast, the PNC, with a pointed posterior end, started to develop only ∼8 h after node grafting, distinguishing AME and PNC development by the timing, direction and shape of tissue extension. Ectopically grafting the node anterior to the host node AP level (*n*=13) resulted only in anterior extension of the AME, which further developed into the PP and ANC (Fig. S7B,C), while producing a cell clump at the posterior end. In contrast, upon grafting the node at a level posterior to the host node (*n*=6), no AME tissue developed, but posterior extension of the PNC occurred after 8 h (Fig. S7D,E). These observations indicated that (1) the node develops both the AME and PNC only at the original node position; and (2) node grafts at anterolateral sites develop only AME tissue, whereas node grafts at posterolateral sites develop only PNC tissue.

Next, the development of host-derived tissues in response to node grafting outside the brain precursor region was investigated (Fig. S8A). The embryos were stained for neural development (SOX2, green) and quail-derived tissues (magenta) 16-18 h after node grafting (st. 9-10) (Fig. S8B).

**Fig. 5.**
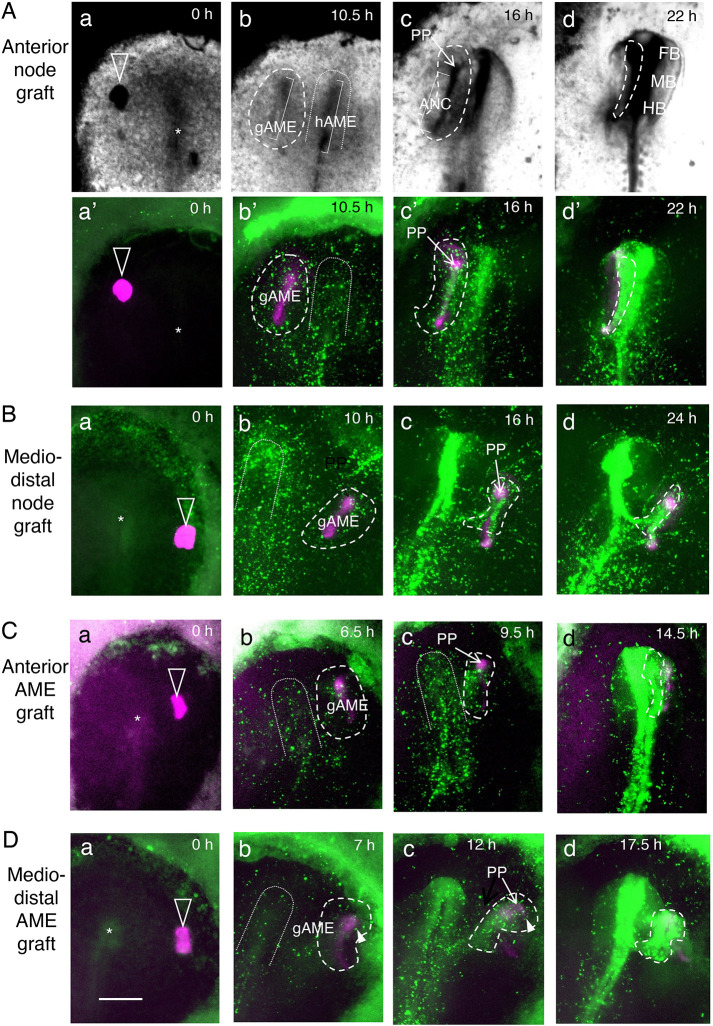
**Live imaging of the grafted node/AME and gathering of SN-EGFP-labeled host epiblast on the gAME.** The anterior is at the top. Unfilled arrowheads indicate the node/AME grafts (magenta); asterisks indicate the host nodes. Dashed lines encircle the epiblast cells gathering around the gAME and forming secondary brain tissues. The inverted U-shapes in dotted lines indicate the primary neural plate regions at the prospective and forming stages. (A) Grafting a st. 4 Japanese quail node at an anterior level. (B) Grafting a quail node at a lateral position of the host node level. (C) Grafting a st. 5 quail AME at an anterior level. (D) Grafting a st. 5 quail AME at a lateral position of the host node level. Excerpts from Movies 5-8. The periods in the culture are indicated in panels Aa-Ad. Brightfield images (Aa-Ad) show cell density changes (darker for a higher density). (Aa′-Ad′) Fluorescence images of graft-derived tissues (magenta) and SN-EGFP-labeled epiblast cells (green). In B-D, only fluorescence images are shown. (a,a′) The stage when the quail nodes/AMEs were grafted. (b,b′) The gAME elongated and elicited convergence of surrounding epiblast cells (encircled by dashed lines). (c,c′) The gAME tissue differentiated into the PP (indicated by an arrow) and ANC (covered by SN-EGFP fluorescence). The head precursors that converged on gAME started to form secondary brain tissues (encircled by dashed lines). (d,d′) The secondary brain (encircled by dashed lines) fuse to the primary brain at all AP levels (A,C) or at the posterior end (B,D). In Ad, the FB, MB and HB portions of the primary brain are indicated. Arrowheads in Db and Dc indicate the cells of area opaca origin contributing to the secondary brain. Scale bar: 500 µm.

The quail node grafted in the anterior embryo area (Fig. S8Aa-Ac,C) elicited the development of secondary brain tissues (Fig. S8Ba-Bc, *n*=13). The secondary brain developed separately from the host brain (Fig. S8Ba, *n*=4) or fused to the host brain depending on their graft positions (Fig. S8Bb, *n*=5; Fig. S8Bc, *n*=4). More proximal node grafts than shown here resulted in fusion of the secondary brain tissues along their entire lengths to the host brain, as shown in Fig. 5A. In all cases of anterior node grafting, the quail node-derived AME underlay the secondary brain tissues (Fig. S8Ba-Bc). The secondary brain portions tentatively assigned by their morphological features (positioning, width and bulging) are indicated in Fig. S8Ba-Bc and appeared to depend on the AP level of the node graft position (Fig. S8Aa-Ac).

In contrast, posterior node grafting resulted in graft-derived PNC development, in association with the host spinal cord (Fig. S8Bd, *n*=4) or in isolation (Fig. S8Be, *n*=3). A host-derived narrow strip of neural tissue aligning the graft-derived PNCs in the latter was speculated to be the secondary spinal cord floor plate (FP), although this was not characterized further.

As summarized in Fig. S8C, grafting in the anterior embryo region (anterior to the horizontal dashed line) elicited the development of secondary brain tissues, whereas grafting in the more posterior region resulted in PNC self-differentiation without eliciting brain development. The node graft-derived development of the AME (developing into the PP and ANC) or PNC was supported by the histological analysis of cross-sections (Fig. S8D,E).

Graft-dependent secondary brain development was specific to st. 4 nodes. The grafted st. 5 nodes, lacking the potential to develop into the AME (Fig. S2), primarily self-differentiated into the PNC and somites regardless of the graft position (Fig. S9).

### Live imaging of anterior epiblast migration after node/AME grafting indicates that head precursor gathering on the AMEs is the first step of brain tissue development

The above observations indicated that the nodes grafted in the anterior embryo region developed into the AME (Fig. S7) and that only under these conditions did the secondary brain tissues develop (Fig. S8). To investigate the link between these two events, we grafted mCherry-expressing quail node in the anterior region of SN-EGFP-labeled chicken host and live-recorded their developmental changes (*n*=13, Movies 5 and 6).

**Fig. 6.**
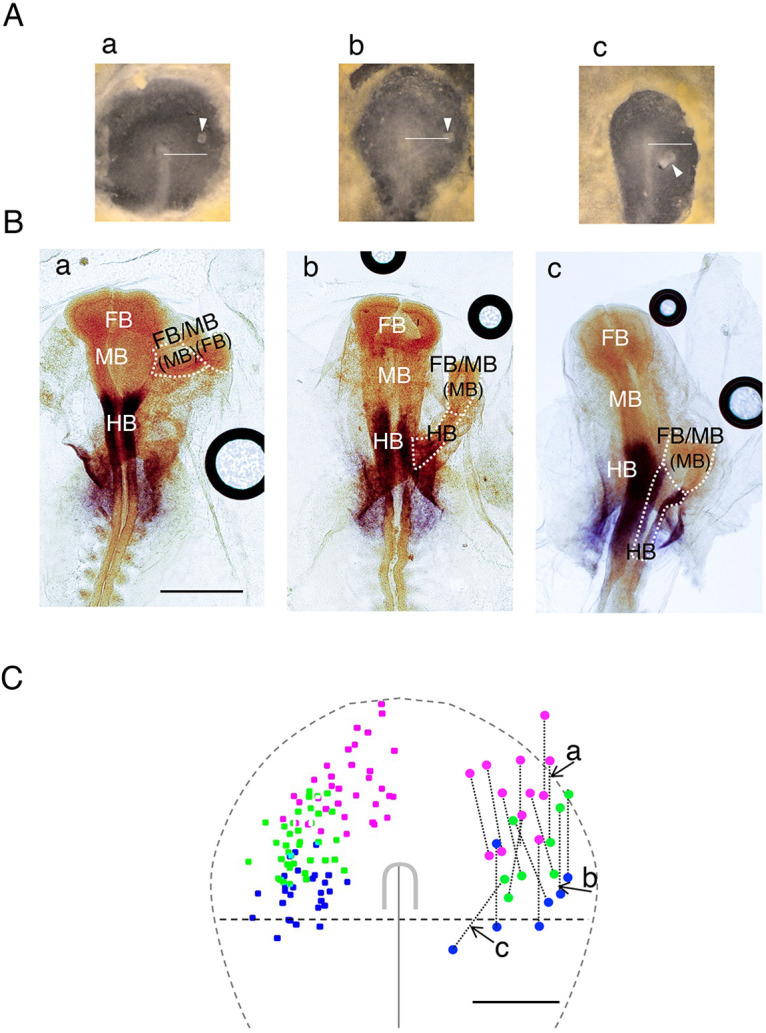
**AME graft position-dependent development of the secondary brain portions.** (A,B) Representative AME grafts at different AP levels in host embryos: (Aa) anterior to the node, (Ab) at the node level, and (Ac) posterior to the node. The horizontal bars extend 1 mm from the node center. Arrowheads indicate the grafted AMEs. (B) Embryos shown in A after ∼18 h, hybridized for *Otx2* (orange, FB/MB) and *Gbx2* (purple, HB). Primary brain portions and secondary brain portions encircled by the dashed lines are indicated by white and black letters, respectively. The tentatively assigned brain portions in the *Otx2*^+^ secondary brains on morphological bases (FB or MB) are indicated in parentheses. (Ba) Anterior AME grafting elicited secondary FB/MB development with fusion to the primary brain at the MB level. (Bb) Node-level AME grafting elicited secondary MB/HB development with primary brain fusion at the HB level. (Bc) Posterior AME grafting elicited the development of inferior FB/MB and HB, fusing with the primary brain at the HB level. (C) Left: The distribution of the head ectoderm precursors of the same axial levels as FB, MB and HB, color-coded in magenta, green and blue, respectively (Fig. 4B). Right: Color-coded dots indicating the anterior and posterior secondary brain portions tentatively assigned as in B were plotted at the anterior and posterior end positions of the elongated AME grafts, assumed to be 500 µm long. Data of 13 AME grafts are shown. The dashed lines represent the standard contour of area pellucida and the anterior epiblast posterior limit. Scale bars: 1 mm.

The case of quail node grafting in an anterior-lateral host position (*n*=5) is shown in Fig. 6A and Movie 5. Snapshots of brightfield images indicating cell density changes (darker representing a higher density) (Fig. 5Aa-Ad) and fluorescence images (Fig. 5Aa′-Ad′) are compared. The grafted node (Fig. 5Aa, Aa′) extended anteriorly to form gAME tissue synchronously with hAME (Fig. 5Ab,Ab′), which further differentiated into the PP and ANC (overlain by SN-EGFP-labeled cells) by 16 h (Fig. 5Ac,Ac′). The gAME-proximal anterior epiblast cells converged around the elongating gAME, whereas those positioned more to the right did so around the hAME (Fig. 5Ab,Ab′); they individually formed neural plates and developed into brain tissues (Fig. 5Ac,Ac′). The gAME-centered secondary brain tissue eventually fused to the host brain (Fig. 5Ad,Ad′).

Fig. 5B and Movie 6 present the case of node grafting at the host node AP level at a remote position (*n*=8), where only the fluorescence image is shown. The gAME elongated 4-10 h after node grafting, whereas the surrounding anterior epiblast converged around it (Fig. 5Bb), similar to Fig. 5Ab,Ab′. The gAME-centered secondary brain tissues (Fig. 5Bc) united to the primary brain at their posterior parts (Fig. 5Bd).

Time-lapse recordings of other embryos (*n*=7) analogous to Movie 6, where the grafted nodes stayed without AME extension when the SN-EGFP-labeled cells became detectable, show that the node grafts did not exhibit cell-gathering activities. Thus, the node graft-derived gAME rather than the grafted node provoked the convergence of anterior epiblast cells to initiate secondary brain development.

To confirm this conclusion, the isolated AMEs of st. 5 mCherry transgenic quail embryos were grafted at various host embryo positions (Fig. 5C,D, Movies 7 and 8) (*n*=7). The gAMEs quickly elongated anteriorly in 6 h and subsequently developed into the PP and ANC (Fig. 5Cb,Db, Fig. S10), 4-5 h earlier than when starting from the node graft. The surrounding anterior epiblast cells converged on the gAME (Fig. 5Cb,Db), and developed in secondary neural plates (Fig. 5Cc,Dc), while the host embryos were still at late st. 5 (Fig. S10, Movies 7 and 8). The secondary neural plates were structured into brain tissues after st. 7 of host development (∼12 h from st. 4; Movies 7 and 8; Fig. 5Cc,Dc), which was eventually fused to the host brain along the entire length of the secondary brain (Fig. 5Cd) or at the posterior end (Fig. 5Dd), similar to the case after node grafting (Fig. 5Ad′,Bd).

As summarized in Fig. S10, the local convergence of anterior epiblast cells onto the AME and subsequent neural plate formation proceeded according to the gAME development schedule, occurring 4-5 h earlier when starting from AME grafts compared with node grafts. An analysis of the event timing indicated the following. (1) By late st. 4, when SN-EGFP-labeled epiblast cells became detectable, all grafted st. 5 AME tissues had elongated and gathered proximal epiblast cells (*n*=7), indicating the competence of st. 4 epiblast cells to respond to gAME. (2) At the same developmental stage, many grafted nodes persisted without AME extension; they did not gather epiblast cells (e.g. Movie 6, *n*=7) despite the competence of the host epiblast, indicating that the nodes lack the activity to elicit local epiblast convergence.

When the epiblast gathering on gAME leading to the secondary neural plate development (encircled by dashed lines) occurred next to the forming primary neural plate (inverted U in dotted lines) (*n*=14) (Fig. 5A,C), these brains eventually fused along their lengths. In contrast, when these neural plates were remote, they developed into two separate anterior heads that fused at the secondary brain posterior end (*n*=12) (Fig. 5B,D).

During the secondary brain development from the neural plate (st. 6) to the neural tube (st. 8), the primary and secondary brain axes approached each other (Fig. 5). This process was analyzed using embryos developing two separate brains (Fig. S11). In these embryos, at st. 8 (*n*=26), the primary brain axis (H) formed an angle of 15.6±6.5 (s.d.) degrees to the immediately posterior trunk axis (T) with the vertex facing the secondary brain (Fig. S11A). This axis bending occurred because the H axis was pulled towards the secondary brain axis (G) (Fig. S11Ba-Bc). The short tracks of SN-EGFP-labeled cells relative to one of the axes G, H and B (the axis of brain-separating head ectoderm; Fig. S11Aa) were analyzed using the embryo data from Fig. 5B,D and Movies 6 and 8 (Fig. S11Ba′,Ba″,Bc′,Bc″). The analysis indicated that folding the two neural plates into narrower neural tubes and bringing the proximal head ectoderm precursors in the overlying cell layer caused the progressive proximation of the primary and secondary brain axes. On the assumption that an initially 400 µm-wide primary neural plate at st. 6 (Fig. 3B) is folded into a 160 µm-wide neural tube at st. 8, and that head ectoderm strips of 160 µm wide at both sides move to cover the neural tube (Fig. S1C), a shortening distance of 280 µm between the primary and secondary brain axes was predicted. The occurrence of the same events in a 240 µm-wide secondary neural plate added 170 µm to the distance shortening, thereby accounting for the observed axis distance shortening by 450 µm, from 1120 µm to 670 µm (Fig. S11Ba,Bc). The region of head ectoderm precursor intervening the two approaching heads remained still during the process (Fig. S11Ba″,Bb″). In the AME graft-elicited secondary brain development, structuring the brain tissue at st. 7 also contributed to the brain axes proximation (Fig. S11C).

To address the question of whether anterior epiblast cells gathering on gAMEs involved enhanced cell proliferation, node-grafted embryos were labeled using 5-ethynyl-2′-deoxyuridine (EdU) (Fig. S12). gAMEs did not alter the proliferation rate of proximal epiblast cells that contributed to secondary brain development.

### Head precursor regionality at the AME graft positions determines the secondary brain portions

The observations reported in Fig. S8 suggested that the secondary brain portions that develop from the anterior epiblast cells depend on the position of node or AME grafting. Therefore, the correlation of the AME graft position with the resultant brain portions was systematically investigated. Thirteen AME grafts were placed at various anterior embryo positions outside the brain precursor region (Fig. 4A). The embryos were analyzed after ∼18 h for *Otx2* (for FB and MB) and *Gbx2* (for HB) expression to assess the secondary brain portions. Fig. 6A,B shows representative embryos with AME grafts at different AP levels (Fig. 6A) and the resultant secondary brain portions (Fig. 6B). Anterior AME grafting resulted in secondary FB/MB development, with fusion to the host brain at the MB level (Fig. 6A,Ba). AME grafting at the node level resulted in well-developed secondary FB/MB and HB portions fusing to the host at the HB level (Fig. 6A,Bb). In contrast, grafting slightly posterior to the node resulted in the development of inferior FB/MB and HB fusing to the host at the HB level (Fig. 6A,Bc), confirming the AME graft position-dependent development of secondary brain portions.

Time-lapse recording of seven AME grafts, including those in Fig. 5C,D, indicated that AME grafts of 279±28 µm (s.d.) in initial length elongated anteriorly to 514±62 µm (s.d.) after grafting. Therefore, we assumed a model in which AME grafts elongate ∼500 µm from the graft posterior end and surrounding anterior epiblast cells gather at the elongated AME. The *Otx2*-expressing secondary brain portions were tentatively assigned, using their morphological features as FB (anteriorly positioned with lateral bulging) and MB (posteriorly positioned and thinner). These assigned anterior and posterior portions of the secondary brains were color-coded (FB, magenta; MB, green; HB, blue) and marked on the anterior and posterior ends, respectively, of the grafted AME maps (Fig. 6C, right). These plots were very similar to those of the head ectoderm precursor distribution continuing to the host FB, MB and HB precursors (Figs 4B and 6C, left). This result strongly supported a model in which the secondary brain portions develop in a manner that reflects the given regionalities of the anterior epiblast.

### Integration of the results of the present study with earlier observations defines a brain portion potential map covering the broad epiblast area

**Fig. 7.**
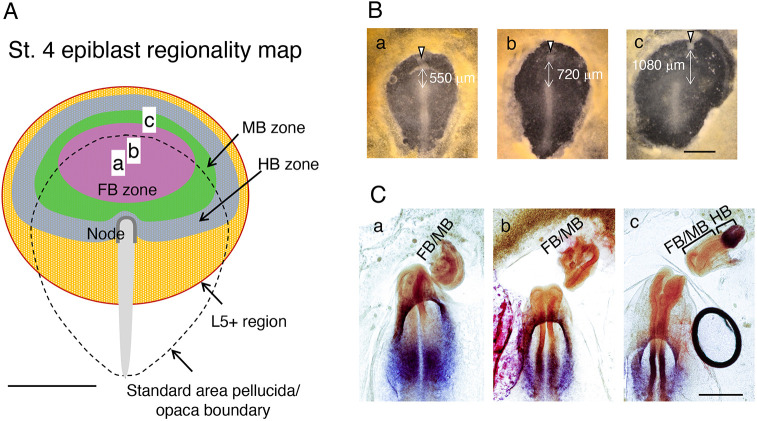
**Regionality map of the st. 4 epiblasts constructed using available data.** (A) Distribution of the head tissue developmental potential of anterior epiblast at st. 4. The developmental potential extends crossing the area pellucida boundary to the L5^+^ domain of the area opaca (Streit et al., 1997). The zones for the individual brain portion developmental potential are drawn using pale colors. Data shown in Figs 4 and 7, and earlier reported data (Dias and Schoenwolf, 1990; Foley et al., 1997; Knoetgen et al., 2000; Storey et al., 1992) are considered. a-c indicate the AME graft positions in Fig. 7B. (B) Embryos with AME grafts close to the area pellucida anterior limit (arrowheads). (C) The secondary brain tissues in the embryos shown in B after 18 h, hybridized for *Otx2* and *Gbx2* expression. Embryos a and b with the grafts in the FB potential zone developed the *Otx2*-expressing secondary FB/MB tissues. Embryo c with AME grafting at a position remote from the node developed secondary HB tissue in addition to the FB/MB. Scale bars: 1 mm (A,B); 500 µm (C).

The shape and size of the area pellucida, presumed to be the embryonic precursor region, varied markedly among embryos (Figs 6A, 7B, Fig. S8A), whereas embryos developed equivalently. The trajectories of SN-EGFP-labeled epiblast cells indicated that the cells travel across the anterior area pellucida/opaca boundaries in many embryos (e.g. cell tracks in dashed ovals in Fig. 1Ab). The epiblast cells originating from the area opaca contributed to the secondary brain tissues (e.g. Fig. 5D, white arrowheads). These observations suggested that the proximal region in the anterior area opaca epiblast can be regarded as an extension of the embryogenic epiblast. Streit et al. (1997) reported the following interesting observation concerning the responsiveness of the area opaca. The area opaca region that produced host-derived brain tissues in response to node grafting was limited to the L5 antigen-expressing domain, extending anteriorly from the area pellucida (Fig. 7A). The area opaca region with L5 expression appears to overlap with the region where SN-EGFP-labeled cells seamlessly migrate from and to the area pellucida (Fig. 1Ab, Movies 1, 2, 6, 8).

The grafting of st. 4 nodes in the L5-expressing area opaca region on the lateral sides results in MB and HB tissue development (Storey et al., 1992), indicating that this region bears MB and HB regionalities. Knoetgen et al. (2000) reported that the grafting of nodes from mouse or rabbit embryos in the anterior area pellucida/area opaca boundary of st. 4 chicken embryos results in secondary brain tissue development, with FB tissue in the area pellucida and HB tissue in the area opaca. Dias and Schoenwolf (1990) also reported that node grafting in an anterior pouch of the germinal crescent elicits the formation of secondary brain tissues with FB, MB and HB portions, which were in the head-to-head orientation with the host brain. In contrast, Foley et al. (1997) reported that only the secondary FB portion develops following AME grafting in a lateral pouch.

Considering these previous observations and extending the data in Figs 4C and 6C, we propose the head precursor regionality map of st. 4/5 epiblasts shown in Fig. 7A. This model predicts that the epiblast area immediately anterior to the brain precursor region (Fig. 4A) possesses FB regionality. In contrast, more remote anterior regions have MB and HB regionality in this order.

To test this model, AME grafts were placed at the anterior-most position of the area pellucida oriented with the anterior end pointing to the node. The distances between the AME grafts and the node varied considerably among embryos. Three such embryos with graft positions a-c in Fig. 7A,B were allowed to develop for 18 h and hybridized for the analysis of *Otx2* and *Gbx2* expression (Fig. 7C). Grafting the AME within the FB zone (Fig. 7A) in embryos a and b (Fig. 7B) resulted in the development of *Otx2*-expressing FB/MB neural tissues (Fig. 7C).

Embryo c in Fig. 7 had an exceptionally vast anterior epiblast field, and the AME graft was 1.08 mm anterior to the node, crossing the FB, MB and HB zones (Fig. 7A). Remarkably, this exceptional setting resulted in the development of the *Gbx2*-expressing HB portion, in addition to the FB/MB portions (*Otx2*^+^), in strong support of the st. 4 epiblast regionality map (Fig. 7A).

## DISCUSSION

### Long-distance migration of anterior epiblast cells towards the head axis to develop into brain and head ectoderm tissues

The live imaging of SN-EGFP-labeled epiblast cells demonstrated the long-distance collective migration of epiblast cells from a broad area starting at mid st. 5, causing cell convergence around the forming head axis (Figs 1 and 2A). The cell migration rates ranged from 70 to 110 µm/h and peaked at late st. 5 (Fig. 2B). The direction of cell migration towards the head axis in the bilateral areas a,a′ in Fig. 2 was diagonal to the axis, whereas that in the axial zones b,b′ was roughly parallel to the axis (Fig. 2, Fig. S3).

Detailed analysis of these cell tracks allowed delineation of the brain portion precursor map at st. 4/5 (Fig. 4A), revising the map drawn by Fernández-Garre et al. (2002), as detailed below. The head ectoderm precursors of different AP levels were distributed as an external extension of the precursors' abutting brain portion precursors (Fig. 4B,C), which was crucial information when considering the brain portion developmental potential of the anterior epiblast cells.

### Revision of the brain precursor map

Fernández-Garre et al. (2002) proposed a brain portion precursor map of st. 4 chicken epiblast by the homotopic grafting of labeled epiblast disks ∼125 µm in diameter in the endoderm-ablated region of host embryos and detecting the labeled cell distributions after 24 h. Their map was drawn assuming that the brain portion precursors were distributed identically and with defined boundaries. However, this study showed that the anterior limits of the brain precursors varied between 210 and 450 µm anterior to the node tip level among the embryos. Schoenwolf and Alvarez (1991) also reported a case in which a homotopic graft 500 µm anterior to the node contributed to the FB (Fig. 4A, asterisk).

The most significant differences were found along the midline. The map drawn by Fernández-Garre et al. (2002) had only FB precursors immediately anterior to the node and a 150-µm indentation in the anterior boundary of the FB precursors, which was 400 µm from the node on both sides. However, this study showed the presence of MB precursors and some HB precursors between the FB precursors and the node in all embryos (Fig. 3B, Fig. S5, Fig. 4A) and no midline indentation in the FB precursor boundary (Fig. 2B, Fig. S5). The local endoderm ablation adopted in epiblast grafting (Fernández-Garre et al., 2002) could have affected anterior AME extension (Fig. 1B, Fig. S7) and axial brain precursor migration (Fig. 2, Fig. S3), thus causing a head axis-specific anomaly in the operated embryos.

### The brain developmental potential of the non-brain precursor region of anterior epiblasts is manifested by local convergence on ectopic AME tissues

Node grafting in the anterior embryo region elicited gAME extension without PNC development (Fig. S7). Only under these conditions did secondary brain tissues develop (Fig. S8). With this information, the grafted node/AME labeled with mCherry and SN-EGFP-labeled epiblast cells was recorded live. Regardless of whether derived from a grafted node or after direct grafting, ectopic gAME tissues positioned outside the brain precursor region (i.e. within the head ectoderm precursor region) elicited the local gathering of surrounding anterior epiblast cells, which then developed into secondary brain tissues.

A significant difference between the node graft-derived AME and the directly grafted AME was that epiblast cell gathering occurred 4-5 h earlier with the latter starting in st. 4 hosts. During this period, the grafted node did not exhibit epiblast-gathering activity (Fig. S10). This observation confirmed that the AME rather than the node elicits local epiblast gathering.

To determine whether known diffusible factors, particularly those emanating from the AME, are involved in these epiblast cell-gathering steps, COS7 cells overexpressing LEFTY1, CERL1 (nodal antagonists), DKK1 (Wnt antagonist) or NOGGIN (BMP antagonist) were grafted underneath the anterior epiblast. None of these factors or their combinations mimicked the AME in eliciting local cell convergence or promoting brain tissue development (data not shown), only adding to the list of factors ineffective for promoting neural development reported by Linker and Stern (2004).

### Regional distribution of the brain portion developmental potential in the epiblast

Ectopic gAME tissues placed in the anterior embryonic region always elicited secondary brain development. The brain portions that developed in the secondary brains (Fig. 6C) reflected the epiblast regions (Fig. 4C) that elongated AME tissues traversed. The results indicate that the head ectoderm precursors and brain precursors of the same laterally extending region at st. 4/5 (Fig. 4C) share the potential to develop into the same brain portions.

The results of grafting the node/AME in the anterior embryonic region (Figs 5, 6, Figs S8, S11) indicate the following model: (1) The anterior epiblast cells are bipotent in terms of being able to develop into the brain or head ectoderm. (2) Once the AME tissue develops, regardless of the host or graft origin, the AME-proximal anterior epiblast cells converge on the AME; the centrally positioned cells develop into the brain tissues, whereas those more distally positioned develop into the head ectoderm. (3) The brain portions in the secondary brain tissues reflect the original regionality of the convergent anterior epiblast cells.

Considering previous data on node graft-dependent brain portion development (Dias and Schoenwolf, 1990; Foley et al., 1997; Pinho et al., 2011; Storey et al., 1992; Streit et al., 1997), combined with the brain portion potentials summarized in Fig. 6C, a model of the entire brain portion potential map was drawn (Fig. 7A). Regions around the standard area pellucida/opaca boundary (Fig. 7A) may develop into area pellucida or area opaca, contingent on embryo-dependent variations. This model was further supported by grafting an AME at a far anterior position eliciting secondary HB portion development (Fig. 7B,C). The brain portion potential map in Fig. 7A should provide a standard guide for further analyses of epiblast-derived developmental processes.

## MATERIALS AND METHODS

### Fertilized eggs

Non-transgenic chicken and quail fertilized eggs were obtained from local breeders. Fertilized eggs of *mCherry*-transgenic Japanese quail [TG(PGK1:H2B-chFP)] (Huss et al., 2015) were provided by the Avian Bioscience Research Center at Nagoya University as part of the National Bioresources Program. The embryos were staged according to Hamburger and Hamilton (1951) and Ainsworth et al. (2010). Animal experimentation was performed in accordance with the guidelines of Nagoya University and Kyoto Sangyo University.

### Random EGFP labeling of the epiblast by electroporation of Supernova vector cocktails

A st. 4 chicken embryo was isolated using a ring-shaped filter paper support attached to the vitelline membrane, and placed between a pair of 2×2 mm electrodes of 6 mm distance in the orientation of the ventral side upward facing the anode, using a set-up described previously (Kondoh and Uchikawa, 2008; Uchikawa et al., 2003, 2017). The following mixture of vector DNAs was prepared (Fig. S1B): 1 µg/µl pK031 (TRE-CRE-pA) (Mizuno et al., 2014), 0.5 µg/µl pK038 (CAG-LoxP-Stop-LoxP-GFP-IRES-tTA-pA) (Mizuno et al., 2014) and pT2K-CAG-tTA (provided by Y. Takahashi, Kyoto University, Japan) at 0.4 µg/µl (Figs 1-3, 5C,D) or 0.1 µg/µl (for more sparse cell labeling in Fig. 5A,B; Fig. S1C), delivered at a volume of ∼2 µl between the epiblast and vitelline membrane, and electroporated with four pulses of 8 V with 50 ms duration and 1 s intervals using CUY21SC (Nepa Gene). The embryos were placed on agar-albumen medium (Uchikawa et al., 2003) and cultured in the orientation of the ventral side upward. The embryo images were recorded in tiff format with 10 min interval time-lapse (except for Movies 5 and 6, which were recorded at 25 min intervals) using a humidified chamber at 38°C under an AF6000 inverted microscope (Leica). Linear level adjustment of color channels and pseudocolor operations were performed using Fiji software (Schindelin et al., 2012). The tiff stacks were encoded into mov-formatted movies using in-house developed software. The movies were produced at a rate of 6 frames/s, showing 1 h of real time in 1 s in Movies 1-4, 7 and 8.

### Trajectory analyses of SN-EGFP labeled cells

The image sequences were first smoothened using a median filter (r=1) and Gaussian blurring (σ=1.0). To manage the variation in fluorescence intensity between the frames and fluorescence image radius among the labeled cells, software implemented in C was developed in-house to perform the following procedures. (1) Local fluorescence maxima were collected as the candidate cell positions of labeled cells. (2) These cell positions were ordered according to their peak intensities. (3) Donut-shaped masks of 2 pixel width were prepared with 2 pixel radius increment steps spanning 4-20 pixels to collect intensity distribution data around the maxima. (4) These donut masks were applied to every peak to test the significance of the peak position intensity compared with peripheral regions using a ROKU algorithm (Kadota et al., 2003). The peaks that passed this test were registered as the ‘cells’. (5) The test was repeated by increasing the donut radius to collect all possible cells with various fluorescence diameters. (6) The information on the labeled cell positions was transformed using an R-script to align the horizontal axis of the forming head and keep the st. 5 node position at its posterior end. Then, trajectories of the SN-EGFP-labeled cells were drawn over image sequences using software in C developed in-house (available upon request) (e.g. Figs 1Ab,Bb, 2A, Fig. S3]. This protocol successfully tracked the labeled cells over tens of frames (covering several hours) but created gaps when the fluorescence signal of a cell became lower (close to background noise) in consecutive flames. When more extended cell tracking was needed (e.g. Fig. 3C), the tracks were generated by visually monitoring cell positions on the frames processed in the following way. The cell position data were generated to include low-fluorescence spots allowing the appearance of fluctuating background spots and overlaid on the original fluorescence image, allowing the choice of consistent tracks by an ImageJ (Schneider et al., 2012) plugin developed in-house.

### Tracing the brain portion boundaries at st. 9 of SN-labeled embryos back to st. 6 and st. 5

The brain precursors at st. 6 were distributed in the bilateral epiblast zones 200 µm from the embryo axis and between the forming anterior ectodermal ridge and the original node position, as confirmed by repeated forward tracking. These labeled brain precursors became a part of the folding neural plate and closing neural tube, where fluorescence from the majority of the ventrally located precursors in the neural tube gradually diminished, and fluorescence-labeled dorsal neural tube cells and overlying ectoderm cells, derived from immediately outside the st. 6 brain precursor field, were detectable at st. 9. At this stage, the anterior part of forebrain often bent ventrally in culture, as exemplified by Fig. 3Ab. The embryo was fixed immediately after the SN-EGFP recording and hybridized *in situ* for *Otx2* and *Gbx2* expression. The flat-mounted *in situ* hybridization image was then aligned in register with the image of the SN-EGFP-labeled embryo using somite positions and other morphological features as guides. The FB/MB boundary where anterior FB bulging occurs, the MB/HB boundary marked by the *Otx2*/*Gbx2* transition, and the HB/SC boundary defined by the posterior end of neural tube *Gbx2* expression were copied on the st. 9 SN-EGFP images. These boundaries were traced back, maintaining the relations to brightly labeled landmark cells to st. 8. Backtracking from st. 8 to 6, more centrally located cells gaining marked fluorescence provided additional landmarks for brain portion boundaries, and the portion boundaries were traced back to st. 6 (Fig. S4). SN-EGFP-labeled cells in the FB, MB and HB compartments of the brain precursor field at st. 6 were individually traced back to determine their positions at st. 5 (Fig. 3B, Fig. S5).

### Node and AME grafting

To graft nodes, stage-matched chicken host and Japanese quail node donor embryos, both at st. 4, were isolated using the ring filter support and placed on agar-albumen support used in embryo culture. The donor node was excised as a rectangular tissue block by an operation using a sharpened tungsten needle from the ventral side and transferred to the host embryo (Figs S2A, S7, S8). A size-matched rectangular well was formed in the host embryo, into which the donor node was inserted.

To isolate the AME from st. 5 Japanese quail embryos, a rectangular incision along the AME contour was made from the ventral side, leaving the epiblast intact. Then, 5 µl of 2.5% Pancreatin (Wako-Fujifilm) in HEPES-buffered saline (Qiu et al., 1998) was added to the incision and kept at room temperature for <1 min to detach the AME from the epiblast. The stripped AME was rinsed and inserted between the epiblast and the ventral layer of a st. 4 chicken host embryo through an incision at an anterior aspect of grafting in the ventral layer.

To record early cell convergence responses of the host epiblast, Supernova vector electroporation was performed at early st. 4 of the recipient embryo, and after 2 h of incubation when the host was still at st. 4, the graft operations described above using an mCherry-transgenic donor were performed.

### WISH

Embryos were fixed with 4% paraformaldehyde (PFA) in PBS at 4°C overnight and kept in methanol at −20°C. WISH was performed as described by Henrique et al. (1995). The *Gbx2*, *Otx2* and *Sox2* probes were synthesized using relevant templates (Katahira et al., 2000; Uchikawa et al., 2011) (provided by H. Nakamura, Tohoku University, Japan, and M. Uchikawa, Osaka University, Japan), labeled by digoxigenin (DIG) or fluorescein isothiocyanate (FITC), and used at 500 ng/ml. *Gbx2* or *Sox2* hybridization was detected by purple color development using 1/2000 dilution of alkaline phosphatase-conjugated anti-DIG antibodies (Roche) and with 340 µg/ml nitroblue tetrazolium chloride and 175 µg/ml 5-bromo-4-chloro-3-indolyl-phosphate in 0.1 M Tris-HCl, pH 9.5, 0.1 M NaCl, 50 mM MgCl_2_ and 0.1% Tween 20. Subsequently, *Otx2* hybridization was detected by orange color development using a 1/500 dilution of alkaline phosphatase-conjugated anti-FITC antibodies (Roche) and HNPP Fluorescent Detection Set (Roche). The embryos were mounted in RapiClear 1.52 (SunJin Lab) and photo recorded using an Axioplan 2 microscope (Zeiss).

### Whole-mount immunostaining of embryos

Chicken embryos of st. 8 to 10 were fixed in 4% PFA in PBS for 1 h at 4°C. The fixed embryos were stored in methanol at −20°C. The embryos were placed sequentially in 75%, 50% and 25% methanol, in 0.1% Tween 20 in PBS, and finally in 10% Blocking Reagent (Roche), 0.5% Triton X-100 and 0.1% Tween 20 in PBS, in which immunoreactions were performed. Primary antibodies were rabbit anti-SOX2 (Epitomics, AB5603, 1/500) and mouse QCPN (quail cell marker antibody, Developmental Studies Hybridoma Bank, Iowa University, 1/100), and reacted at 4°C for 36 h (anti-SOX2) or 18 h (QCPN). After rinsing the embryos in 0.1% Tween 20 in PBS four times at room temperature, the secondary antibodies (Alexa 488/568-labeled donkey anti-rabbit/mouse IgG; Abcam, ab150061/ab175700) were applied at 1/1000. Fluorescence images were taken using an M165 FC fluorescence stereomicroscope (Leica).

### EdU labeling of node-grafted embryos

Embryos, isolated with a ring filter paper support and with a node graft at st. 4, were incubated at 38°C in the orientation of the ventral side upward. After 6 or 8 h, the embryos were flipped to dorsal side facing upward, the vitelline membrane was pierced, 200 µl of 40 µM EdU was added to the epiblast side of the embryos, and the embryos were incubated in this orientation for a further 2 h. Only the epiblast cells were labeled using this procedure. We confirmed that these dorsal-side-upward cultures allowed embryo development at least up to st. 15, similar to ordinary ventral-side-upward cultures. After fixation with 4% PFA for 15 min at room temperature, the embryo specimens were processed for Alexa 488 fluorescence using the EdU Proliferation Assay Kit (Abcam) and counterstained with 1 μg/ml 4′,6-diamidino-2-phenylindole (DAPI). Fluorescence images were taken using an FV3000 inverted laser microscope (Olympus).

## Supplementary Material

Supplementary information

Reviewer comments
