# Supplementary material for: Live imaging of avian epiblast and anterior mesendoderm grafting reveals the complexity of cell dynamics during early brain development
Source: Development. 2022 Mar 28;149(6):dev199999. doi: 10.1242/dev.199999 (PMC9017232; doi:10.1242/dev.199999)
Supplement: Supplementary information [file develop-149-199999-s1.pdf]

Figure S1

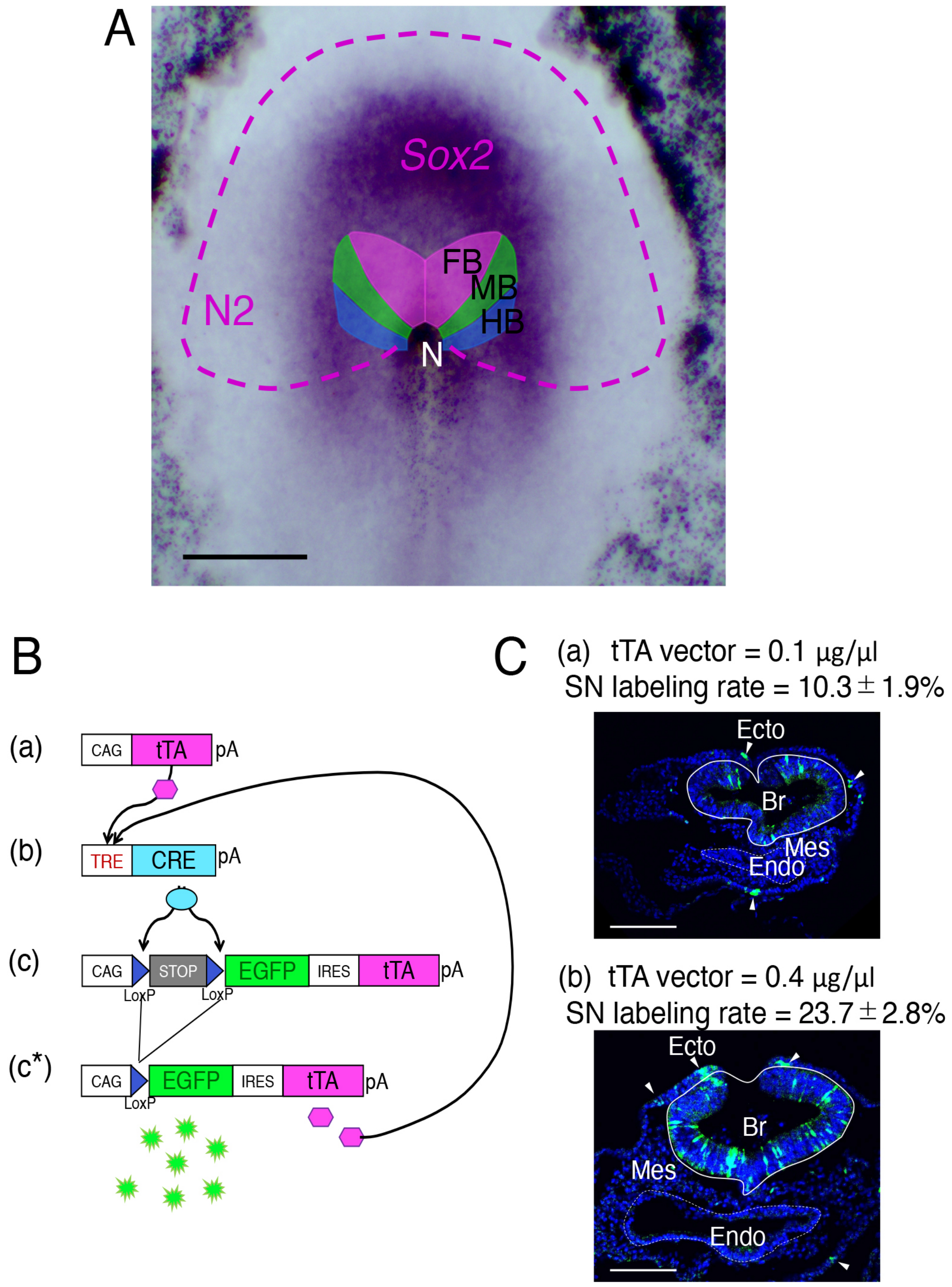
A
Sox2
N2
FB
MB
HB
N
B
(a)
CAG
tTA
pA
(b)
TRE
CRE
pA
(c)
CAG
STOP
EGFP
IRES
tTA
pA
LoxP
LoxP
(c*)
CAG
EGFP
IRES
tTA
pA
LoxP
C
(a) tTA vector = 0.1 µg/µl
SN labeling rate = 10.3 ± 1.9%
Ecto
Br
Mes
Endo
(b) tTA vector = 0.4 µg/µl
SN labeling rate = 23.7 ± 2.8%
Ecto
Br
Mes
Endo

Fig. S1. Observations on the regionalities of chicken embryo epiblast and the Supernova-EGFP (SN-EGFP) cell labeling method. (A) Comparison of the *Sox2* expression in a chicken embryo anterior epiblast at early st. 5, when the AME has just started anterior extension, with the brain precursor map reported by Fernández-Garre et al. (2002). The *Sox2* expression was detected by in situ hybridization. The broken line indicates the outer limit of the N2 enhancer activity, which activates epiblastic *Sox2* expression (Iwafuchi-Doi et al., 2011). N node, FB forebrain, MB midbrain and HB hindbrain. The anterior is at the top. Bar, 500 µm. (B) The vectors composing the Supernova system. (a) An expression vector (tTA vector) provides an amount of tTA [tetracycline-controlled transactivator (Gossen et al., 1995)] in electroporated cells. (b) TRE (tetracycline-responsive element)-dependent CRE expression vector. When tTA binds to this element, CRE recombinase is synthesized. (c) An EGFP–tTA joint expression system using an IRES (internal ribosome entry site) sequence driven by the CAGGS enhancer-promoter complex (CAG) (Niwa et al., 1991). This vector's coding sequences remain untranscribed unless the transcriptional termination sequence (STOP) cassette flanked by the LoxP sequences is excised. (c*). The tTA proteins expressed from the STOP-excised vector (c) feed-forwardly, together with exogenous tTA, activate vector (b), producing CRE recombinases more abundantly. This process continues until all vector (c) sequences become free from the STOP cassette, leading to the full EGFP expression in electroporated cells. (C) Cross-sections of SN-EGFP-labeled brain tissues in embryos electroporated with the vector cocktail at st. 4 from the epiblast side. The cryosections were immunostained for EGFP (green) and stained with DAPI (blue, for nuclei), according to Iida et al. (2020), showing SN-EGFP labeling of epiblast-derived tissues, brain (Br, encircled), and head ectoderm (Ecto, some indicated by arrowheads). However, endoderm (Endo, encircled by broken lines) and head mesenchyme (Mes) were not labeled to a significant extent. (a) and (b) Representative embryos electroporated with tTA plasmid at concentrations of 0.1 and 0.4 µg/µl, which showed tTA vector concentration-dependent increase of SN (EGFP) labeling rate. Statistical significance of the labeling rate differences was examined in the following way. Two SN-EGFP-labeled embryos at each tTA vector concentration were chosen to assess the fraction of SN-EGFP-labeled cells in brain tissues (including those with faint labeling) using head-level 10

Development • Supplementary information

cross-sections per embryo. Individually, embryos electroporated with 0.1 μg/μl tTA vector showed the labeling rates 9.5±1.7 (SD) % and 11.1±1.8%, whereas those with 0.4 μg/μl tTA showed the rates 24.4±3.0% and 23.1±2.7%. One-way ANOVA comparing 4 embryos ($F = 111$, $P < 0.01$) followed by post-hoc tests (e.g., Tukey HSD) indicated $P < 0.01$ for any embryo pairs with different tTA vector inputs, confirming the significance of tTA vector concentration-dependent increase in the SN-EGFP labeling rate. In (C), the averaged labeling rates of two embryos are indicated. Bars, 100 μm.

Development • Supplementary information

Figure S2

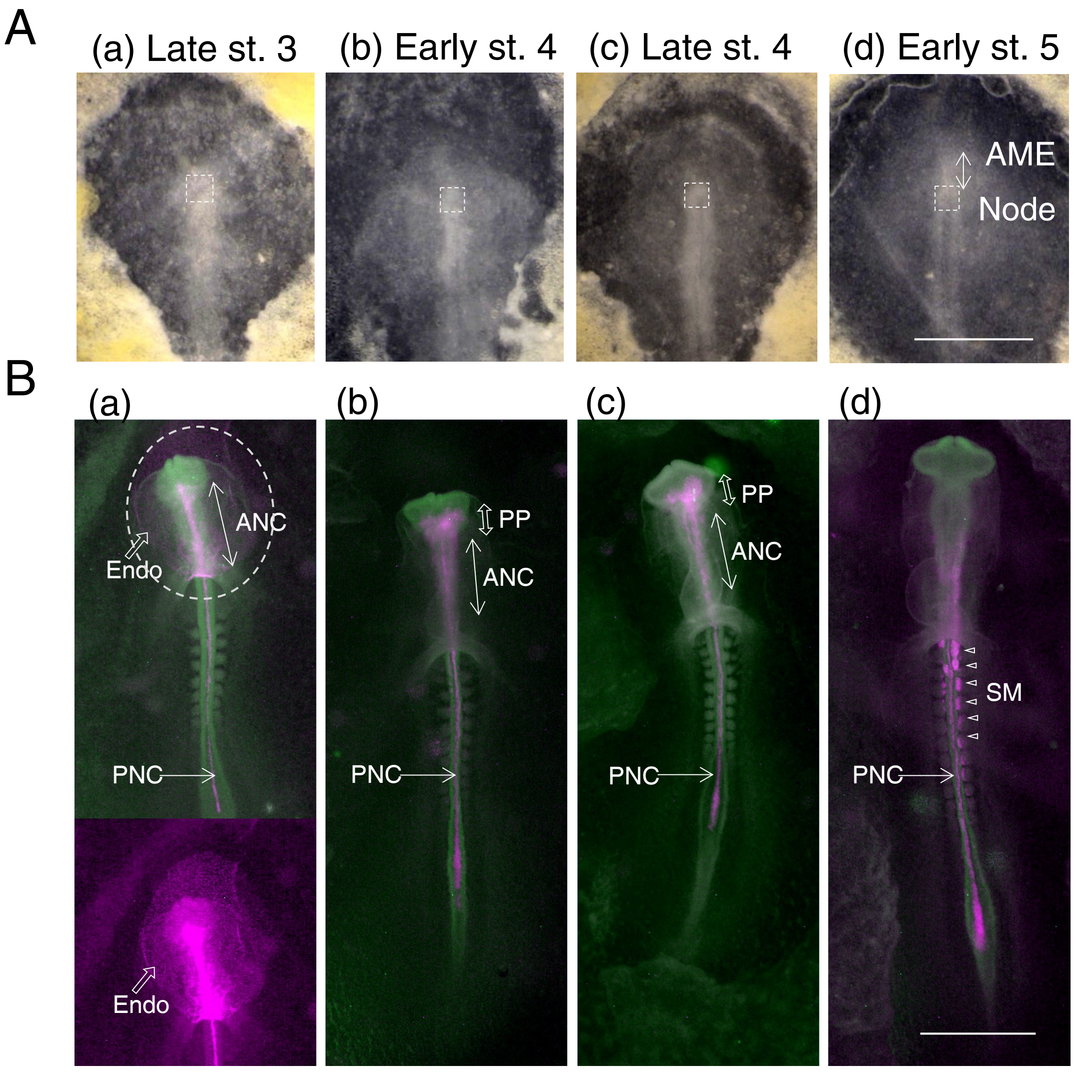
A
(a) Late st. 3
(b) Early st. 4
(c) Late st. 4
(d) Early st. 5
AME
Node
B
(a)
(b)
(c)
(d)
ANC
Endo
PNC
Endo
PP
ANC
PNC
PP
ANC
PNC
SM
PNC

Fig. S2. Development of st. 4 nodes solely into the anterior mesendoderm and posterior notochord. (A) The Japanese quail donor embryos in the ventral view at respective stages and the node tissues to be excised indicated by the broken rectangles. In st. 5 embryos, the anterior mesendoderm (AME) protrudes from the node (the double arrow). Bar, 1 mm. The anterior is at the top. (B) The host chicken embryos that received the respective node grafts. The embryos were cultured for ~16 h, fixed, immunostained in whole-mount for quail-derived tissues (magenta) and SOX2 expression (green). Ventral views are shown. (a) An embryo that received a st. 3 node graft. The grafted node developed into the endoderm (Endo), encircled by the broken line, the anterior notochord (ANC), and the posterior notochord (PNC, indicated by an arrow). The lower panel shows only the anti-quail immunofluorescence to more clearly indicate the development of the quail node-derived endoderm. (N = 2). (b) and (c). Embryos that received Japanese quail node grafts at early st. 4 (N = 3) and late st. 4 (N = 2). In both cases, the grafted node developed into full AME consisting of the prechordal plate (PP) and ANC, besides PNC. (d) An embryo that received a st. 5 node. (N = 3). The grafted node did not give rise to any AME tissue but developed into the medial portions of the somites (SM with open arrowhead), besides PNC. Bar,1 mm.

Development • Supplementary information

Figure S3

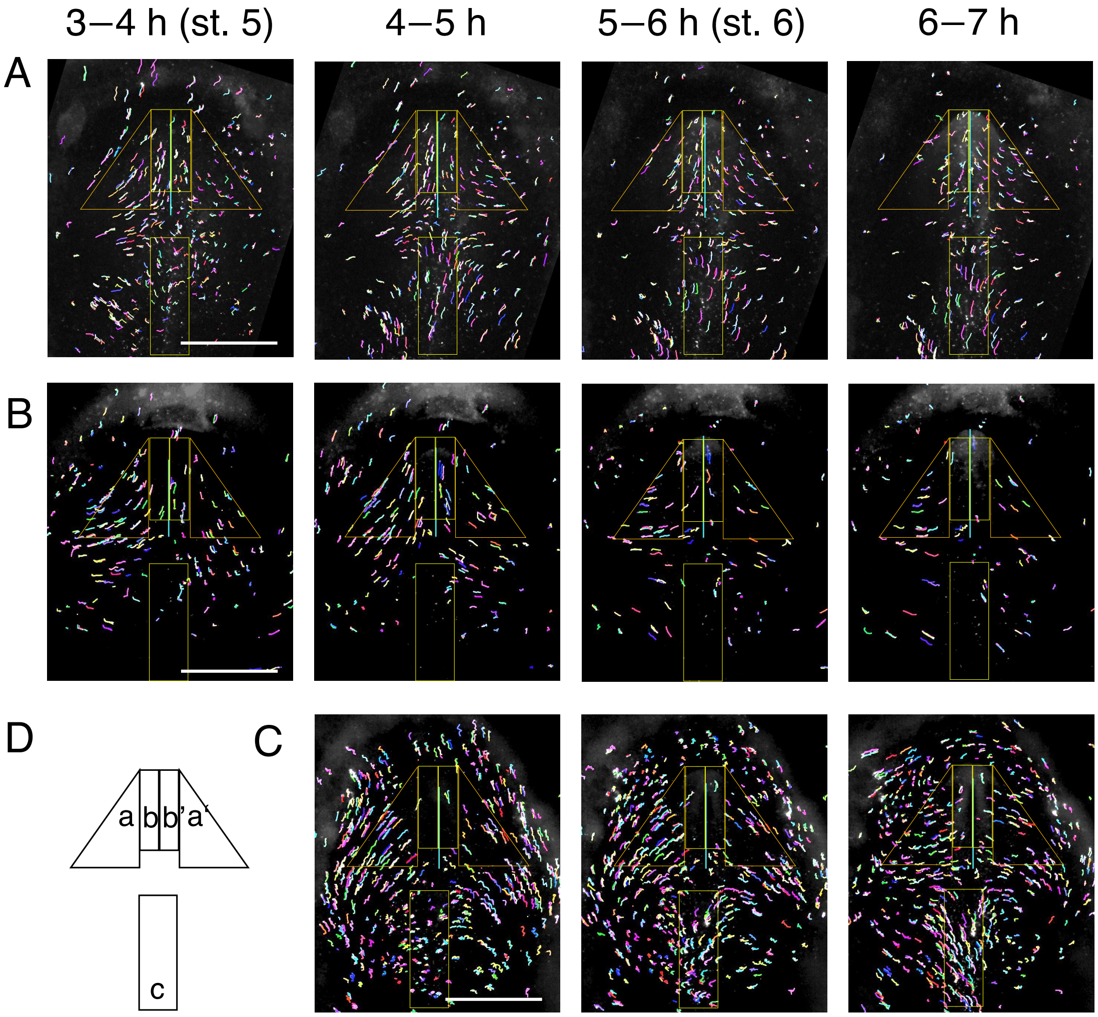

E

Embryo region	Measurements	Hours after entering st. 5<br>3–4 h (st. 5)	4–5 h	5–6 h (st. 6)	6–7 h
(a)	Average migration rate (μm/h) ± SD	**80.2 ± 18.2**	**93.8 ± 19.6**	**81.9 ± 12.8**	**71.4 ± 17.7**
	Average angle of cell migration (degree) ± SD	44.4 ± 14.5	43.7 ± 18.5	51.0 ± 20.4	60.0 ± 11.3
	Track numbers	87	106	99	100
(b)	Average migration rate (μm/h) ± SD	**78.2 ± 13.4**	**86.8 ± 23.1**	**75.5 ± 18.7**	**70.2 ± 21.5**
	Average angle of cell migration (degree ± SD	9.7 ± 14.7	14.3 ± 13.8	17.9 ± 13.2	22.6 ± 16.0
	Track numbers	38	47	39	31
(c)	Average migration rate (μm/h) + SD	**72.0 ± 18.6**	**105.9 ± 14.8**	**77.0 ± 23.9**	**86.8 ± 24.7**
	Average angle of cell migration (degree) ± SD	–3.5 ± 37.8	–5.4 ± 10	4.0 ± 20	5.0 ± 15.2
	Track numbers	22	22	57	75

Development • Supplementary information

Fig. S3. Migration tracks of SN-EGFP-labeled epiblast cells from st. 5 to 6 at 1 h intervals. Three embryos differing in high-density SN-EGFP-labeled areas were used for the analysis to cover the entire anterior epiblast field with tracks: (A) Dense labeling in the head axis-proximal zone, (B) intermediate zones, and (C) more distal zones. Embryos (A) and (C) also had high-density labeling in the posterior field. (D) The three epiblast regions with specific cell migration patterns relevant to neural tissue development were analyzed for cell migration rates and directions (angles from the embryo axis) to gain averages of three embryos. (a)(a’) Bilateral right triangular regions where epiblast cells migrate toward the head axis to be precursors for the brain and head ectoderm tissues. (b)(b’) Bilateral belt zones along the head axis, each 200 µm wide, where presumptive brain precursors migrate anteriorly. (c) The 400 µm posterior belt zone where epiblast cells of postnode levels migrate posteriorly. Embryo (C) started to exhibit SN-EGFP labeling after 2 h of entering st. 5; hence, data for the 2 to 3 h span was not scored. Bar, 1 mm. Comparison of the changes in regions (a)(a’) among the three embryos indicated that labeled cells in embryo (A) reach the (b)(b’) zone fast, followed by those of embryos (B) and (C) in sequence, reflecting the differences in the initially labeled regions. Besides, we have observed variations in the timing to start cell migrations and the migration angles between embryos and even between the embryo sides. In embryo (A), the start of cell migration in the (a’) (right) region was at least 1 h delayed compared to the (a) (left) region. The posterior cell migrations in region (c) started ~2 h of st. 5 in embryo (A), but it started only after 4 h of st. 5 in embryo (C). The cell migration rates (µm/h) and angle (degrees from the embryo axis) were measured in the regions indicated in (D) in all specimens. Data for (a’) and (b’) were flipped horizontally and compiled with regions (a) and (b) data to the average for the three embryos. (E) Summary of the statistics of the analysis of migration rates and directions. The average migration rates ranging from 70 to 110 µm/h changed moderately but with relatively small standard deviations (SD). The changes in the migration rates between the adjacent intervals in a specific region (a–c) were statistically significant ($P < 0.001$, Mann-Whitney U test). In contrast, the changes in the average directions (angles from the embryo axis) were accompanied by large SDs, reflecting the variations in a region, between the embryo sides, and among the embryos. These statistics are summarized in Figure 2(E).

Figure S4

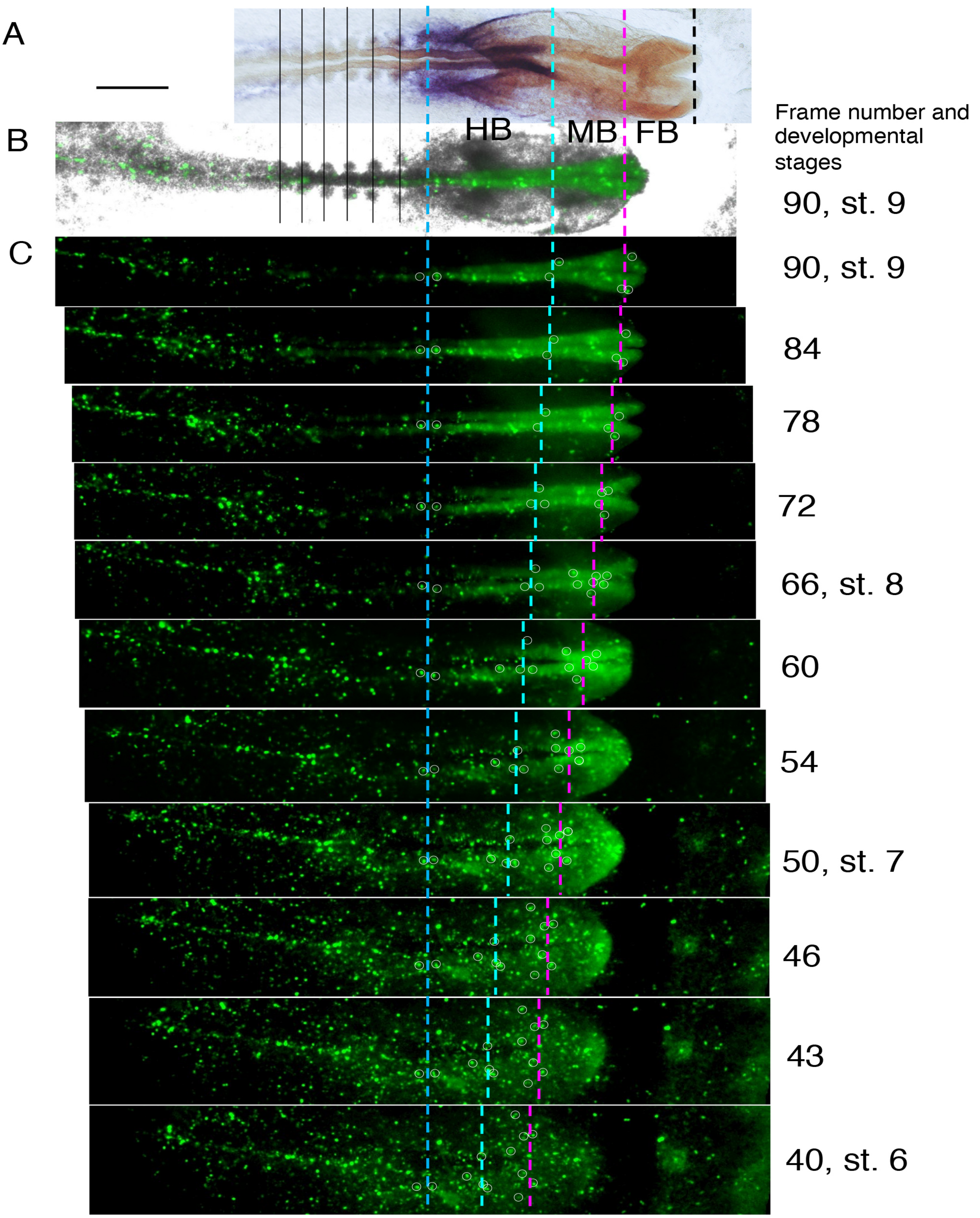

Development • Supplementary information

Fig. S4. Tracking back the brain portion boundaries from st. 9 to st. 6 using SN-EGFP-labeled cells. (A) Immediately after the termination of SN-EGFP recording, the labeled embryos were fixed and hybridized in situ for the analysis of *Otx2* (expressed in the FB and MB, orange) and *Gbx2* (expressed in the HB, purple) expression. The FB/MB boundary was determined as the posteriormost position of lateral expansion characteristic of the FB. (B) The hybridized embryo images were aligned to the last frame of the bright field/SN-EGFP composite image. In the embryo shown in Figure 3, the last frame was the 90th at st. 9 with an imaging interval of 10 min. This embryo showed ventral curling of the FB region, which occasionally occurs in culture, whereas the hybridized specimen was mounted flat, with extension of the FB portion. The two embryo images were aligned in register using the somite positions and other morphological features as guides. These boundaries were marked on the SN-EGFP image or the 90th frame. (C) These boundaries (HB/spinal cord, blue; HB/MB, cyan; and MB/FB magenta) were tracked back using SN-EGFP fluorescence image frames to earlier stages guided by landmark labeled cells encircled in the panels eventually to st. 6, as described in the Materials and Methods section. Note that the panel sequences show that ventral curling of the FB portion started after st. 7. In this figure, the frame excerpts are arranged so that the boundaries can be followed in these still images. In the actual procedure, however, positional changes of the landmark labeled cells were followed in all frames using a movie to accurately track the brain portion boundaries. The same procedure was used for all four embryos at st. 6 shown in Figure S5. Bar, 500 µm.

Figure S5

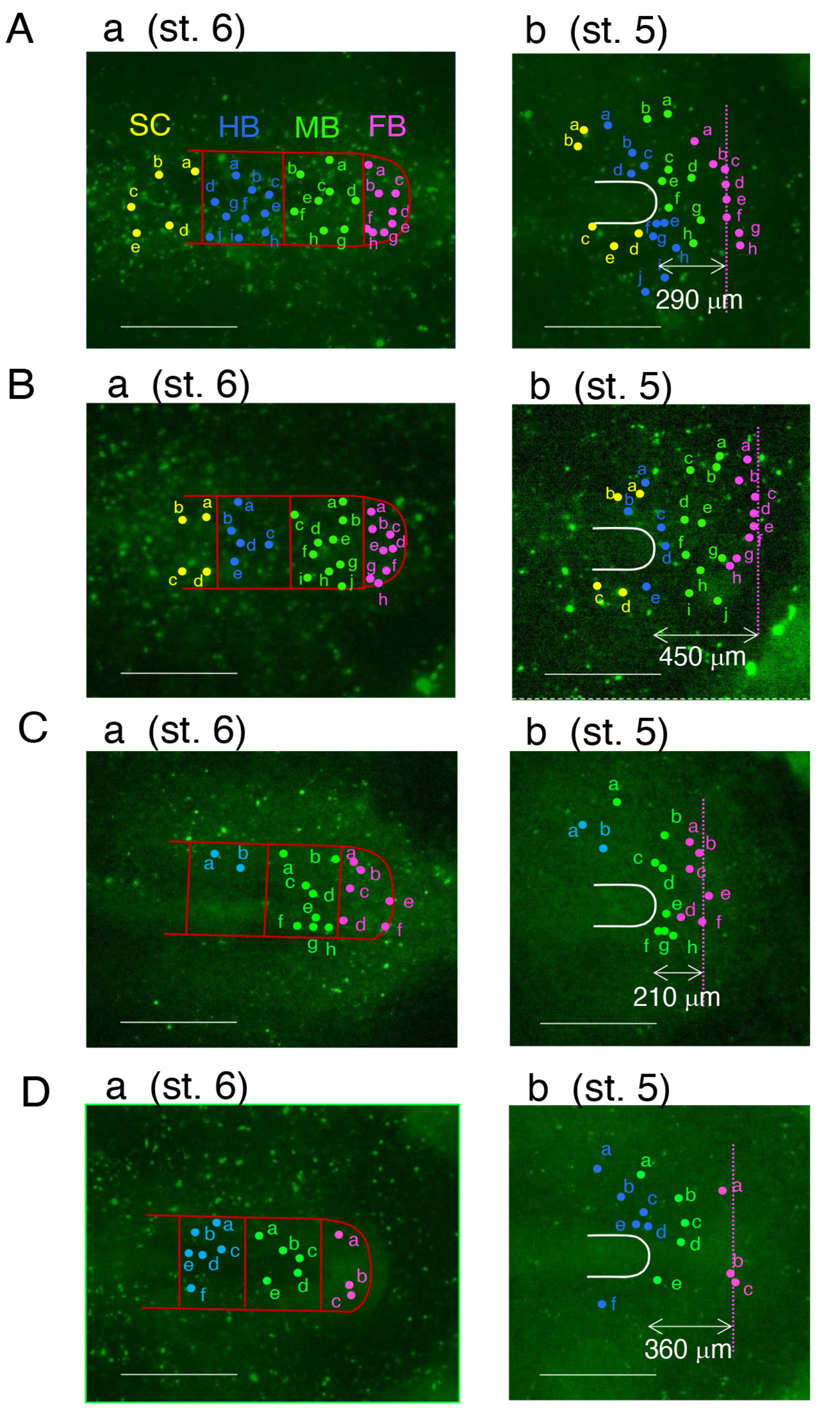

Development • Supplementary information

Fig. S5. Distribution of brain portion precursors at st. 6 and st. 5 in four different embryos, (A) to (D). (a) Brain portion precursor domains at st. 6, which were determined by backtracking the portion boundaries from st. 8/9 when the embryos were hybridized in situ for *Otx2* and *Gbx2* expression, as detailed in Materials and Methods and Figure S4. Color-coded dots indicated the SN-EGFP-labeled cells at this stage in each brain portion precursor domain; FB in magenta, MB in green, and HB in blue. Alphabetical labeling of individual cells establishes correspondences in cell positions between st. 5 and st. 6. For embryos (A) and (B), some SC precursors were also labeled as references. (b) Cell positions at st. 5 of the labeled cells in each brain portion precursor domain at st. 6. Bars, 500 µm.

Figure S6

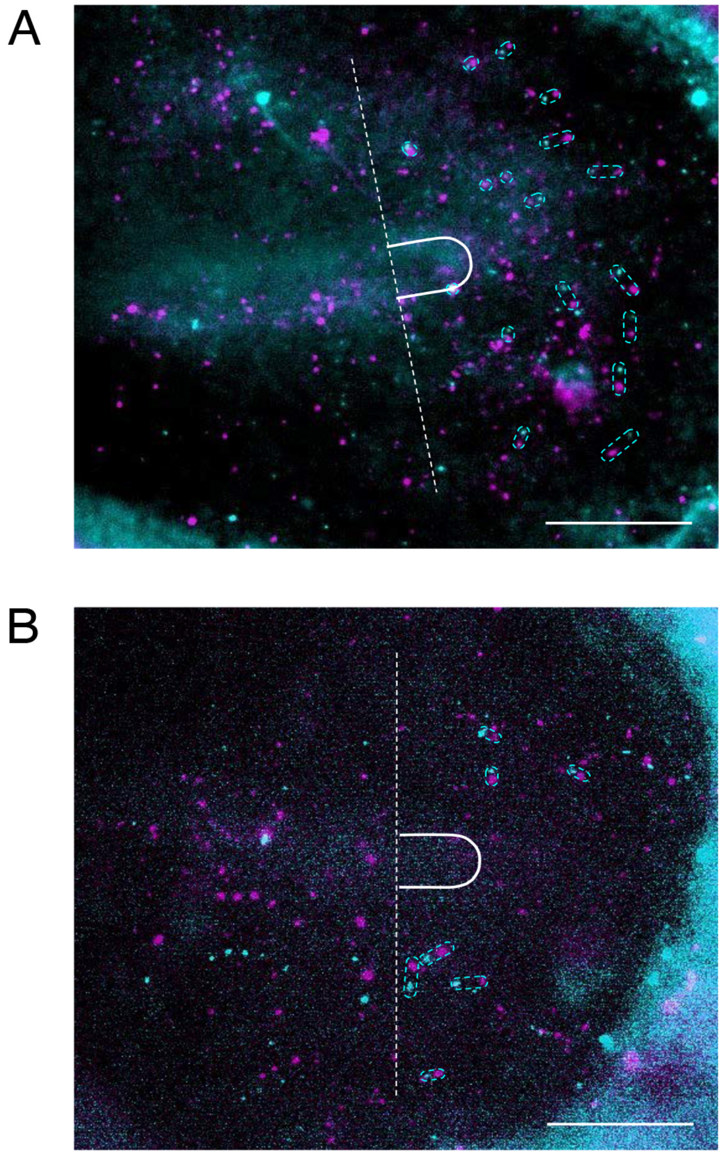

Fig. S6. Comparison of the SN-EGFP-labeled cell positions between st. 4 and st. 5 in two representative embryos. The fluorescence images at st. 4 in cyan and those in st. 5 in magenta were superimposed. 24 conspicuous spot pairs representing drifts in the cell positions anterior to the node levels (broken lines) between the stages were chosen (encircled in magenta broken lines). Measurement of their displacement distances gave an average of 39 ± 26 (SD) μm displacements in 130 min without defined orientations. Bars, 500 μm.

Development • Supplementary information

Figure S7

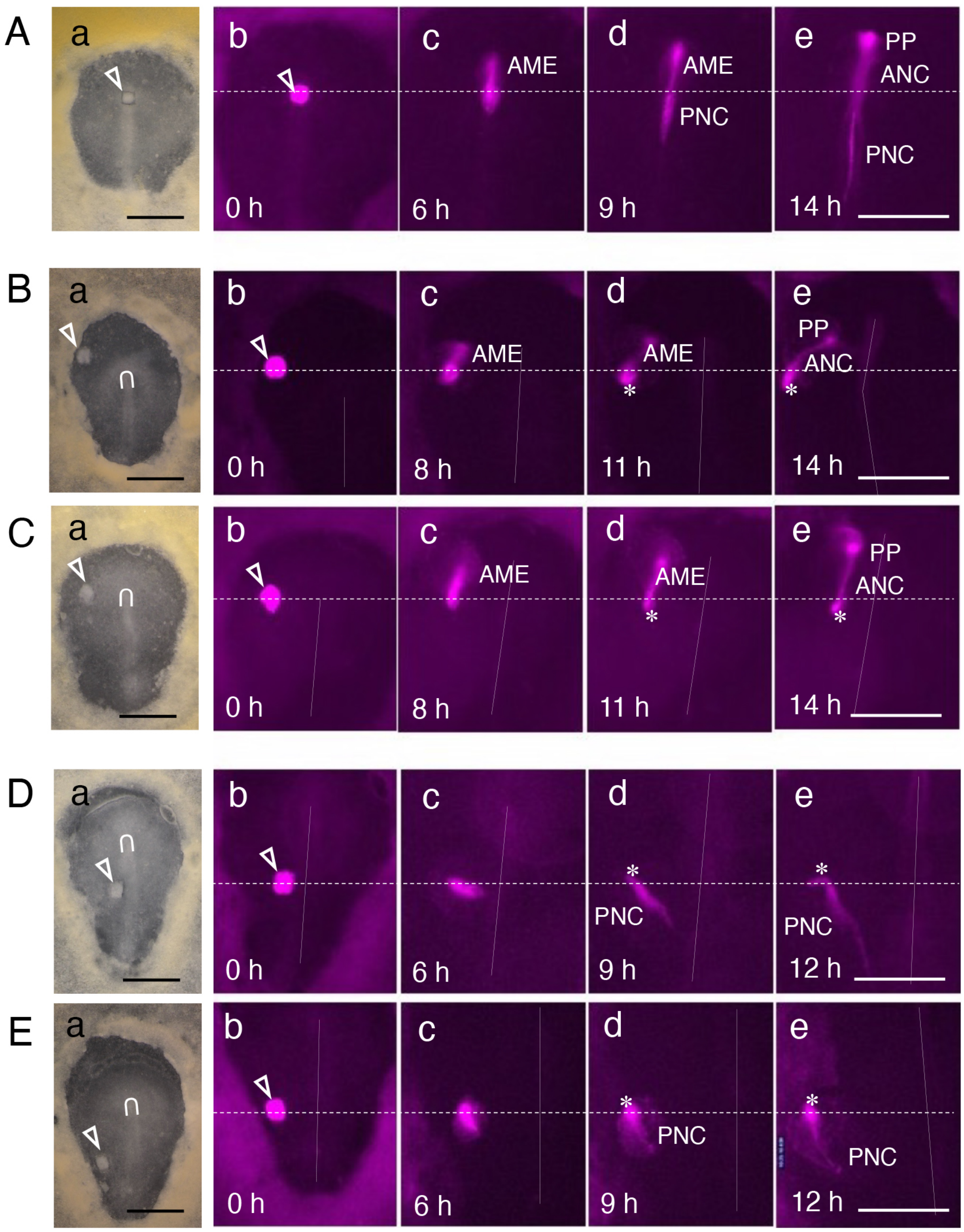

Development • Supplementary information

Fig. S7. Development of mCherry-expressing st. 4 Japanese quail nodes at various positions of chicken host embryos. The nodes were grafted in the same orientation as the host embryos; the anterior is to the top. The inverted U shapes indicate the host nodes. (a) The grafted nodes in the host embryo in the ventral view are shown to indicate their graft positions. (b) to (e). The mCherry fluorescence of tissues derived from the node grafts, recorded at the time points indicated at the panel bottoms in embryo culture. The dorsal images were flipped for the node graft to have the same laterality as (a) in the panels. The axial levels of the initial node grafts in an embryo (b) are indicated by the broken lines. Approximate embryo axes are indicated by thin lines. Bars, 1 mm. (A) A homotopic graft at the node position replacing the host node (N = 2). (b) The fluorescence of the node graft. (c) The anterior extension of AME starts around 6 h of grafting. (d) After 9 h, the posterior extension of the posterior notochord (PNC) starts, while the AME continues to extend. (e) With prolonged culture, the posterior extension of the PNC continues. (B) and (C) The node grafts at a level anterior (B) or the equivalent (C) to the host node (N = 13). (c) and (d) The anterior extension of the AME occurred at the same timing as the homotopic grafts, followed by differentiation into the PP and ANC (e). However, the PNC extension did not occur in any of the anterior node grafts, leaving cell clumps (asterisks) at the posterior end of the node-derived tissues. (D) and (E) The node grafts at the AP levels posterior to the host node (N = 6). (c) to (e) Anterior extension of node-derived tissues never occurred, leaving cell clumps at the anterior ends (asterisks). The posterior PNC extension occurred at the same timing as the homotopic node grafts shown in (A).

Development • Supplementary information

Figure S8

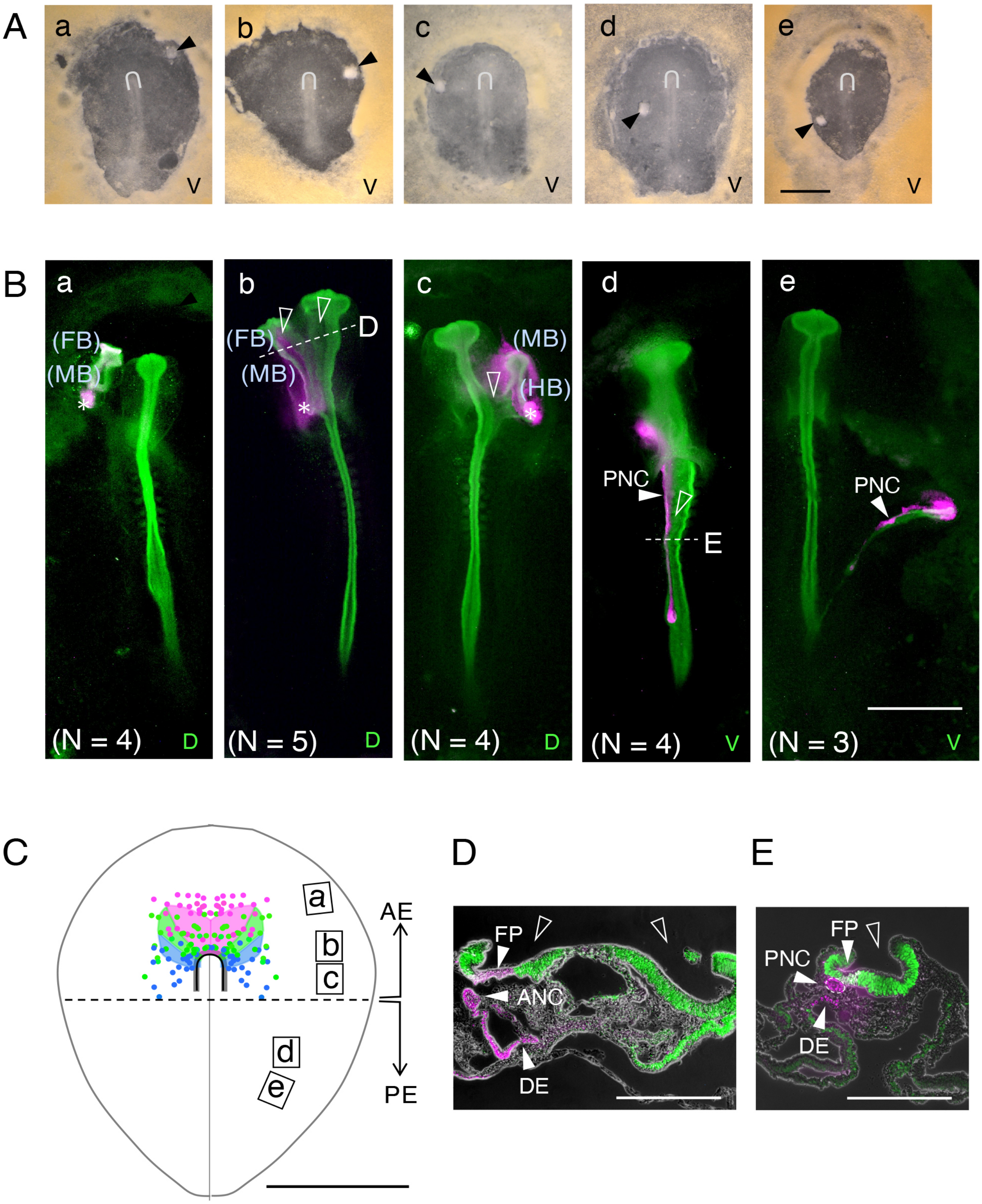

Development • Supplementary information

Fig. S8. Anterior epiblast-specific development of host-derived secondary brain tissues following st. 4 quail node grafting. (A) Japanese quail node graft positions (arrowheads) in the st. 4 chicken host. V, ventral images. The anterior, at the top. (B) Embryos after 16–20 h of quail node grafting shown in (A), immunostained for neural tissues (SOX2, green) and quail-derived tissues (magenta). D, dorsal images. The tentatively assigned secondary brain portions on the morphological basis (FB, MB, and HB) are indicated in parentheses. N, the number of analogous specimens. (a) The anterior node graft away from the midline elicited secondary brain development, separated from the host brain. (b) The secondary brain fused to the primary (host) brain at its side. The primary and secondary brain portions were defective at their dorsal sides (open arrowheads). (c) The secondary brain fusing to the host brain at the HB level. In (a) to (c) the graft-derived AME developed ventral to the brain tissue (magenta) with posterior cell clumps (asterisks). (d) and (e) The embryos where the nodes were grafted at more posterior positions, where the secondary PNC developed. In (d), the node-derived PNC was incorporated into the posterior host tissues, whereas in (e), the PNC self-differentiated from the node graft, to which a narrow host-derived strip of SOX2-expressing neural tissue was aligned. (C) A summary of the results shown in (A) and (B). The node graft positions were drawn on the schematic st. 4 embryo map showing the brain precursor distributions [Fig. 4(A)]. The broken line, the approximate posterior limit of the anterior epiblast giving rise to the secondary brain tissues. (D) and (E) Immunofluorescence images of sections of embryo (b) at level “D” and of embryo (d) at level “E” in (B), which were superimposed on the phase-contrasted images. The grafted quail node developed into the floor plate (FP), ANC/PNC, and adjacent definitive endoderm (DE), consistent with earlier observations (Catala et al., 1996; Teillet et al., 1998) that these tissues in the spinal cord originate from the same precursor pool. The present data extend these tissue relationships to the cranial level. The analysis also indicated that the close apposition of two neural tubes or integration of the node graft-derived PNC/FP into the host spinal cord [(B)(b)–(d)], resulted in the neural tube closure defects (open arrowheads). Bars, 1 mm in (A)–(C), 250 μm in (D)(E).

Development • Supplementary information

Figure S9

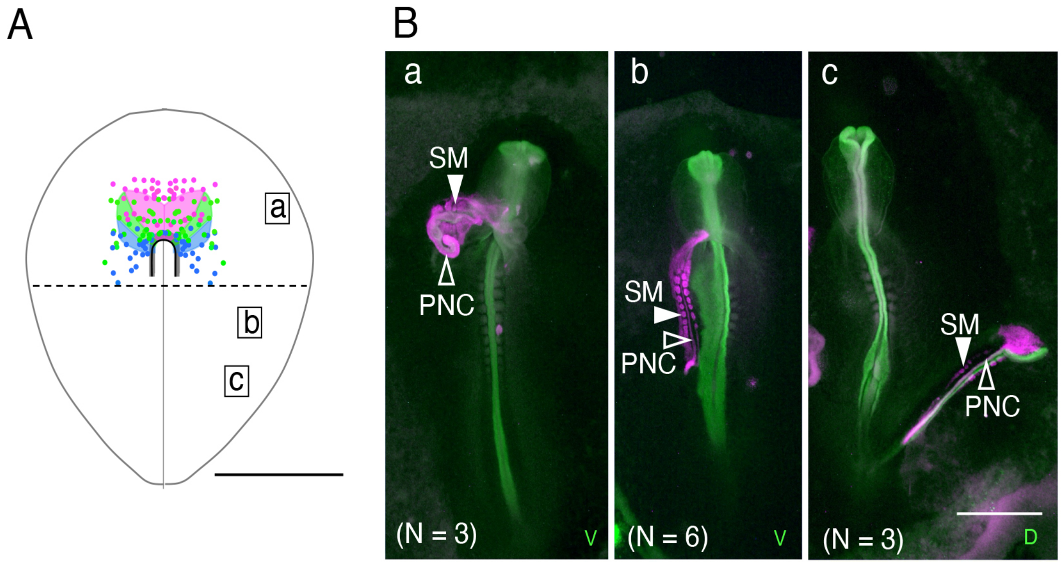

Fig. S9. The outcome of st. 5 node grafts at various anteroposterior levels of st. 4 embryos. (A) Positions of the st. 5 node grafts (a) to (c) (rectangles) in embryos presented in (B), drawn on the schematic st. 4 embryo shown in Fig. 4(C). (B) Embryos after culturing for 16–20 h, stained for neural tissue (SOX2, green) and quail-derived tissues (magenta). In all embryos, the grafted node self-differentiated into the posterior notochord (PNC) and the somites (SM). (a) The PNC and SM tissues derived from the anteriorly grafted node protruded from the host tissue. (b) Medial position graft resulted in fusion with the host tissue at the anterior end. (c) PNC and SM tissues developed from a posterior-distal node graft independent of the host tissue. A narrow strip of host- derived neural tissue associated with the secondary PNC, analogous to Fig. 4(B)(e). “N” indicates the number of immunostained embryos with similar outcomes. Bars, 1 mm.

Hornburch et al. (1979) performed grafting of nodes from st. 5 embryos based on the criteria of this study (Fig. S2) in various positions of the area pellucida. The results were similar to the st. 5 node graft data shown here.

Figure S10

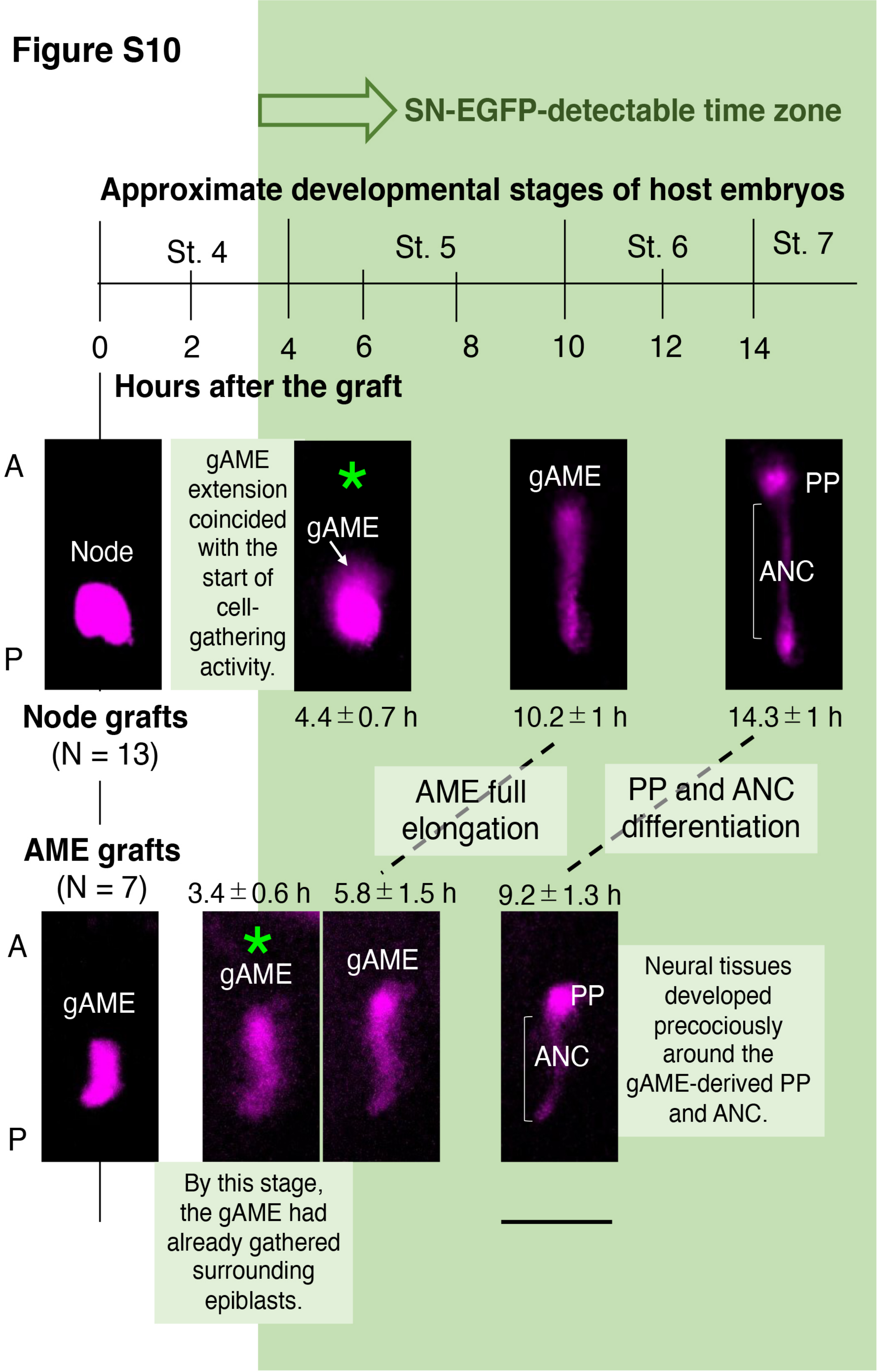

Development • Supplementary information

Fig. S10. Schematic representation of the sequence of events after the grafting of st. 4 nodes (N = 13) and st. 5 AMEs (N = 7) in early st. 4 chicken host embryos on the time scale of the host developmental stages and the hours after grafting. The host embryos were electroporated with SN-EGFP vectors, resulting in fluorescence signals around the end of st. 4 and starting the “SN-EGFP-detectable time zone.” The grafts were from mCherry-expressing transgenic quail embryos; their characteristic morphological changes are shown in magenta in fluorescence images, which are excerpts of data in Figure 5(A) (Movie 5) for the nodes and in Figure 5(C) (Movie 7) for the AME tissues. The green asterisks indicate the first moments that the cell-gathering activity of the grafted node/AME (gAME) was detected. A and P indicate the anterior and posterior sides of the grafts. Bar, 500 µm.

The grafted nodes maintained their original state with clear tissue demarcation for ~3 h and then started to extend the gAMEs, which was the first moment the cell-gathering activity of the grafted tissue could be detected. In many specimens (N=7), node grafts were still in the original state when entering the SN-EGFP-detectable time zone and did not show activity to gather cells around. Toward the end of st. 5 (~10 h after grafting), the gAME elongated, and was wider on the anterior side, and elicited convergence of proximal epiblast cells. Then, during st. 6, the AME differentiated into the wide and short PP and thin and long ANC.

The AME grafts were isolated from the st. 5 donor embryos during their elongation. When the SN-EGFP became detectable (green asterisk), surrounding epiblast cells had already gathered. This observation indicated that st. 4 epiblast cells have the potential to respond to and gather around gAME tissues. Subsequent developmental steps of the gAME proceeded on the graft-autonomous schedule, ~5 h earlier than the development of the node graft or host node. Approximately 9 h after AME grafting, when the host embryos were still close to the end of st. 5 when the neural plates were still forming, the epiblast cells that had gathered around the gAME tissue started neural tube development (Movies 7–8).

These observations indicate that the epiblast cells have a wide time window to respond to gAME tissues but not to nodes and develop into neural tissues following the time course of gAME development.

Development • Supplementary information

Figure S11

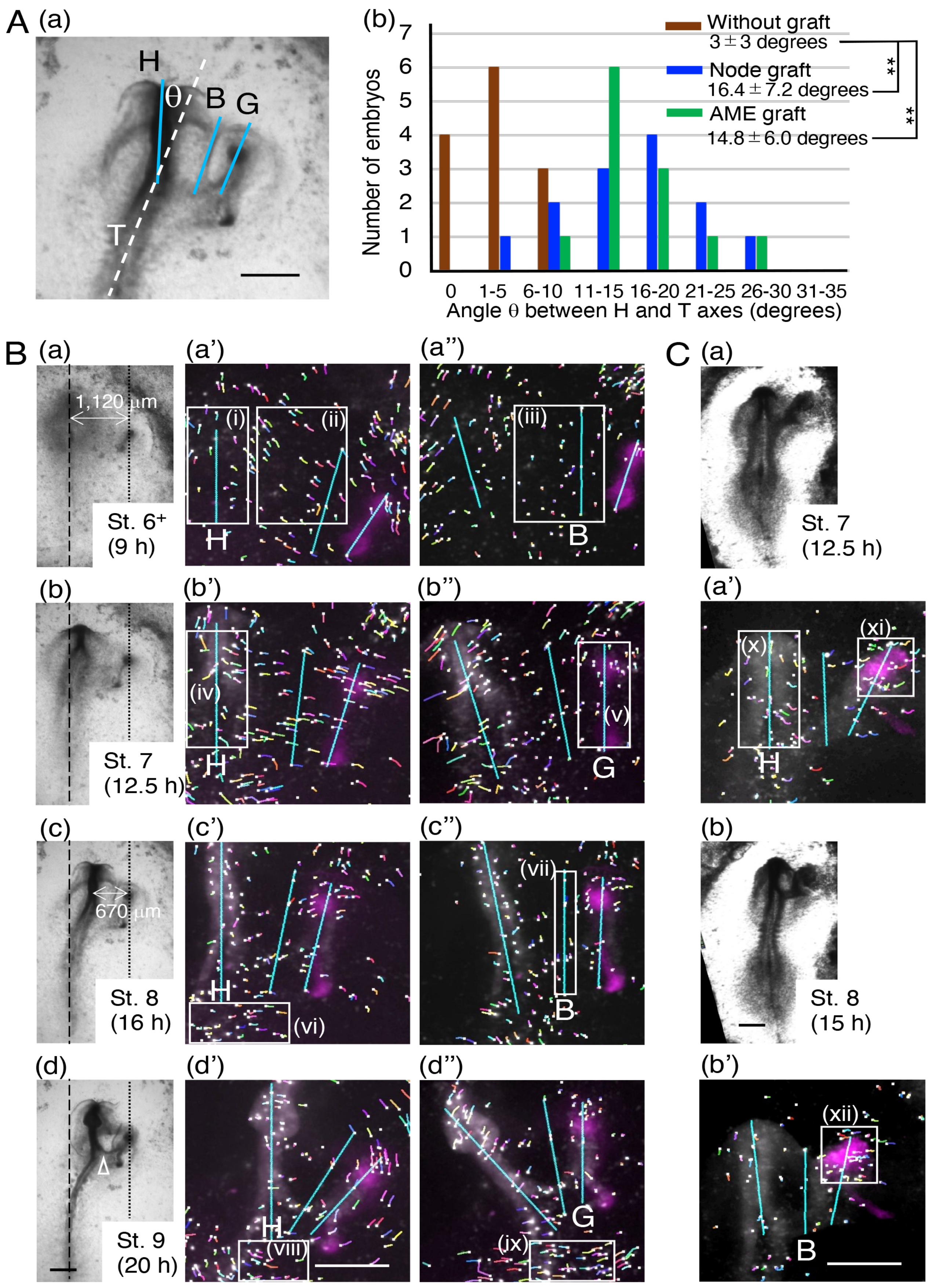

Development • Supplementary information

Fig. S11. Histogenesis of primary and secondary brains is responsible for the progressive proximation of the two brain tissues. Bars, 500 μm. (A) Definition of tissue axes used in the analysis (a) and secondary brain-dependent bending of the primary head axis relative to that of the trunk (b). (a) H, host (primary) brain axis; G, graft-dependent (secondary) brain axis; B, the axis of boundary head ectoderm partitioning the primary and secondary heads; T, the axis of the anterior trunk, with which the H axis made the angle θ. (b) Distribution of the H axis bending angle θ measured in 13 embryos of each group at st. 8. Without graft, the bending was on either side. **, $P < 0.001$ using the Mann–Whitney U test.

(B) and (C) Analyses of trajectories of SN-EGFP-labeled cells around axes H, B, and G, during the brain histogenesis stages. (i) to (xii) The areas where short tracks of SN-EGFP-labeled cells provide information concerning cell dynamics in neural tube formation and head ectoderm precursors covering or intervening the two neural tubes.

(B) An embryo with the node graft in Figure 5(B). (a)–(d) Bright-field images at stages 6+, 7, 8, and 9. The original positions of the primary and secondary brain axes at st. 6+, which were 1,120 μm apart, are indicated using broken and dotted lines, respectively. These axes became closer by 450 μm at st. 8. (a')–(d') and (a'')–(d'') Short tracks of SN-EGFP-labeled cells over a duration of 75 min drawn relative to one of the three axes, H, B, or G. The most recent cell positions are indicated in white; single white dots represent cells that remained in their position. (i) The primary neural plate at st. 6+, where the cells showed anteromedial cell convergence around the H axis (44 ± 15 μm/h). (ii) The juxtaposed cell sheet pulled toward the H axis (47 ± 12 μm/h). (iii) The cell movements relative to the B axis in the corresponding area were negligible. (iv) As the neural tube developed, the cell convergence toward the H axis became more extensive (50 ± 15 μm/h). (v) Around the G axis, the axial convergence was moderate (26 ± 5 μm/h), reflecting the smaller scale of secondary neural tube histogenesis. (vi) Cell movements in and around the neural tubes diminished at st. 8, except for their posterior ends (41 ± 13 μm/h), which pulled each other. (vii) The head ectoderm penetrating through the B axis (29 ± 7 μm/h) partitioned the primary and secondary heads. The two heads in this embryo pulled each other further at st. 9

Development • Supplementary information

(viii, 52 ± 13 μm/h; ix, 51 ± 9 μm/h) along a thick neural tissue bridge [arrowhead in (d)], creating a large θ (~40 degrees), which was observed in 3 out of the 13 node-grafted embryos.

(C) An embryo with the AME graft in Figure 5(D). (a)(b) Bright-field images at st. 7 and 8, when the unstructured secondary neural plate was reorganized into the symmetrical brain tissue (Movie 8). The tissue reorganization aligned the secondary brain with the primary brain, which was observed in 9/13 cases of AME graft-elicited secondary brain development. (a')(b') Trajectories of SN-EGFP-labeled cells over 60 min around the H and B axes. At st. 7, H axis-directed cell movements in the primary brain (x, 58 ± 15 μm/h) and H axis-directed cell movements in the forming secondary brain (xi, 59 ± 19 μm/h) were both extensive. (xii) The tissue reorganization in the secondary brain continued to st. 8 (46 ± 13 μm/h).

Figure S12

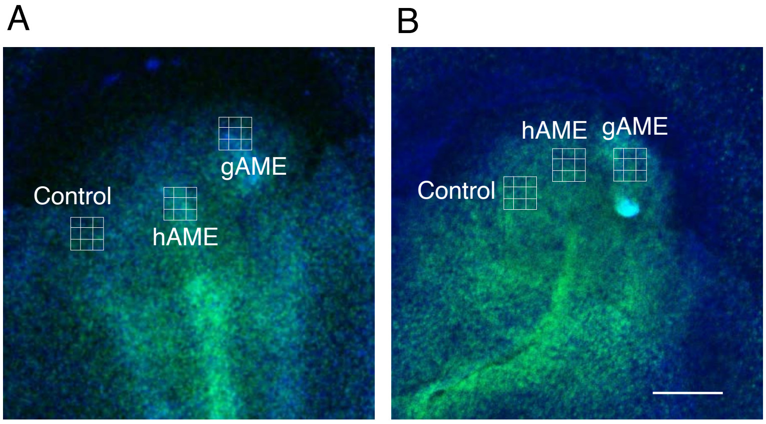

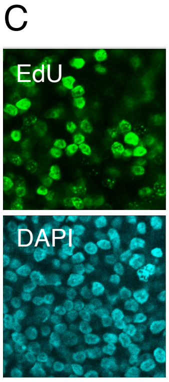

D

	EdU/DAPI (%)	
6-8 hr	Average	SD
gAME	55.4	±5.1
hAME	56.8	±4.1
Control	54.9	±8.0
8-10 hr		
gAME	49.4	±3.8
hAME	51.0	±4.9
Control	48.9	±7.4

Fig. S12. Detection of S-phase nuclei by EdU labeling of node-grafted epiblasts. (A) The epiblast of an embryo labeled with EdU after 6 to 8 h after grafting the node with DAPI nuclear staining. (B) An embryo labeled 8 to 10 h after grafting the node. After the EdU labeling, embryos were fixed and processed for Alexa 488 and DAPI fluorescence. Three rectangular regions of 234 × 234 μm^2 covering the node graft-derived AME (gAME), host AME (hAME), and on the contralateral side of gAME (AME-unaffected control) were chosen for analysis in each embryo. Each region was divided into nine subregions to determine the fraction of EdU-labeled nuclei in all DAPI-stained nuclei. Bar, 500 μm. An example of such a subregion comparing EdU labeling and DAPI staining is shown in (C). An average of nine subregions in each measurement region is shown in (D) with standard deviations. An EdU labeling frequency of 56% was estimated for the embryo in (A) (6–8 h labeling) and 50% for (B) (8–10 h labeling).

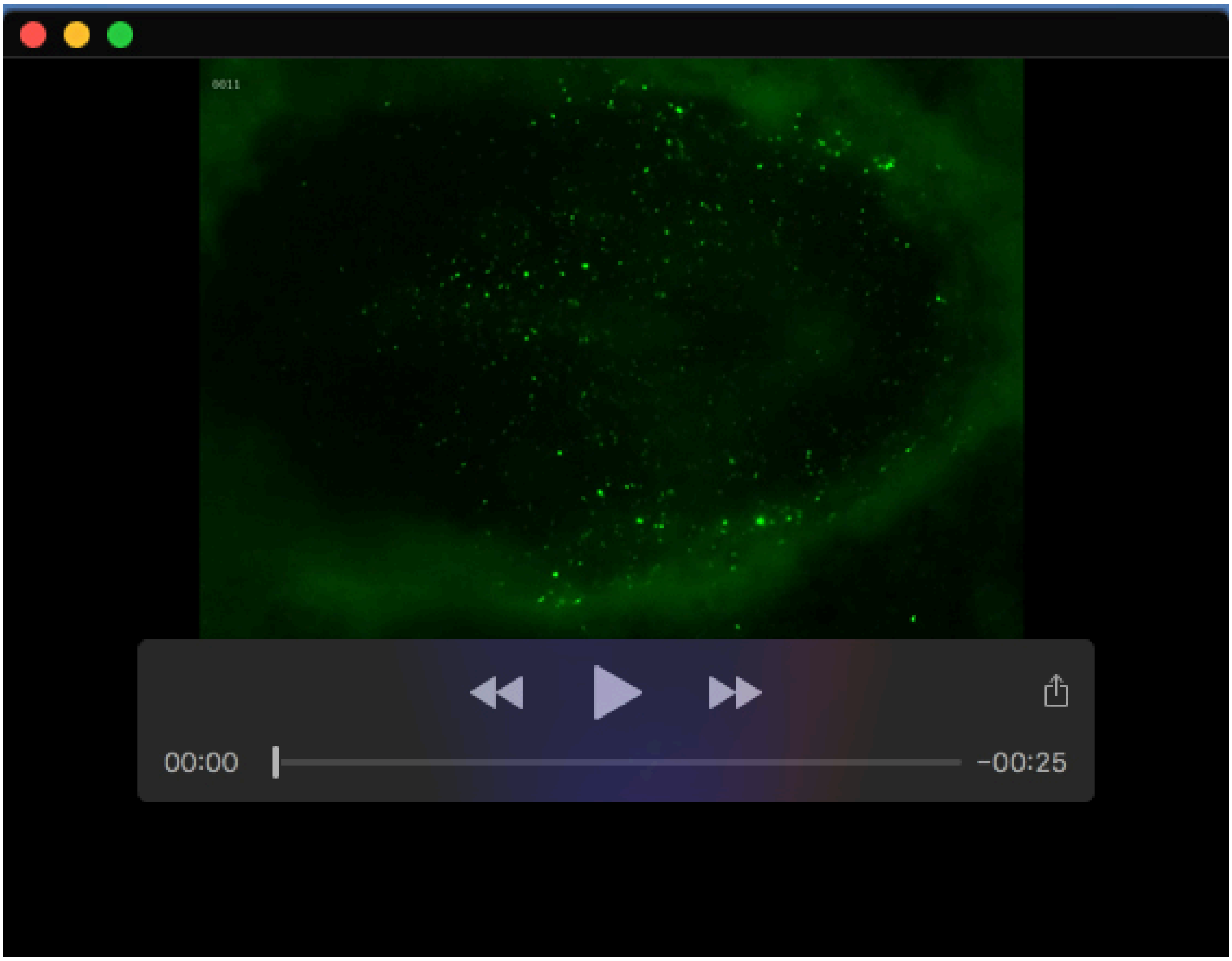

Movie 1. A representative time-lapse recording of SN-EGFP-labeled epiblast cells from st. 5 to st. 9, showing the convergence of the epiblast cells toward the midline even in long distances. The frames were taken at 10 min intervals and converted to the movie at 6 fps. The original data for Figure 1(A)(a).

Development • Supplementary information

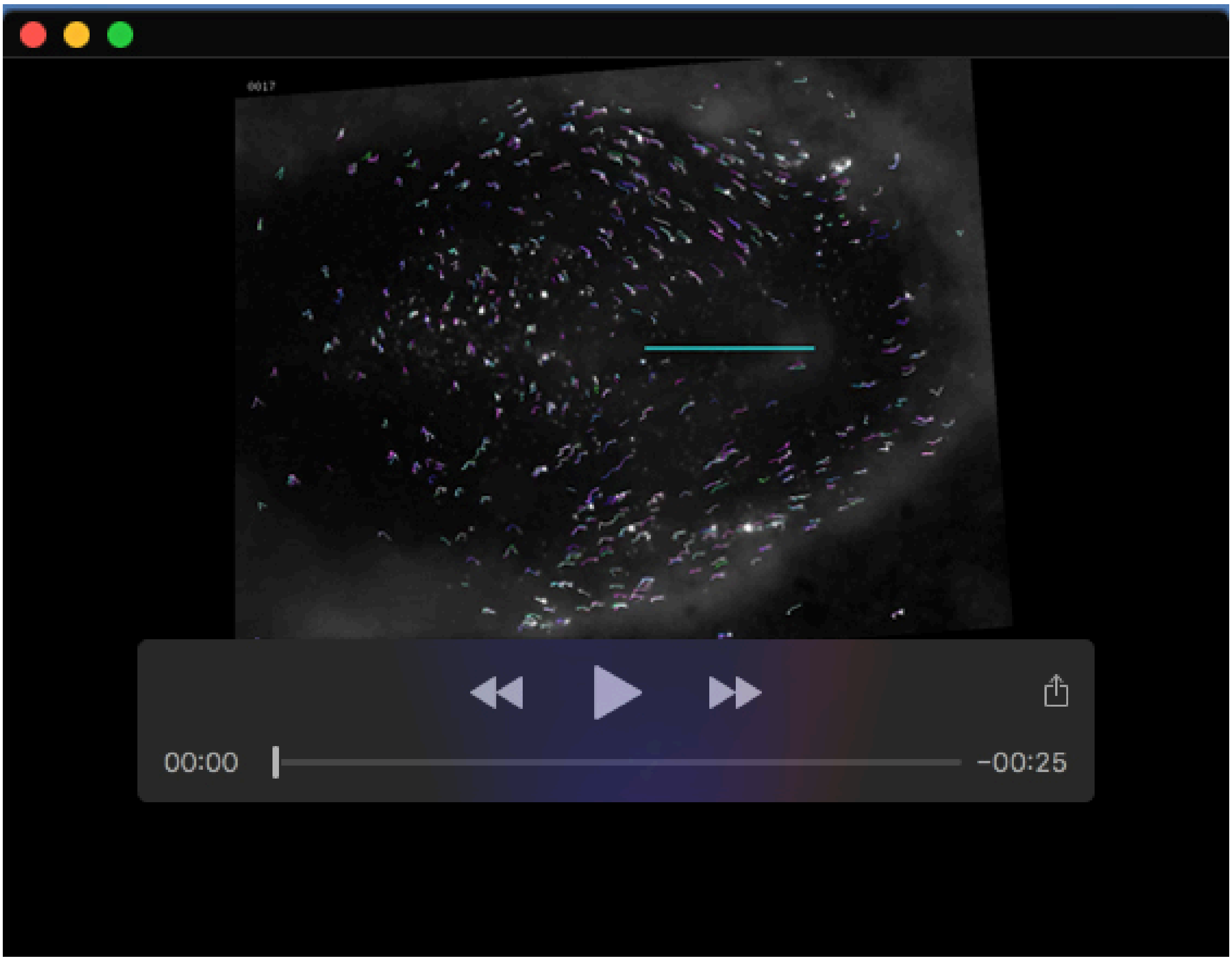

Movie 2. Trajectories of SN-EGFP-labeled cells relative to the head axis (magenta line) during st. 5 – 8 of the embryo shown in Movie 1. Trajectories covering more than 8 consecutive frames are shown. Original frames were at 10 min intervals and shown at 6 fps. The original data for Figure 1(A)(b).

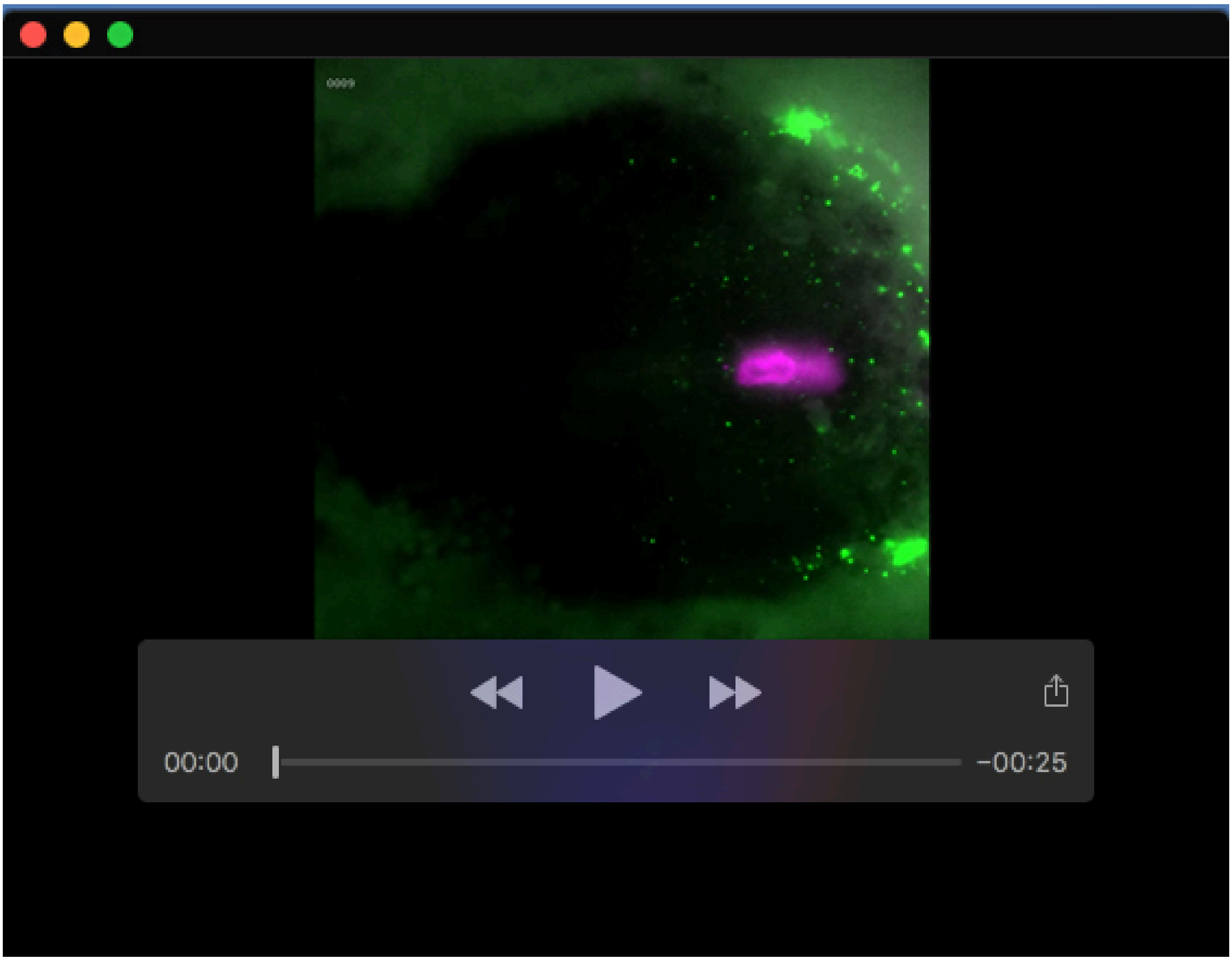

Movie 3. A representative time-lapse recording of an mCherry-labeled st. 4 Japanese quail node orthotopically grafted in a SN-EGFP-labeled embryo from st. 5 to st. 8. This movie shows the development of a st. 4 node into the anterior mesendoderm (AME) and posterior notochord (PNC) and the convergence of the anterior epiblast cells toward the midline. The frames were at 10 min intervals and shown at 6 fps. The original data for Figure 1(B)(a).

Development • Supplementary information

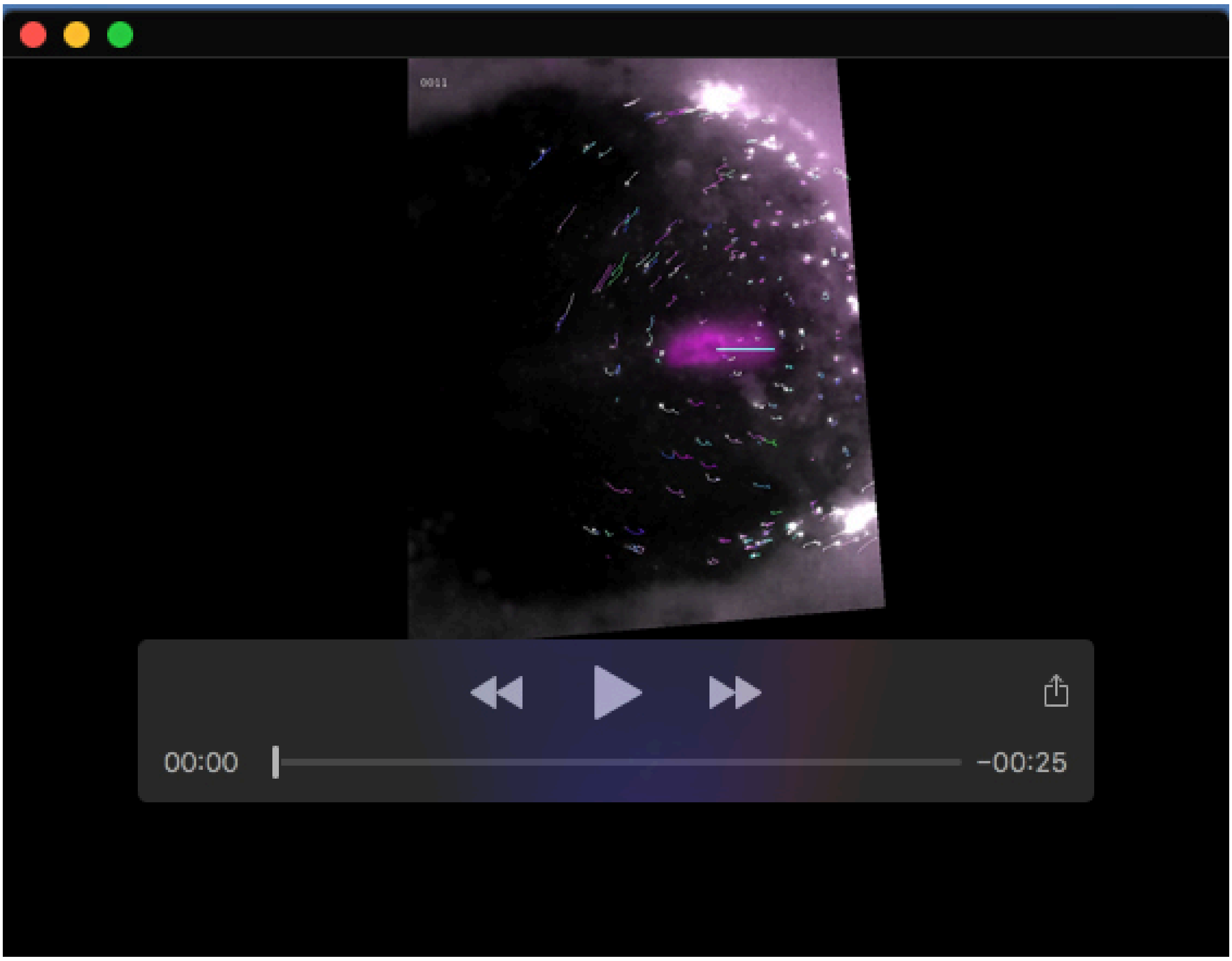

Movie 4. Trajectories of SN-EGFP-labeled cells relative to the axis of the node-derived AME (magenta line) during st. 5 – 8 of the embryo shown in Movie 3. Trajectories covering more than 8 consecutive frames are shown. This movie confirms the epiblast convergence toward the AME position (magenta line). Original frames were at 10 min intervals and shown at 6 fps. The original data for Figure 1(B)(b).

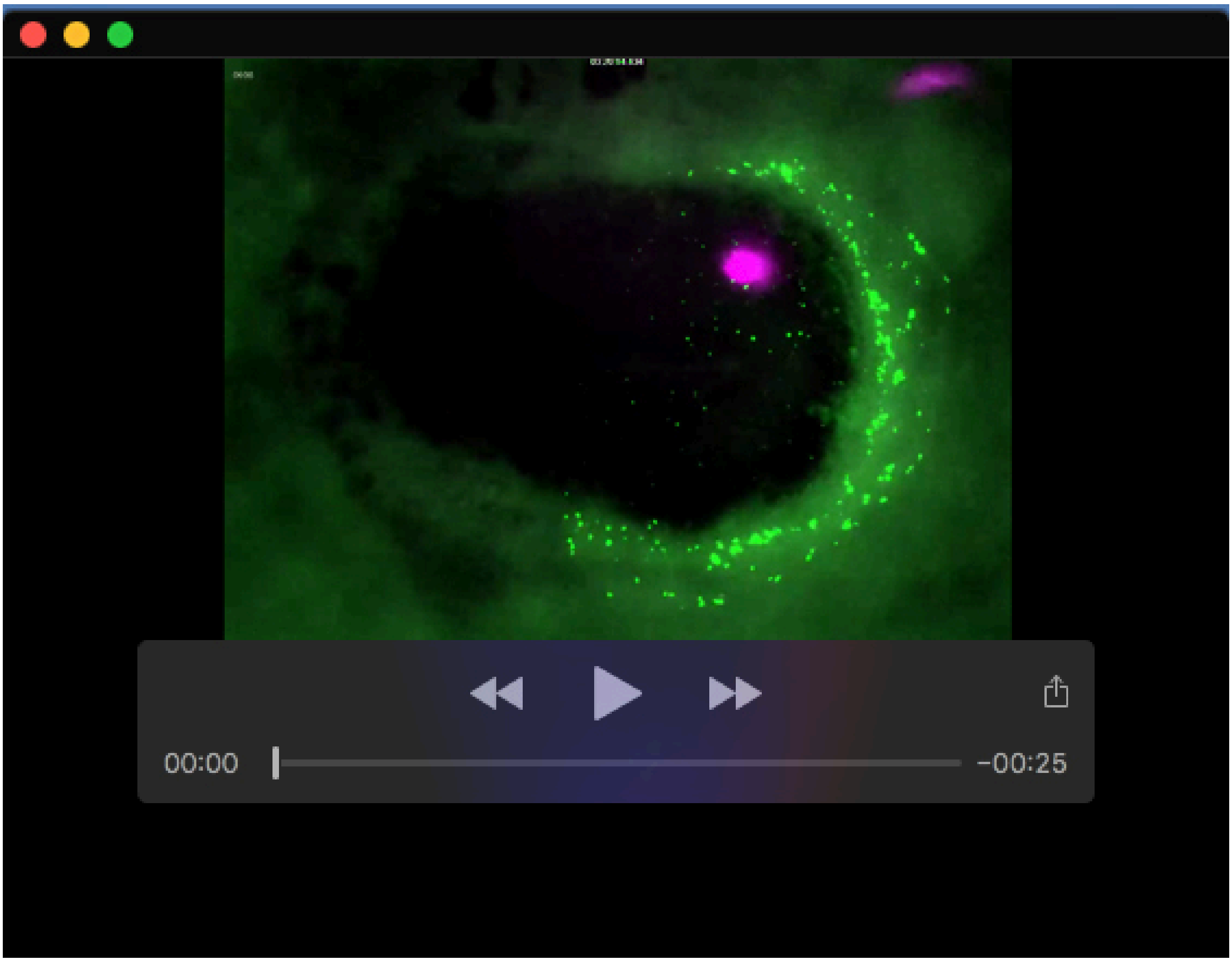

Movie 5. A representative time-lapse recording of a SN-EGFP-labeled epiblast from st. 4 to st. 9 with a graft of mCherry-labeled st. 4 Japanese quail node at an anterolateral position of the host node. This movie shows the node graft development into an ectopic graft-derived AME (gAME) and the convergence of the surrounding epiblast cells toward the gAME, which elicited the secondary brain tissue development, followed by its fusion to the host brain. The frames were at 25 min intervals and shown at 6 fps. The original data for Figure 5(A)(a') to (d').

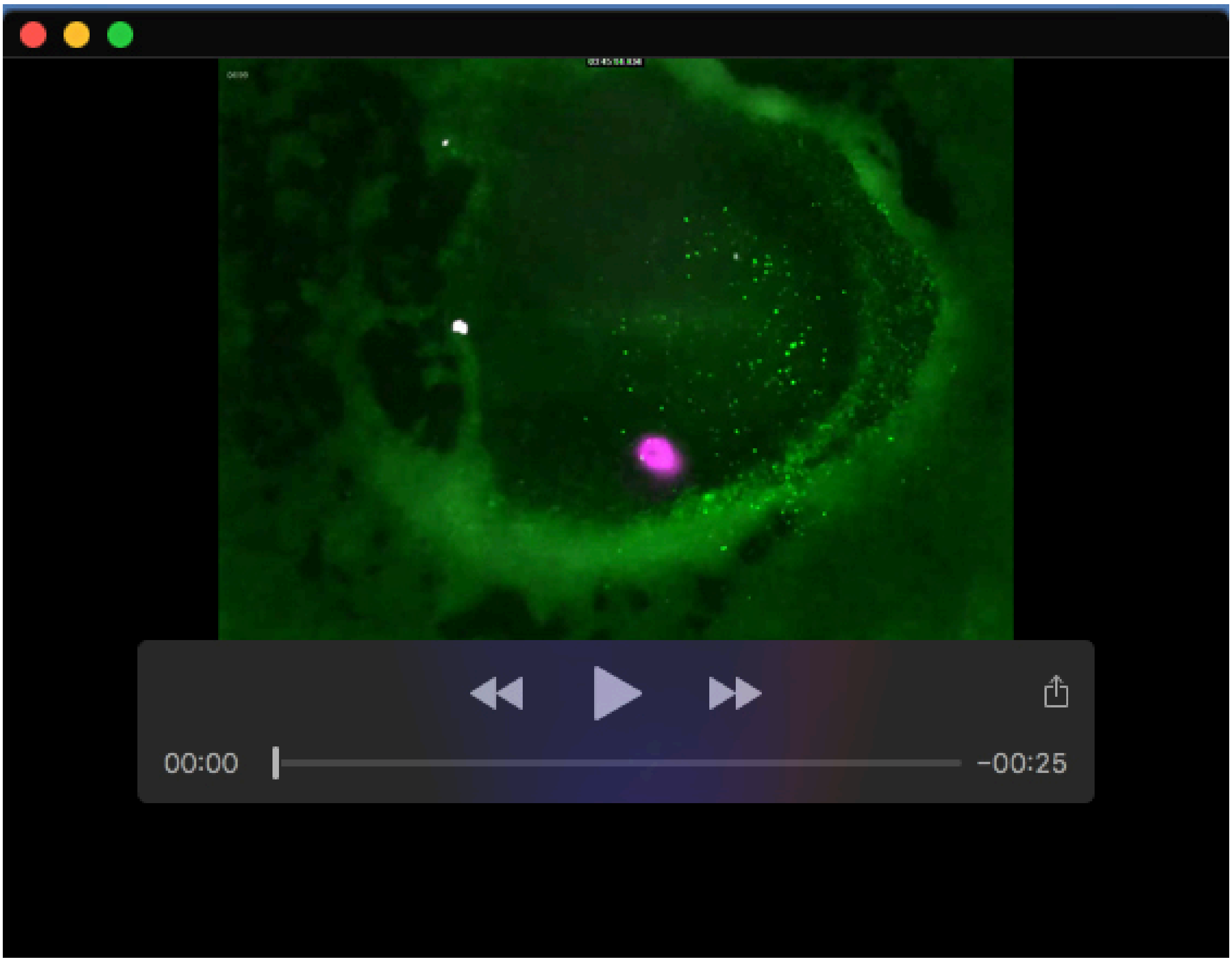

Movie 6. The second example of ectopic mCherry-labeled node graft in SN-EGFP-labeled epiblast field analogous to Movie 5, but the node graft position lateral to the host node. The original fluorescence data for Figure 5(B).

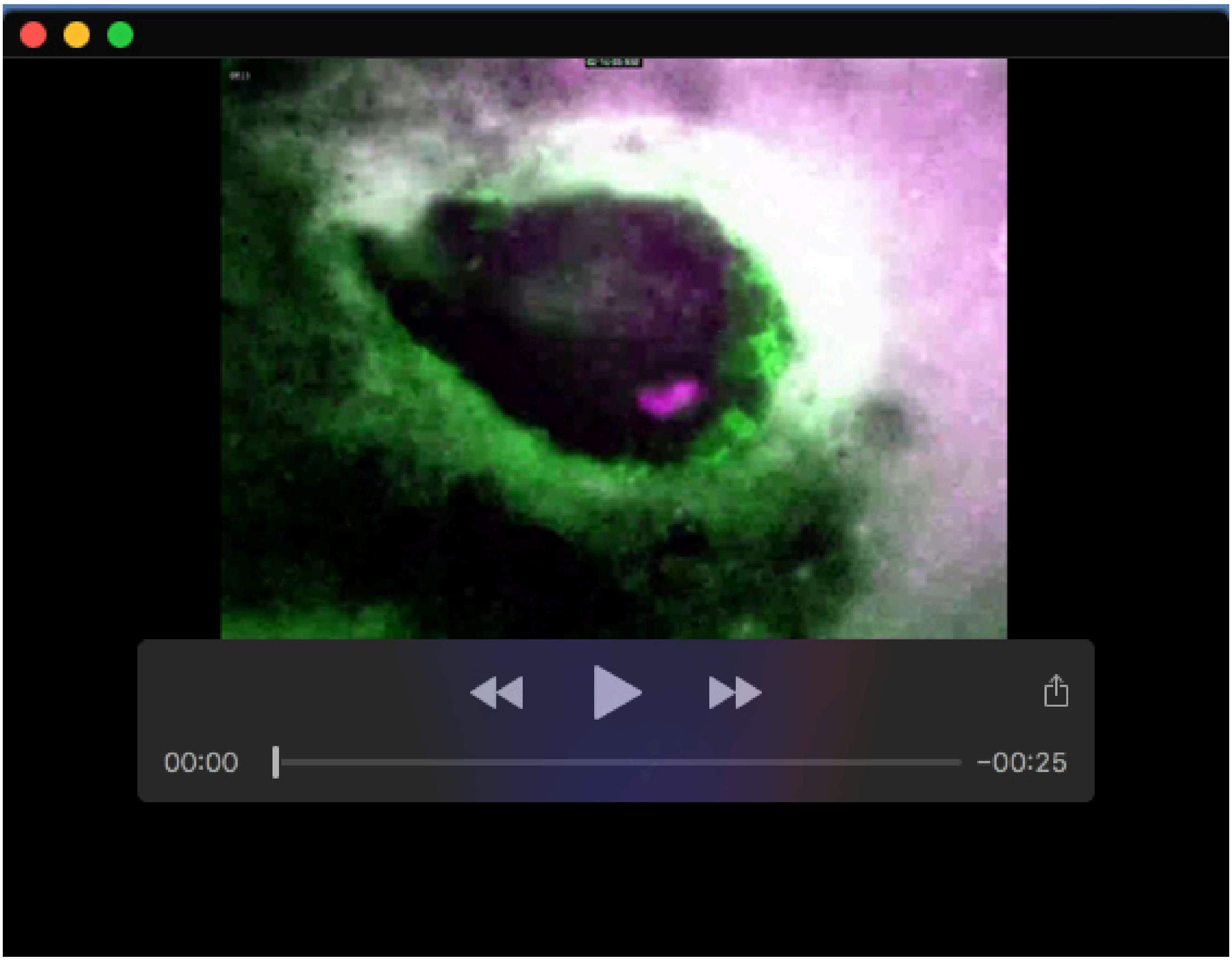

Movie 7. A representative time-lapse recording of a SN-EGFP-labeled epiblast from st. 4 to st. 9 with a graft of mCherry-labeled st. 5 Japanese quail AME at an anterolateral position of the host node. The AME graft developed further into the anteriormost prechordal plate (PP) and more posterior, anterior notochord (ANC). The surrounding epiblast cells converged toward the gAME tissues similarly to following a node graft, formed the secondary brain tissue, and eventually fused to the host brain. The frames were at 10 min intervals and shown at 6 fps. The original fluorescence data for Figure 5(C).

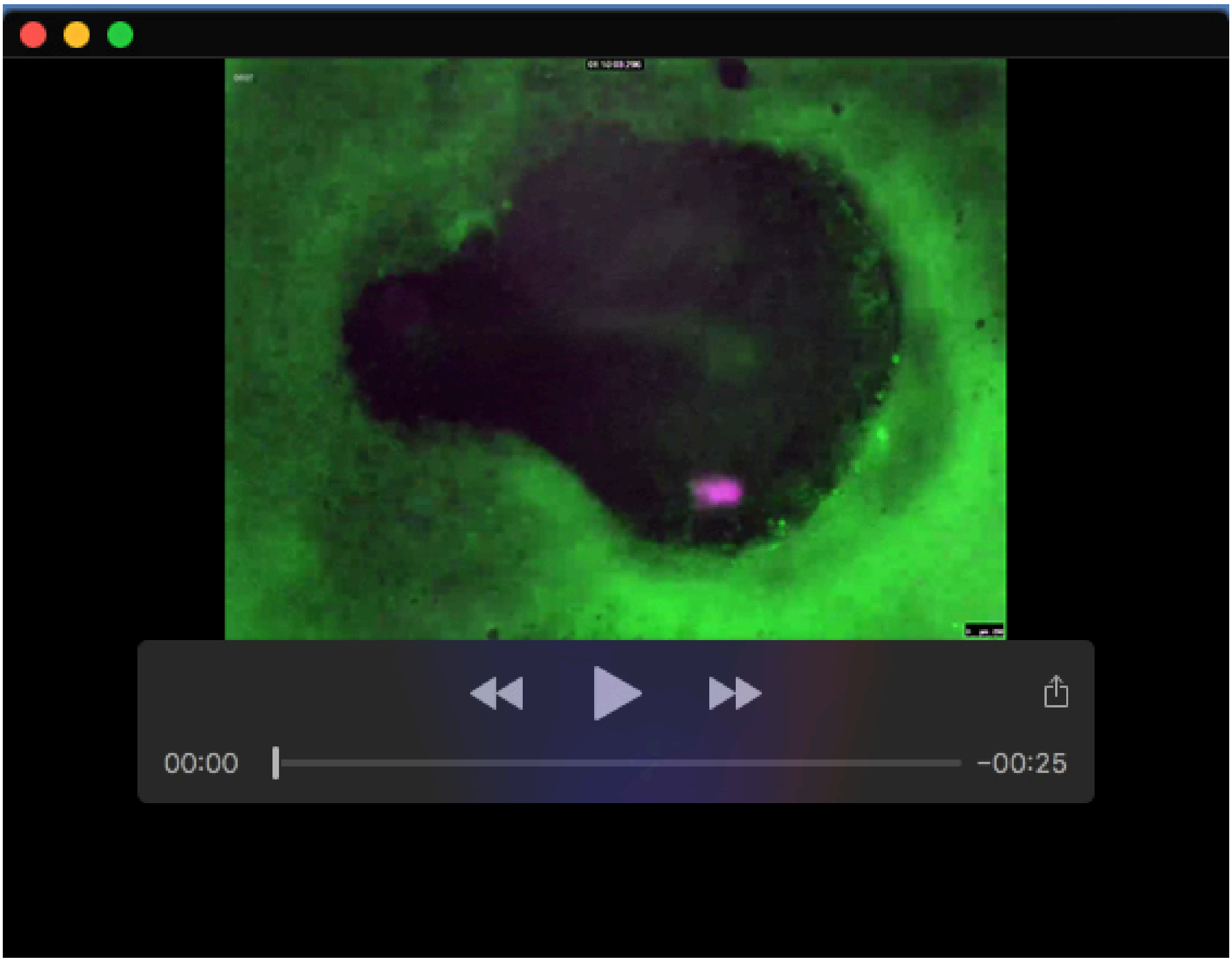

Movie 8. The second example of ectopic mCherry-labeled AME graft in SN-EGFP-labeled epiblast field analogous to Movie 7, but the node graft position more remote and lateral to the host node. The secondary brain tissue that developed surrounding the gAME was fused to the host brain only at its posterior end due to head ectoderm development separating the two brains at the anterior positions. Note also the contribution of area opaca-derived cells in the secondary brain. The original fluorescence data for Figure 5(D).
