# Supplementary material for: Live imaging of avian epiblast and anterior mesendoderm grafting reveals the complexity of cell dynamics during early brain development
Source: Development. 2022 Mar 28;149(6):dev199999. doi: 10.1242/dev.199999 (PMC9017232; doi:10.1242/dev.199999)
Supplement: Reviewer comments [file develop-149-199999_review_history.pdf]

Live imaging of avian epiblast and anterior mesendoderm grafting reveals the complexity of cell dynamics during early brain development

Koya Yoshihi, Kagayaki Kato, Hideaki Iida, Machiko Teramoto, Akihito Kawamura, Yusaku Watanabe, Mitsuo Nunome, Mikiharu Nakano, Yoichi Matsuda, Yuki Sato, Hidenobu Mizuno, Takuji Iwasato, Yasuo Ishii and Hisato Kondoh
DOI: 10.1242/dev.199999

Editor: James Briscoe

Review timeline

Original Submission:	21 April 2021
Editorial decision:	19 May 2021
Resubmission:	12 July 2021
Editorial decision:	16 August 2021
First revision received:	27 September 2021
Editorial decision:	21 October 2021
Second revision received:	22 November 2021
Editorial decision:	5 January 2022
Third revision received:	13 January 2022
Accepted:	18 January 2022

Original submission decision letter

MS ID#: DEVELOP/2021/199722
MS title: Live imaging of avian epiblasts revealing a new precursor map and a role for anterior mesendoderm in brain development

Authors: Koya Yoshihi, Kagayaki Kato, Hideaki Iida, Machiko Teramoto, Akihito Kawamura, Yusaku Watanabe, Mitsuo Nunome, Mikiharu Nakano, Yoichi Matsuda, Yuki Sato, Hidenobu Mizuno, Takuji Iwasato, Yasuo Ishii, and Hisato Kondoh
Article type: Research Article

Dear Dr. Kondoh,

I have now received all the referees reports on the above manuscript, and have reached a decision. The referees' comments are appended below, or you can access them online: please go to BenchPress and click on the 'Manuscripts with Decisions' queue in the Author Area.

As you will see from their reports, the referees recognise the potential of your work, but they also raise significant concerns about it. All three referees acknowledge the quality of the time-lapse studies but each had difficulties following aspects of the study and requested additional analysis and quantification. Overall, the referees' opinion is that the work does not provide sufficient new understanding to the field. I agree with this view. Given the nature of these concerns, I am afraid I have little choice other than to reject the paper at this stage.

However, having evaluated the paper, I do recognise the potential importance of this work. In particular, developing the analysis of the cellular movements responsible for the formation of anterior regions of the neural tube, and of the different patterns of cell movements anterior and posterior to Hensen's node would greatly strengthen the study. I would therefore be prepared to

© 2022. Published by The Company of Biologists under the terms of the Creative Commons Attribution License (https://creativecommons.org/licenses/by/4.0/).

consider as a new submission an extension of this study that contains new experiments, data and discussions and that address fully the major concerns of the referees. The work required goes beyond a standard revision of the paper. Please bear in mind that the referees (who may be different from the present reviewers) will assess the novelty of your work in the context of all previous publications, including those published between now and the time of resubmission.

If you decide to resubmit, please go to BenchPress and click on the 'Submit a new manuscript' link within the Author Area.

Please ensure that you click the 'This is a resubmission' checkbox, and enter the manuscript identification number shown above. I would also ask you to provide in the cover letter an explanation of the key ways in which the manuscript differs from the current submission, followed by a point-by-point response to the referees' concerns.

Reviewer 1

Advance summary and potential significance to field

In this paper, Yoshihi et al present some very beautiful work documenting the response to grafts of the node and emerging "AME" into various positions in the area pellucida of the developing chicken embryo. The ability to track individual epiblast cells and so monitor patterns of contribution to the neural axis over time in normal and grafted embryos provides insight into cell movements underlying formation of the head region. However, despite the authors claims here, the data presented seem unsurprising: the main finding that nodes or "AME" induce different regions of the CNS depending on graft position in the st4 anterior epiblast is expected and consistent with the epiblast possessing some regional character at this stage. It is also not clear to this reviewer how findings here disagree with the concept of an organiser, which of course changes its nature (cell populations and signalling abilities) as development proceeds. For these reasons I think the paper in its current form is potentially confusing for the field.

Comments for the author

Other hopefully helpful comments:

i) The Abstract lists experimental findings but does not derive a conclusion.

ii) Page 3 " This study focused on stage (st.) 4 chicken embryos (Hamburger and Hamilton, 1951), a stage when the node, formed at the anterior end of the primitive streak, develops only to the anterior mesendoderm (AME) and the posterior notochord (PNC), as shown in this study" How do the authors explain the detailed fate map of the st4 node generated by single cell labelling which shows that this also contains cells fated to form medial somites ? (Selleck and Stern 1991) https://pubmed.ncbi.nlm.nih.gov/1794328/

iii) Page 6 paragraph 1 - the authors describe cell movements observed as displaying "collective cell migration" - this term is usually used to describe movement of cells as a group I am not sure there is evidence for this here - rather the authors are tracking the trajectories of individual cells.

iv) Page 8 paragraph 1 - The fate mapping results for the neural plate are indeed consistent with Fernández-Garre et al. (2002) and of Schoenwolf & Alvarez (1991); the findings presented here seem confirmatory.

v) Page 9 "we hypothesized that the anterior epiblast cells outside the brain precursor region have a hidden potential of brain development, which is not manifested in normal development." Surely, this is already known from the many grafts placed in the area pellucida from Waddington onwards. Indeed, a "border" region around the neural plate has been characterised by others and it is known that the neural/epidermal border is regulated by signalling provided by the node/derivatives https://pubmed.ncbi.nlm.nih.gov/9834186/

© 2022. Published by The Company of Biologists under the terms of the Creative Commons Attribution License (https://creativecommons.org/licenses/by/4.0/).

vi) The neural inducing and regionalising properties of the "AME" or prechordal mesoderm have been analysed previously https://pubmed.ncbi.nlm.nih.gov/9247340/

vii) It is not clear to this reviewer on what basis the potential to generate hindbrain and midbrain resides anterior to that of the forebrain in Figure 7 - this could be further explained here, is it simply that grafts are orientated towards the host forebrain ?

viii) In the Discussion, p18 " Many previous node graft experiments selected the proximal regions of the area opaca (Storey et al., 1992) or germinal crescent at the epiblast anterior end (Dias and Schoenwolf, 1990) as grafting sites. These experimental maneuvers assumed that these regions are not specified and hence bear no embryo regionalities".

Storey et al 1992 refer to the area opaca as "more neutral" than the area pellucida, it is not assumed to be a completely neutral environment. The assumption in this study was that AO epiblast cells were not committed to making neural tissue but had the potential to do this if exposed to correct signals. The regional character of the induced neural tissue in these cells altered depending on age of node and also stage of host - at an earlier stage a more complete regionalised neural axis could be induced consistent with gradual regionalisation of the epiblast. While the different inducing abilities of different staged nodes reflected the changing cell populations in the node.

Reviewer 2

Advance summary and potential significance to field

The manuscript entitled "Live imaging of avian epiblasts revealing a new precursor map and a role for anterior mesendoderm in brain development" is describing the chick gastrula epiblast cell tracing results obtained by the authors using the Supernova sparse labelling technique. The authors provide a high-quality map of the cell movements from stage 4 to 9. They then characterise cell movements after stage 4 node and stage 5 anterior mesendoderm (AME) grafting in the non-neural epiblast. Very preliminary analysis of these grafts suggests, mostly based on graft morphology, that non-neural epiblast has a A/P restricted competence at the time of the graft. Overall, the manuscript shows a series of nice time-lapse studies but does not provide enough new understanding to the field. The data systematically lacks quantification and the text is difficult to follow in many places. Lack of quantification (even no mention of number of embryos analysed) and specific AP markers substantially weaken the authors' conclusions.

Comments for the author

Specific comments:
- The cell tracking reproduces the movements already published by Weijer and others. Furthermore, claims of collective migration are not supported by any quantification of cell movements.
- The authors down-play the difference between their neural plate fate mapping results and the ones published by the Puelles lab at the same stage. The difference is not only the position of the anterior neural plate border (a difference that is very big: 250 vs 500 microns!). The cells forming the forebrain in the Puelles fate-map are also located near the node forming a V shape field of cells. The authors do not find cells in front of the node participating to the forebrain area. In their case, the first cells anterior to the node are midbrain cells. This is a conceptually very meaningful difference that needs to be verified by further mapping and properly discussed. The Puelles data is weak on these medial cells so it is important to unambiguously determine the fate of these cells.
- The finding that head ectoderm cells transiently express sox2 is interesting and suggests indeed a potential for neural fate that is switched on if node or AME is grafted. However, the link between sox2+ ectoderm and competence to respond to node signals is only correlative.
- Grafting in the lateral epiblast (future head ectoderm) suggests that these are committed to their anterior-posterior position as early as stage 4/5. More precise AP markers would be needed to ascertain this finding.
- Grafts of anterior mesoderm rather than node reveal that anterior mesoderm alone is sufficient to induce neural tissues. This could provide some new understanding if the authors would use a series of markers identifying the set of AP identities induced by these cells.

© 2022. Published by The Company of Biologists under the terms of the Creative Commons Attribution License (https://creativecommons.org/licenses/by/4.0/).

Figures comments
- Figure 1: Legend needed to understand the colours of each line representing cell trajectories.
- Figure 4 should mention how many grafts were done for each of the a-f positions and present marker analysis for each position to ascertain AP identities of the graft-induced CNS
- Figure 5 is very difficult to understand and needs quantification
- Figure 6 has same issues as Figure 4

Reviewer 3

Advance summary and potential significance to field

This study addresses how the anterior neural tube/early brain arises in the chick embryo over the period HH5-HH9. A key strength of the study is that the authors perform real time image analysis of cell movements during neural plate formation/neurulation, after eGFP labelling. The authors show that in normal development, epiblast cells converge and extend anteriorly to form the neural tube. By 'back-tracing' cells from their final destination at HH9 to their origin at HH5, the author develop a map to show the position of forebrain, midbrain and hindbrain precursors, and of head ectoderm precursors at HH5. This leads them to suggest that epiblast cells have the potential to give rise to neural tube or head ectoderm, and that fate is determined by proximity to the axis. To identify a potential role for axial mesendoderm, the authors graft nodes (capable of giving rise to anterior mesendoderm (AME)) to ectopic positions and show that host-derived secondary neural tube-like structures form around the differentiating grafts. The authors suggest that the type of neural tissue formed is dependent on the A-P position of the graft (anterior > forebrain-like; mid > hindbrain-like; posterior > posterior neuraxis/floor plate?). Finally the authors perform a technically challenging experiment, combining grafting with live-imaging to try to argue that the AME directs surrounding head precursors to converge and develop into the brain.

Previous studies have generated fate-maps of the chick neural tube at HH4, have shown the ability of the Node and node-derived structures (including the AME) to promote ectopic neural tissue, and have suggested that epiblast cells can generate neural or ectoderm tissue. The state-of-the-art techniques (sparse labelling/live imaging) used in this study confirm previous fate-maps and ideas on epiblast cell potential, but are not necessarily novel findings. The key advance, therefore is to show that anterior regions of the neural tube develop through a mechanism that involves convergence towards the AME. A second key advance is the very different patterns of cell movements, anterior or posterior to Hensen's node. This observation is interesting, but not followed up in the work.

Comments for the author

Despite its novelty, the study has a number of limitations.

1. Fig 1, Movies 1-3: the data demonstrates, very nicely, that there is a general cell movement towards to midline and then anteriorly (the authors term this collective migration). But looking at individual cell tracks, it is hard to be certain that this is a convergence to the AME. First, most of the cells imaged end up in the dorsal aspect of the neural tube. Second, looking at the tracks of individual cells (Fig 1A panel (b); Fig 1B pane; (b) and Movie 2, they appear to converge towards the midline, then to track parallel to this. While I agree that cells are converging towards the general midline, this does not necessarily argue for a convergence of cells toward the axial AME. The collective migration could be driven through other means.

2. Fig 2: The authors trace back the trajectories of individual cells, to determine the position of forebrain, midbrain and hindbrain precursors at HH5 and HH6. This relies on having a clear way to define the hindbrain, midbrain and forebrain at HH9. The authors use Gbx2 and Otx2 to define the HB/MB boundary (note the Otx2 labelling cannot be detected in Fig 2A (a) - so this needs to be repeated). However, they then suggest that they 'copy these boundaries on the SN-EGFP image of the same embryo' (bottom line p6). This does not seem to be the case: comparison of Fig2A panels (a) and (b) does not show the MB/FB boundary at the same position. This makes it very difficult to judge the FB/MB data shown in the rest of this figure (panels B and C).

© 2022. Published by The Company of Biologists under the terms of the Creative Commons Attribution License (https://creativecommons.org/licenses/by/4.0/).

3. Fig 3: This figure (text and fig legend) is hard to follow. In particular it is not clear how the final position of each 'back-tracked' cell was determined. The authors need to explain this and justify how they decided if a cell was in the neural tube, the dorsal neural tube or the proximal head ectoderm. This justification needs to be applied to Fig 2 as well. To explain this point by point: -If I understand correctly, panel (A) shows the position of precursors that will end up in the neural tube. Where has the data come from? It is not the same as that shown in Fig 2B panel (b)?
-Panel B shows the same embryo: this time the dots show the position of precursor cells that will end up in the 'dorsal brain/proximal head ectoderm' Again - where has the data come from?
-The authors need to show an example, similar to those shown in Fig 2 to show that they can distinctly trace back cells that end up in the neural tube versus the dorsal neural tube/head ectoderm. Without having such higher resolution data I am struggling to understand the data in panels A and B, and so struggling to understand how Fig 3 adds to previous studies.

4. Fig 4: Please provide examples of real image of acute grafts - to show the position and size of the graft relative to the 'brain map' at HH4-5 (ie relative to the dots shown in Fig 3). The schematic shown in Fig 4A is not sufficient.

5. (see also point 9) Fig 4: The authors need to use markers to confirm the composition of cells that differentiate from the Node grafts - ie markers that confirm that AME and not notochord, differentiate when grafts are positioned anteriorly, and that notochord, and not AME, differentiate when grafts are positioned posteriorly (Fig 4Ba-f).

6. (see also point 9) Fig 4: Similarly, markers that distinguish between forebrain, midbrain, hindbrain and spinal cord need to be used to confirm the types of brain region that develop ectopically

7. Fig 4 panel C: The idea that secondary brain development occurs at the expense of primary brain is really interesting, but raises many questions. Are the authors suggesting that there is a limited supply of cells with the potential to give rise to the brain? How does this fit with the idea that epiblast cells have potential to give rise to brain? This is a key point - ie the origin of the cells that generate secondary structures - and ties into the questions raised regarding figs 2 and 3.

8. Fig 4 panel C: Sections are needed to show the extent of the missing dorsal tissue - it is not possible to see this in the wholemount view.

9. Fig 6: Assertions as to development of particular brain regions are based partly on morphology and partly on expression of Otx2 and Gbx2. The Gbx2 labelling is informative, but expression of Otx2 cannot be detected. The authors need to show improved in situs, and add probes that distinguish between FB and MB.

10. The experiment to combine eGFP labelling, with grafts and real time imaging is technically extremely challenging, and the authors should be congratulated on achieving this. Frustratingly, though, it was still difficult to work out cell movements. The eGFP labelling is weak at a time when key events could be occurring and the image resolution was quite low. The authors conclude that precursors close to the graft start to converge on it, whereas those 'in the remaining region' (I am not sure what this refers to?) converge on the host AME. But this was difficult to see. Largely, it was impossible to see how the cell movements after grafting related to those in the normal embryo. This makes it difficult to tie together the in vivo observations and the grafting studies. This significantly detracts from the overall impact of the paper.

11. Likewise, it is difficult to interpret the cell movements in Fig 5B. It is difficult to know whether this is a convergence of cells towards the grafted tissue.

12. Fig5C and D: in these instances, where HH5 AME tissue has been grafted (instead of HH nodes that can differentiate to AME) it is difficult to make out the type of host tissue that develops ectopically - these are labelled as FB/MB/HB - but independent markers are needed to confirm this.

© 2022. Published by The Company of Biologists under the terms of the Creative Commons Attribution License (https://creativecommons.org/licenses/by/4.0/).

Minor comments

1.It would be helpful to the reader to better define the AME: ie the region of mesoderm and endoderm that has extended from the Node by HH5 and that will differentiate into prechordal mesendoderm, prechordal mesoderm and head process notochord.

2. The Introduction needs some changes as it begins to introduce results: these should go at beginning of results section

3. Fig 3A legend: please clarify that dots refer to data generated here and show the position at HH5, whereas shaded regions refer to a previous fate-mapping study (Fernandez-Garre) and show the position at HH4.

4. P7: 'where relative position was … conserved' from st 6 (not in st 6)

5. Fig 4 panel C: please switch orientation so that it is obvious that this is an enlargement of Fig 4B panel (b).

Response to reviewers' comments

We appreciate the constructive criticisms of the editor and reviewers on our previous manuscript. We have revised the manuscript on the following new framework while responding to the points of the reviewers.

The live imaging-based analyses in this study targeted the following two processes: the wide-range convergence of the anterior epiblast to the head axis in the brain-forming period and the ectopic anterior mesendoderm (AME)-directed local epiblast convergence leading to secondary brain development. In the new version of the manuscript, we have explicitly expressed the novelties of our findings, adding new critical sets of data (Figs. 2, S3-S5, and 8).

The significant improvements in the manuscript contents are itemized below.

The new Introduction section summarizes the novel findings of this study compared to earlier studies to orient the readers:

1. In the past, epiblast cell migration during the primitive streak only before st. 4 was investigated; the analysis of epiblast cell migrations in the head-forming stage after st. 4 in this study is brand-new.
2. The Supernova-based epiblast cell labeling (SN-EGFP) and cell tracking analysis revised the Fernández-Garre brain portion precursor map.
3. Live imaging of the node-derived tissue development after grafting allowed new observations. It distinguished AME development and posterior notochord (PNC) development in the direction and timing of tissue extension.
4. The simultaneous live imaging of the epiblast and node/AME grafts revealed new cell gathering activity of the AME, leading to secondary brain tissue development.
5. This paper proposes a brain portion potential map of the anterior avian embryo field, integrating our new findings and previous observations on node grafts at or outside the area pellucida periphery.

The Results section was revised, adding new data and improved data presentations.

1. In addition to the extended tracking of SN-EGFP-labeled cells (Fig. 1), the time- resolved short track data are presented in the new Fig. 2 and Fig. S3.
2. The procedures in the backtracking of the st. 8/9 brain portion boundaries to st. 5/6 epiblast are explained concisely in the Results section and detailed in a new chapter in the Materials and Methods section.
3. In the previous version of the manuscript, four embryo data were superimposed to draw the head portion precursor maps [current Fig. 4(A)]. In the new version, the new Fig. S4 is provided, showing embryo-to-embryo variations in the brain portion precursor

© 2022. Published by The Company of Biologists under the terms of the Creative Commons Attribution License (https://creativecommons.org/licenses/by/4.0/).

distributions. This data provided a ground for revising the Fernández-Garre brain portion precursor maps.

4. The new Fig. S6 presents the live imaging-based analysis of the graft position- dependent development of node grafts. This analysis demonstrated that the node grafts in the anterior embryo region develop only into the AME tissues, whereas that in the posterior embryo region only caused the PNC development. These tissues were distinguished not only by the tissue morphology but also by the timing and direction of tissue extension.
5. In Fig. S6 and on all possible occasions, the immediate snapshots of the node/AME grafts in st. 4 host embryos are presented.
6. In the new Fig. 5 (previous Fig. 4), data were rearranged with an additional histological section.
7. The new epiblast schemes included the brain portion precursor map (Figs. 5 and S7). The numbers of embryos used in each specimen group are stated.
8. The new Fig. 6 (previous Fig. 5) panels avoiding superimposition with the bright- field images and using brighter images clearly visualize the SN-EGFP-labeled epiblast cells and their convergence on graft-derived AMEs. The corresponding new movies (Mov. 5-8) vividly illustrate the cell convergence events. The in situ hybridization data were also included in this Figure.
9. The new Fig. 7 (previous Fig. 6) correlating the AME graft AP positions and the brain portions was remodeled using higher-quality in situ hybridization data with higher magnifications and a better summary scheme.
10. The new Fig. 8 (previous Fig. 7) to integrate the data of this study and those of earlier studies was extensively revised. The newly added data demonstrated that the far anterior position of the anterior epiblast has the hindbrain developmental potential. Accordingly, this Figure was moved from the Discussion section to the Results section.

The Discussion section was entirely rewritten and provided an in-depth analysis of the contents in the Results section.

Responses to the Reviewers
The itemized responses to the reviewers' points are given below in upright fonts.

Reviewer 1 Advance Summary and Potential Significance to Field...
In this paper, Yoshihi et al present some very beautiful work documenting the response to grafts of the node and emerging "AME" into various positions in thearea pellucida of the developing chicken embryo. The ability to track individual epiblast cells and so monitor patterns of contribution to the neural axis over time in normal and grafted embryos provides insight into cell movements underlyingformation of the head region.

However, despite the authors claims here, the data presented seem unsurprising: the main finding that nodes or "AME" induce different regions of the CNS depending on graft position in the st4 anterior epiblast is expected and consistent with the epiblast possessing some regional character at this stage.

This comment was addressed in response to the last point of this reviewer.

It is also not clear to this reviewer how findings here disagree with the concept of an organiser, which of course changes its nature (cell populationsand signalling abilities) as development proceeds. For these reasons I think the paper in its current form is potentially confusing for the field.

In the new version of the manuscript, the focus was placed more on the merit of live imaging. Besides, an effort was made to integrate the current and most previous observations using node graft experiments in a unified model, providing new data (Fig.8).

Reviewer 1 Comments for the Author...
Other hopefully helpful comments:

i) The Abstract lists experimental findings but does not derive a conclusion.

© 2022. Published by The Company of Biologists under the terms of the Creative Commons Attribution License (https://creativecommons.org/licenses/by/4.0/).

The Abstract was rewritten entirely.

ii) *Page 3 "This study focused on stage (st.) 4 chicken embryos (Hamburger and Hamilton, 1951), a stage when the node, formed at the anterior end of the primitive streak, develops only to the anterior mesendoderm (AME) and the posterior notochord (PNC), as shown in this study" How do the authors explain thedetailed fate map of the st4 node generated by single cell labelling which shows that this also contains cells fated to form medial somites ? (Selleckand Stern 1991)*

As shown in Fig. S2 and Fig. S7, the derivation of medial somites from the node tissue is the hallmark of st. 5 embryos. Selleck and Stern (1991) referred to by the reviewer indicated that some of their st. 4 specimens were actually at early st. 5.

iii) *Page 6 paragraph 1 - the authors describe cell movements observed asdisplaying "collective cell migration" – this term is usually used to describemovement of cells as a group I am not sure there is evidence for this here – rather the authors are tracking the trajectories of individual cells.*

The argument of collective cell migration stands on the observation that cells in an anterior epiblast area migrate coherently (in the same direction with the same velocity, maintaining their spatial relationships). This point was detailed in the new time-resolved analysis of the cell trajectories shown in the new Fig. 2.

iv) *Page 8 paragraph 1 - The fate mapping results for the neural plate are indeed consistent with Fernández-Garre et al. (2002) and of Schoenwolf & Alvarez(1991); the findings presented here seem confirmatory.*

In the previous version of the manuscript, we emphasized the overall consistency of the data between Fernández-Garre et al. (2003) and ours. However, our data revised the Fernández-Garre et al. map concerning details, as indicated in the new version of the manuscript.

The approach taken by Fernández-Garre et al. was to homotopically graft labeled epiblast disks of ~125 μm diameter to the endoderm-ablated regions of host embryos and to detect the positions of the labeled cells 24 h later. Although their analyses were detailed, we have identified some technical limitations. (1) In addition to the relatively large explant size, the orientation of individual explant was not known, limiting the spatial resolution of the map. In contrast, our analysis based on the live recording of many labeled cells scattered over the anterior epiblast field had a single-cell resolution. (2) The Fernández-Garre et al. map was drawn on the assumption that brain portion precursors are distributed identically among embryos, and the precursors for individual brain domains have defined boundaries. However, our analysis showed embryo-dependent variations concerning the brain portion precursor anterior limits. (3) The ablation of the endoderm layer in the region to plug in the donor epiblast disk could have affected the cell migrations when the operation was done along the midline.

Indeed, the Fernández-Garre et al. map showed the following abnormalities. (1) The presence of a dent in the anterior FB precursor boundary on the midline (the anterior limit on the midline was 250 μm from the node compared to 400 μm from the node level on the lateral sides) and (2) the absence of the MB precursors anterior to the node. Our data showed no such dent in the anterior limit of the FB precursor distribution. In addition, our data confirmed the presence of the MB precursors between the FB precursors and the node in all embryos examined.

As shown in Fig. 2 and Fig. S3, a fast-moving anterior cell stream occurs along the midline of the brain precursors, bringing the axial FB and MB precursors to their positions at st. 6, parallel to the AME extension from the node [Fig. 1(B), Fig. S6(A), Fig. 6(A)]. We speculated that the ablation of the endoderm layer in host embryos affected these two processes, resulting in abnormalities along the midline in the Fernández-Garre map.

In the new version of the manuscript, these points are discussed

v) *Page 9 "we hypothesized that the anterior epiblast cells outside the brain precursor region have a hidden potential of brain development, which is not manifested in normal development." Surely, this is already known from the manygrafts placed in the area pellucida from Waddington onwards. Indeed, a "border" region around the neural plate has been characterised by others and*

© 2022. Published by The Company of Biologists under the terms of the Creative Commons Attribution License (https://creativecommons.org/licenses/by/4.0/).

it is known that the neural/epidermal border is regulated by signalling provided by the node/derivatives.

In this paragraph, we should have emphasized the novelty of the systematic survey concerning the response of the possibly bipotential anterior epiblast field to grafts of st. 4 nodes or st. 5 AMEs, as done in the new manuscript. Waddington did not graft a node in isolation but as a part of the anterior primitive streak. In addition, precise donor embryo staging is essential. We commented on these points briefly.

vi) The neural inducing and regionalising properties of the "AME" orprechordal mesoderm have been analysed previously. https://pubmed.ncbi.nlm.nih.gov/9247340/

Foley et al. (1997) grafted the AME at two locations of the embryos: in the area opaca finding no significant effect and in a lateral pouch of the anterior germinal crescent located at the periphery of the area pellucida, observing the development of host-derived FB tissue. This latter effect is reinterpreted in our paper while integrating earlier observations with the current analyses (Fig. 8).

vii) It is not clear to this reviewer on what basis the potential to generate hindbrain and midbrain resides anterior to that of the forebrain in Figure 7 -this could be further explained here, is it simply that grafts are orientated towards the host forebrain?

New experimental data supporting and explaining the model are provided in the new version of the manuscript (Fig. 8). Data also confirm that the potential to generate HB and MB resides further anterior to that of the FB.

viii) In the Discussion, p18 "Many previous node graft experiments selected theproximal regions of the area opaca (Storey et al., 1992) or germinal crescent at the epiblast anterior end (Dias and Schoenwolf, 1990) as grafting sites. These experimental maneuvers assumed that these regions are not specified and hence bear no embryo regionalities".

Storey et al 1992 refer to the area opaca as "more neutral" than the area pellucida, it is not assumed to be a completely neutral environment. The assumption in this study was that AO epiblast cells were not committed to makingneural tissue but had the potential to do this if exposed to correct signals. The regional character of the induced neural tissue in these cells altered depending on age of node and also stage of host – at an earlier stage a more complete regionalised neural axis could be induced consistent with gradual regionalizationof the epiblast. While the different inducing abilities of different staged nodes reflected the changing cell populations in the node.

In the new version of the manuscript, we did not discuss the ground for choosing the area opaca as the site for the node graft. However, the observation of Foley et al. (1997) that grafting an AME piece in the area opaca did not elicit any host-derived neural tissue development left the essential activity of the AME tissues in the brain development unrecognized. Our study grafting the AME in the area pellucida demonstrated its activity.

The node is formed at st. 4, while its activity to elicit brain development at the ectopic grafted site is lost at st. 5. However, an activity analogous to the st. 4 node was found in the st. 5 AME. If an analysis were done only after 18 h of the tissue graft, in the way of classical experimental embryology, one might argue that the activity of the st. 4 node was passed over to the AME. However, the time-lapse recordings of fluorescence-labeled tissues indicated that not the grafted node per se but the AME that develops from the node after grafting is the entity that initiates the secondary brain development. This conclusion was confirmed by directly grafting AMEs. In addition, the AME grafting elicited neural tissue development at earlier stages of the host (~ 3 h) than after the node grafting, bypassing the AME elongation step starting from the node graft. Nevertheless, the same brain portions developed regardless of after the node graft or AME graft. This observation indicates that similar epiblast regionalization status is maintained between stages 5 to 6, arguing against the model of progressive specification of the epiblast during these stages. These conclusions were reached owing to the live imaging of the developmental process. We hope the readers recognize this as a part of the novelty of this study.

© 2022. Published by The Company of Biologists under the terms of the Creative Commons Attribution License (https://creativecommons.org/licenses/by/4.0/).

Reviewer 2 Advance Summary and Potential Significance to Field...
The manuscript entitled "Live imaging of avian epiblasts revealing a new precursor map and a role for anteriormesendoderm in brain development" is describing the chick gastrula epiblast cell tracing results obtained by the authors using the Supernova sparse labelling technique. The authors provide a high-quality map of the cell movements from stage 4 to 9. They then characterise cell movements after stage 4 node and stage 5 anterior mesendoderm (AME) grafting in the non-neural epiblast.
Very preliminary analysis of these grafts suggests, mostly based on graft morphology, that non-neural epiblast has a A/P restricted competence at the time of the graft.
Overall, the manuscript shows a series of nice time-lapse studies but does not provide enough new understanding to the field. The data systematically lacks quantification and the text is difficult to follow in many places. Lack of quantification (even no mention of number of embryos analysed) and specific AP markers substantially weaken the authors' conclusions.

The new version of the manuscript was written to emphasize new findings gained by live imaging of epiblast cells after st. 4, providing quantified time-resolved cell migration data. In addition, the significance of the data from the simultaneous live recording of both grafted tissues and responding epiblast cells was discussed more deeply. Besides, higher- magnification and higher-quality photo images were used to confirm brain portion identification.

Reviewer 2 Comments for the Author...

Specific comments:
- *The cell tracking reproduces the movements already published by Weijer and others. Furthermore,claims of collective migration are not supported by any quantification of cell movements.*

As stated in the Introduction section, earlier studies on epiblast cell migration were almost entirely concerning the primitive streak formation in the posterior epiblast and before st. 4. Our live imaging of epiblast cell migrations after st. 4 revealed a wide-range anterior epiblast convergence to the midline in the head tissue formation in the anterior epiblast and posterior cell migration along the midline in the posterior epiblast, providing brand-new information concerning the epiblast dynamics after st. 4.

In the previous manuscript, we presented only long tracks of migrating cells to show long-distance cell convergence. The new manuscript shows more time-resolved short tracks quantifying cell migration rates and directions and their changes (Fig. 2; Fig. S3). These new analyses also demonstrated that cells in an area migrate coherently (in the same direction with the same velocity), maintaining their spatial relationships and confirming their collective migrations.

- *The authors down-play the difference between their neural plate fate mapping results and the ones published by the Puelles lab at the same stage. The difference is not only the position of the anterior neuralplate border (a difference that is very big: 250 vs 500 microns!). The cells forming the forebrain in the Puellesfate-map are also located near the node forming a V shape field of cells. The authors do not find cells in front of the node participating to the forebrain area. In their case, the first cells anterior to the node are midbrain cells. This is a conceptually very meaningful difference that needs to be verified by further mapping and properly discussed. The Puelles data is weak on these medial cells so it is important to unambiguously determine the fate of these cells.*

We appreciate the reviewers for pointing this out. As detailed in response to Reviewer 1's point iv above, our data with single-cell resolution provide new insights into the head precursor distribution (Fig. 2; Figs. S3 and S4), allowing the revision of the Fernández- Garre map. The relevant points are elaborated in the Discussion section.

- *The finding that head ectoderm cells transiently express sox2 is interesting and suggests indeed apotential for neural fate that is switched on if node or AME is grafted. However, the link between sox2+ectoderm and competence to respond to node signals is only correlative.*

© 2022. Published by The Company of Biologists under the terms of the Creative Commons Attribution License (https://creativecommons.org/licenses/by/4.0/).

In the new version of the manuscript, this point was discussed in conjunction with *Dlx*5 (head ectoderm marker) data of Pera et al. (1998) and *Sox2* N2 enhancer activity substantiating the model we have reached.

- *Grafting in the lateral epiblast (future head ectoderm) suggests that these are committed to their anterior-posterior position as early as stage 4/5. More precise AP markers would be needed to ascertain thisfinding.*
- *Grafts of anterior mesoderm rather than node reveal that anterior mesoderm alone is sufficient to induceneural tissues. This could provide some new understanding if the authors would use a series of markers identifying the set of AP identities induced by these cells.*

We have routinely terminated the embryo cultures at st. 8 to 9 to correlate live imaging and in situ hybridization data, enabling backtracking brain portion precursors. The number of the brain regionality markers is limited at these stages; many come up only after st. 10. For example, En2, which encompasses the MB and HB, started its expression around st. 8, but the expression level was too low to withstand the multicolor in situ hybridization condition. In contrast, the lateral bulging of the forebrain is a reliable landmark and was used to identify the FB/MB boundary. Larger and better-quality images of in situ-hybridized embryos were used in the new version of the manuscript to ascertain our brain portion assessments.

Figures comments
- *Figure 1: Legend needed to understand the colours of each line representing cell trajectories.*

We added an explanation for this.

- *Figure 4 should mention how many grafts were done for each of the a-f positions and present markeranalysis for each position to ascertain AP identities of the graft-induced CNS.*

The numbers of grafts producing similar results are indicated in the new version of the manuscript. Fig. 7 focuses on AP marker analysis

- *Figure 5 is very difficult to understand and needs quantification.*

The problem was mainly caused by the superimposition of fluorescence and bright-field images. Brighter fluorescence images in the new version of the manuscript allow the readers to see the cell convergence movements. The new Movies 5 to 8 also vividly visualized the anterior epiblast cell gathering around the ectopic AMEs. In Fig. S8, some quantifications of the cell convergence movements toward the graft-derived AME are provided.

- *Figure 6 has same issues as Figure 4*

The number of embryos in the analysis (AME-grafted and hybridized for *Otx2* and *Gbx2* expression) was 13, as indicated in the main text. AP domain assessment was improved by the provision of higher-magnification and better-quality panels, as indicated above.

Reviewer 3 Advance Summary and Potential Significance to Field...

This study addresses how the anterior neural tube/early brain arises in the chick embryo over the period HH5-HH9. A key strength of the study is that the authors perform real time image analysis ofcell movements during neural plate formation/neurulation, after eGFP labelling. The authors showthat in normal development, epiblast cells converge and extend anteriorly to form the neural tube. By 'back-tracing' cells from their final destination at HH9 to their origin at HH5, the author develop amap to show the position of forebrain, midbrain and hindbrain precursors, and of head ectoderm precursors at HH5. This leads them to suggest that epiblast cells have the potential to give rise toneural tube or head ectoderm, and that fate is determined by proximity to the axis. To identify a potential role for axial mesendoderm, the authors graft nodes (capable of giving rise to anterior mesendoderm (AME)) to ectopic positions and show that host-derived secondary neural tube-like structures form around the differentiating grafts. The authors suggest that the type of neural tissue formed is dependent on the A-P position of the graft (anterior > forebrain-like; mid >

© 2022. Published by The Company of Biologists under the terms of the Creative Commons Attribution License (https://creativecommons.org/licenses/by/4.0/).

hindbrain- like; posterior > posterior neuraxis/floor plate?). Finally the authors perform a technically challenging experiment, combining grafting with live-imaging to try to argue that the AME directs surrounding head precursors to converge and develop into the brain.

Previous studies have generated fate-maps of the chick neural tube at HH4, have shown the abilityof the Node and node-derived structures (including the AME) to promote ectopic neural tissue, andhave suggested that epiblast cells can generate neural or ectoderm tissue. The state-of-the-art techniques (sparse labelling/live imaging) used in this study confirm previous fate-maps and ideas on epiblast cell potential, but are not necessarily novel findings.

In the new version of the manuscript, we have extended our single-cell analysis of the brain portion fate map and revised the previous map based on grafting ~125 µm tissue plugs. In addition, we have emphasized the significance of live imaging of the events that occur both in the grafted tissues and responding epiblast cells, which are presented in new Fig. S6 and Fig. 6.

The key advance, therefore is toshow that anterior regions of the neural tube develop through a mechanism that involves convergence towards the AME. A second key advance is the very different patterns of cell movements, anterior or posterior to Hensen's node. This observation is interesting, but not followed up in the work.

In the Introduction section of the new version of the manuscript, we made it clear that earlier studies on the epiblast movement were performed concerning the primitive streak formation before st. 4 and mainly in the posterior epiblast. The cell migration pattern analysis of the epiblast after st. 4 is novel, e.g., the brain-forming convergence in the anterior epiblast highlighted in this study and the massive posterior cell migration along the midline. These cell movements were analyzed in detail, as indicated in response to this reviewer's point 1.

Reviewer 3 Comments for the Author...
Despite its novelty, the study has a number of limitations.

1. Fig 1, Movies 1-3: the data demonstrates, very nicely, that there is a general cell movement towards to midline and then anteriorly (the authors term this collective migration). But looking at individual cell tracks, it is hard to be certain that this is a convergence to the AME. First, most of the cells imaged end up in the dorsal aspect of the neural tube. Second, looking at the tracks of individual cells (Fig 1A panel (b); Fig 1B pane;
(b) and Movie 2, they appear to converge towards themidline, then to track parallel to this.

While I agree that cells are converging towards the general midline, this does not necessarily argue for a convergence of cells toward the axial AME. The collective migration could be driven through other means.

In the previous manuscript, we showed long tracks of cells to show that epiblast cells migrate long distances toward the midline. However, this way of presentation alone was not successful in delineating the two modes of cell migrations, convergence toward the midline and anterior cell movements close to the midline. In the new manuscript, we also showed time-resolved short tracks to resolve the two modes of movement (Fig. 2, Fig. S3). These new data also showed that during the convergence toward the midline of anterior epiblast cells, cells in an area migrate coherently (in the same direction with the same velocity, maintaining their spatial relationships), featuring the collective movement.

2. Fig 2: The authors trace back the trajectories of individual cells, to determine the position of forebrain, midbrain and hindbrain precursors at HH5 and HH6. This relies on having a clear way to define the hindbrain, midbrain and forebrain at HH9. The authors use Gbx2 and Otx2 to define the HB/MB boundary (note the Otx2 labelling cannot be detected in Fig 2A (a) - so this needs to be repeated). However, they then suggest that they 'copy these boundaries on the SN-EGFP image of the same embryo' (bottom line p6). This does not seem to be the case: comparison of Fig2A panels (a) and (b) does not show the MB/FB boundary at the same position. This makes it very difficult tojudge the FB/MB data shown in the rest of this figure (panels B and C).

© 2022. Published by The Company of Biologists under the terms of the Creative Commons Attribution License (https://creativecommons.org/licenses/by/4.0/).

Live embryos had the ventral bending of the anterior head. In the flat-mount of in situ hybridized specimen, this anterior head portion is anteriorly extended so that the forebrain part of the embryos looks different in Fig. 2(A) [new 3(A). These embryos were aligned using the somites as initial landmarks, as detailed in response to the following point.

3. Fig 3: This figure (text and fig legend) is hard to follow. In particular it is not clear how the finalposition of each 'back-tracked' cell was determined. The authors need to explain this and justify how they decided if a cell was in the neural tube, the dorsal neural tube or the proximal headectoderm. This justification needs to be applied to Fig 2 as well.

To explain this point by point:

-If I understand correctly, panel (A) shows the position of precursors that will end up in the neuraltube. Where has the data come from? It is not the same as that shown in Fig 2B panel (b)?
-Panel B shows the same embryo: this time the dots show the position of precursor cells that willend up in the 'dorsal brain/proximal head ectoderm' Again – where has the data come from?
-The authors need to show an example, similar to those shown in Fig 2 to show that they candistinctly trace back cells that end up in the neural tube versus the dorsal neural tube/head ectoderm. Without having such higher resolution data I am struggling to understand the data inpanels A and B, and so struggling to understand how Fig 3 adds to previous studies.

A section was added in Materials and Methods to address the concerns of this reviewer, which reads:
Tracing the brain portion boundaries at st. 9 of SN-labeled embryos back to st. 6 and st. 5
The brain precursors at st. 6 were distributed in the bilateral epiblast zones 200 μm from the embryo axis and between the forming anterior ectodermal ridge and the original node position, as confirmed by repeated forward tracking. These labeled brain precursors became a part of the folding neural plate and closing neural tube, where fluorescence from the majority of the ventrally located precursors gradually diminished, and fluorescence-labeled dorsal neural tube cells and overlying ectoderm cells, derived from immediately outside the st. 6 brain precursor field, were detectable at st. 9, when the anteriormost portion of the forebrain was bent ventrally. The embryo was fixed immediately after the SN-EGFP recording and hybridized in situ for *Otx2* and *Gbx2* expression. The flat-mounted in situ hybridization image was then aligned in register with the image of the SN-EGFP-labeled embryo using somite positions. The coincidence of other morphological features validated the alignment. The FB/MB boundary where anterior FB welling occurs, the MB/HB boundary marked by the *Otx2*/*Gbx2* transition, and the HB/SC boundary defined by the posterior end of neural tube *Gbx2* expression were copied on the st. 9 SN-EGFP images. These boundaries were traced back, maintaining the relations to brightly labeled landmark cells to st. 8. Backtracking from st. 8 to 6, more centrally located cells gaining marked fluorescence provided additional landmarks for brain portion boundaries, and the portion boundaries were traced back to st. 6. SN-EGFP-labeled cells in the FB, MB, and HB compartments of the brain precursor field at st. 6 were individually traced back to determine their positions at st. 5.

4. Fig 4: Please provide examples of real image of acute grafts – to show the position and size of thegraft relative to the 'brain map' at HH4-5 (ie relative to the dots shown in Fig 3). The schematic shown in Fig 4A is not sufficient.

We appreciate these suggestions. The new Fig. 5(A) and the related figures show the snapshots of the node grafts immediately after the operation. The graft position summary schemes included the brain precursor map.

5. (see also point 9) Fig 4: The authors need to use markers to confirm the composition of cells thatdifferentiate from the Node grafts – ie markers that confirm that AME and not notochord, differentiate when grafts are positioned anteriorly, and that notochord, and not AME, differentiatewhen grafts are positioned posteriorly (Fig 4Ba-f).

We first determined the anteriorly and posteriorly extending tissue from the node graft as the AME and PNC, respectively, based on the live recording of these tissues. They differ in the direction and timing of tissue extension, and the morphology. The conclusion was that the node graft developed only into the extended AME when grafted into the anterior epiblast field and only into the PNC by

© 2022. Published by The Company of Biologists under the terms of the Creative Commons Attribution License (https://creativecommons.org/licenses/by/4.0/).

grafting into the posterior epiblast field. We have provided detailed data for these conclusions in the new Fig. S5 and relevant explanations in the main test.

The AME and the posterior notochord commonly express the representative transcription factor genes *Foxa2* and *T* (*Brachyury*).

6. (see also point 9) Fig 4: Similarly, markers that distinguish between forebrain, midbrain, hindbrainand spinal cord need to be used to confirm the types of brain region that develop ectopically.

We repeat here the response to a point to Reviewer 2. We have routinely terminated the cultures at st. 8 to 9 to correlate live imaging and in situ hybridization data. The number of the brain regionality markers is limited at these stages; many come up only after st. 10. For example, *En2*, which encompasses the MB and HB, started its expression around st. 8, but the expression level was too low to withstand the multicolor in situ hybridization condition. In contrast, the lateral bulging of the forebrain is a reliable landmark and was used to identify the FB/MB boundary. In the new version of the manuscript, higher-magnification and better-quality images of in situ-hybridized embryos were used to ascertain our brain portion assessments.

7. Fig 4 panel C: The idea that secondary brain development occurs at the expense of primary brainis really interesting, but raises many questions. Are the authors suggesting that there is a limitedsupply of cells with the potential to give rise to the brain? How does this fit with the idea that epiblast cells have potential to give rise to brain? This is a key point
- ie the origin of the cells that generate secondary structures - and ties into the questions raised regarding figs 2 and 3.
8. Fig 4 panel C: Sections are needed to show the extent of the missing dorsal tissue - it is notpossible to see this in the wholemount view.

In the new version of the manuscript, histological sections through two embryos showing dorsal defects are shown. In the previous version of the manuscript, we argued for the occurrence of the competition of the supply of the brain source from the epiblast region flanked by two developing brains. We think this kind of competition between two developing brains occurs on many occasions. However, histological sections suggested that topological constraints additionally contribute to dorsal closure defects. In the new manuscript focusing on the merit of live imaging, we feel that the above competition issue needs to be more rigorously tested by imaging-based analyses. Therefore, we reported dorsal closure defects without mentioning the possible causing mechanisms.

Additionally, while revising the figure panels, we found a mismatch between a histological section [current Fig. 5 (B)(d)] and the whole-mount image of the embryo [Fig. 5(E)]. Therefore, Fig. 5 (B)(d) panel was replaced with the correct embryo image.

9. Fig 6: Assertions as to development of particular brain regions are based partly on morphologyand partly on expression of Otx2 and Gbx2. The Gbx2 labelling is informative, but expression of Otx2 cannot be detected. The authors need to show improved in situs, and add probes that distinguish between FB and MB.

The *Otx2* hybridization signal color is orange. We have enlarged and improved the photo qualities of the panels in Fig. 6. The problem of the limited gene expression markers at st. 8 to 9 are explained above. The lateral bulging of the forebrain is a reliable landmark and was used to identify the FB/MB boundary.

10. The experiment to combine eGFP labelling, with grafts and real time imaging is technically extremely challenging, and the authors should be congratulated on achieving this. Frustratingly, though, it was still difficult to work out cell movements. The eGFP labelling is weak at a time whenkey events could be occurring and the image resolution was quite low. The authors conclude that precursors close to the graft start to converge on it, whereas those 'in the remaining region' (I amnot sure what this refers to?) converge on the host AME. But this was difficult to see. Largely, it wasimpossible to see how the cell movements after grafting related to those in the normal embryo. This makes it difficult to tie together the in vivo observations and the grafting studies. This significantlydetracts from the overall impact of the paper.

© 2022. Published by The Company of Biologists under the terms of the Creative Commons Attribution License (https://creativecommons.org/licenses/by/4.0/).

11. Likewise, it is difficult to interpret the cell movements in Fig 5B. It is difficult to know whetherthis is a convergence of cells towards the grafted tissue.

As stated in the response to Reviewer 2, the problem was mainly caused by the superimposition of fluorescence and bright-field images in the previous version of the manuscript. In the new version, brighter fluorescence images in isolation are presented in the new Figure panels and new Movies, clearly showing the cell convergence movements.

12. Fig5C and D: in these instances, where HH5 AME tissue has been grafted (instead of HH nodes that can differentiate to AME) it is difficult to make out the type of host tissue that develops ectopically – these are labelled as FB/MB/HB – but independent markers are needed to confirm this.

In the new version of the manuscript, higher-magnification and higher-quality images are presented in the panels [e.g., new Fig. 7(B)] to show secondary brain portions more clearly.

Minor comments
1. It would be helpful to the reader to better define the AME: ie the region of mesoderm and endoderm that has extended from the Node by HH5 and that will differentiate into prechordal mesendoderm, prechordal mesoderm and head process notochord.

The AME is explained in the new manuscript.

2. The Introduction needs some changes as it begins to introduce results: these should go at beginning of results section.

The Introduction section was revised entirely to better present the novelties of this study.

3. Fig 3A legend: please clarify that dots refer to data generated here and show the position at HH5,whereas shaded regions refer to a previous fate-mapping study (Fernandez-Garre) and show the position at HH4.

These are explained in the new Fig. 4(A).

4. P7: 'where relative position was … conserved' from st 6 (not in st 6)

Corrected.

5. Fig 4 panel C: please switch orientation so that it is obvious that this is an enlargement of Fig 4Bpanel (b).

Fig. 5 (previous Fig. 4) panels were revised and rearranged.

© 2022. Published by The Company of Biologists under the terms of the Creative Commons Attribution License (https://creativecommons.org/licenses/by/4.0/).

Original submission

First decision letter

MS ID#: DEVELOP/2021/199999

MS TITLE: Live imaging avian epiblast migration and interacting anterior mesendoderm in brain development

AUTHORS: Koya Yoshihi, Kagayaki Kato, Hideaki Iida, Machiko Teramoto, Akihito Kawamura, Yusaku Watanabe, Mitsuo Nunome, Mikiharu Nakano, Yoichi Matsuda, Yuki Sato, Hidenobu Mizuno, Takuji Iwasato, Yasuo Ishii, and Hisato Kondoh

I have now received all the referees' reports on the above manuscript, and have reached a decision. The referees' comments are appended below, or you can access them online: please go to BenchPress and click on the 'Manuscripts with Decisions' queue in the Author Area.

As you will see, the referees express considerable interest in your work, but have some significant criticisms and recommend a substantial revision of your manuscript before we can consider publication. I would particularly draw your attention to the comments about the activity of node derived AME. Investigating when AME acquires signalling needed for convergence-extension/neurulation seems a constructive suggestion by Referee 3. Additional grafts with quail st 5 fragments just posterior to AME and assaying surrounding epiblast movements and identity would also strengthen your conclusions.

If you are able to revise the manuscript along the lines suggested, which may involve further experiments, I will be happy to receive a revised version of the manuscript. Your revised paper will be re-reviewed by one or more of the original referees, and acceptance of your manuscript will depend on your addressing satisfactorily the reviewers' major concerns. Please also note that Development will normally permit only one round of major revision.

We are aware that you may be experiencing disruption to the normal running of your lab that make experimental revisions challenging. If it would be helpful, we encourage you to contact us to discuss your revision in greater detail. Please send us a point-by-point response indicating where you are able to address concerns raised (either experimentally or by changes to the text) and where you will not be able to do so within the normal timeframe of a revision. We will then provide further guidance. Please also note that we are happy to extend revision timeframes as necessary.

Please attend to all of the reviewers' comments and ensure that you clearly highlight all changes made in the revised manuscript. Please avoid using 'Tracked changes' in Word files as these are lost in PDF conversion. I should be grateful if you would also provide a point-by-point response detailing how you have dealt with the points raised by the reviewers in the 'Response to Reviewers' box. If you do not agree with any of their criticisms or suggestions please explain clearly why this is so.

Reviewer 1

Advance summary and potential significance to field

The authors of this paper use a combination of cell tracing and grafting to investigate the early brain fate map in the chick embryo. The first part of the describes an analysis of sparsely GFP labelled epiblast cell during the early stages of brain formation in stage HH3-HH8 embryos. The cells are manually tracked and the trajectories analysed. As expected the epithelial cells show highly coordinated displacements patterns respect to the observer moving from lateral to medial-anterior from above the level of the node and lateral to posterior below the level of the node. Although this is termed collective migration, the experiment does not provided insight in the underlying mechanism but the patterns suggests that it most likely results from large scale directed cell intercalations. The trajectories of the cells are compared with existing fate maps and although the observations roughly agree the single cell based analysis provided here indicates that there is

© 2022. Published by The Company of Biologists under the terms of the Creative Commons Attribution License (https://creativecommons.org/licenses/by/4.0/).

some degree of mosaicism of precursors in the various areas that was not captured in the exiting fate maps showing that the cells are not yet determined at this stage. Furthermore it is found that some brain tissue derives from area opaca epiblast.
The second part of the paper uses grafted labelled HH4 nodes or AME's to probe that developmental potential of various epiblast regions. It was shown/confirmed that HH4 nodes or AME's from later stages can induce secondary brains by influencing the fate of host tissue when grated in the anterior area pellucida.
It is found that brain identity of induced secondary brains depends on the position of the graft, resulting in a revised epiblast competence map (Fig.8A).
These experiments are generally well described and provide some useful new and higher resolution data. This is a thorough descriptive study on the origin and migration of epiblast cells during early brain development and an improved characterisation of their differentiation competence at the early stages of brain development as such will likely act as the basis for further studies.

On the other hand this study provides little new mechanistic insight in either the mechanisms that control the movement of the epiblast cells studied here, the identity of these signals controlling these movements or the mechanism that control the competence of the anterior epiblast tissue.

Comments for the author

minor points.
What mechanism(s) do the authors assume to underlies the displacement of the epiblast cells? Do the authors think that this is important in their interpretation of the results, for instance the observed mosaicism in the fate map?
It seems likely that the accumulations of cells seen around the grated nodes /AME's are due to the attraction of host mesenchymal cells rather an increase in density of epiblast cells, but it could be that an apparent increase in labelled cells is due to folding of the epiblast. How do the authors know that only cells in the epiblast are labelled? Is it possible that some of the labelled cells that move to the grafted node are actually mesenchymal cells? Are there any data on to show what is the case such as sections of induced structures?
It seems that the differences in the displacements of the epiblast cells are small and likely not statistically significant, using an appropriate non-parametric test. I would suggest to put in standard deviations and significance of differences in Fig 2B.
Is there any evidence that the rather complex multi-vector supernova system provides any better results than electroporation with a GFP expression vector which can also be readily observed after 3hrs. Is there any evidence for the supposed amplification loop working? This is all based on transient expression and it does not appear that titration with tetracycline is used to control the level of expression, if so it is not mentioned in the methods.
Carefully check figures and compare figure panels with text and legend (no panel fig 5Bf) mentioned in the last sentence of p10.

<u>Reviewer 2</u>

Advance summary and potential significance to field

This study addresses how the anterior neural tube/early brain arises in the chick embryo over the period HH5-HH9. A key strength of the study is that the authors perform real time image analysis of cell movements during neural plate formation/neurulation, after eGFP labelling, and in the context of mCherry grafted quail nodes/node-derived tissue. The authors show that in normal development epiblast cells converge and extend either anteriorly or posteriorly to form the neural tube. By 'back-tracing' anterior cells from their final destination at HH9 to their origin at HH5, the author develop a map to show the position of forebrain, midbrain and hindbrain precursors, and of head ectoderm precursors at HH5. To identify a potential role for axial mesendoderm, the authors graft nodes (capable of giving rise to anterior mesendoderm (AME) and posterior notochord) to ectopic positions and show that host-derived secondary neural tube-like structures form around the differentiating grafts. The authors suggest that the type of neural tissue formed is dependent on the A-P position of the graft (anterior > forebrain-like; mid > hindbrain-like; posterior > posterior neuraxis/floor plate?). Finally the authors perform a technically challenging experiment, combining

© 2022. Published by The Company of Biologists under the terms of the Creative Commons Attribution License (https://creativecommons.org/licenses/by/4.0/).

grafting with live-imaging to try to argue that the AME directs surrounding head precursors to converge and develop into the brain.

Comments for the author

The manuscript is much-improved, through both the re-write and the addition of the Movies. Movies 3 and 4 are especially useful and add significantly to the novelty of the paper, as well as show-casing the technically challenging experiments performed. I do still have fundamental questions however – which I raised in my original review and which I am still struggling to understand:
1.The focus of the paper is very much on the ability of the anterior mesendoderm (AME) to promote an anterior convergence of epiblast cells towards the midline, where they will give rise to the brain.
For instance, quoting from the figure legend to Movie 5: 'This movie shows (st4) node graft development into an ectopic gAME and the convergence of epiblast cells towards the gAME, which elicited secondary brain development'.
However, as very clearly shown (eg in Movies 3 and 4), st 4 nodes will give rise to both AME and posterior notochord, and notochord promotes a posterior convergence of epiblast cells to the midline (Movie 4 and Supp Fig 7 panel c). In other words, epiblast cells converge towards node-derived tissue (AME and notochord) – but those that track anteriorly become brain and those that track posteriorly become more posterior neural tissue. If this is the case, then it seems to me that an important novel point made by this paper is that AME goes anteriorly, notochord goes posteriorly and that their differentiation/extension in some way actively promotes epiblast convergence – and determines whether epiblast cells will go in an anterior or posterior direction. It is still hard to conclude that the driving force is entirely the AME and arguably the focus of the paper should be set within this broader context.
2. Despite the addition of details to the Methods section, I still do not see that the authors can definitively back-track cells to the neural tube, or to different brain regions (Figs 3 and 4) or to show the development of different brain regions after grafting (Fig 6). I do not agree that either the position of somites, or the anterior welling of the prosencephalon provide really solid landmarks. I would strongly encourage the authors to either provide additional in situ images – the Otx2 image is still difficult to detect, over general background, and despite their assertions, by HH9, a number of markers are being upregulated in different brain regions. If the authors do not have the resource to examine different brain regions after grafts, then they should be more speculative about the interpretation of their results.

<u>Reviewer 3</u>

Advance summary and potential significance to field

This version of the manuscript is improved and eliminate some of my concerns.
However, the conceptual progress made by this study is still a bit too vague and there is still some important gap between observations and conclusions.

Comments for the author

- Is the variability in the position of the anterior forebrain border from the node (now shown as ranging from 200 to 450 microns) due to very dynamic movements making each stage 4 embryo slightly different from each other, or caused by a more 'stable' variability from embryo to embryo? If the latter, the various neural plate territories would differ in size from embryo to embryo during neural plate stage. Please clarify.
- The results in Figure 5 and S6, S7 are showing that the ectoderm has restricted competence imposed by its already acquired AP identity (expected and already published in zebrafish and chick). It could all go to supplementary figures.
- The finding that it is the AME cell population emerging from the node that is triggering secondary 'brain' formation is interesting and novel. However, the anterior mesendoderm is in a normal embryo only located under the forebrain and part of midbrain anlage. When placed artificially in a posterior ectodermal position it induces hindbrain and even spinal cord. Why is this posterior induction AME-specific instead of triggered by any part of the maturing grafted node? Is the AME the only part that retain node abilities?

© 2022. Published by The Company of Biologists under the terms of the Creative Commons Attribution License (https://creativecommons.org/licenses/by/4.0/).

- The authors need to test whether the timing of ‘brain’ induction happening 3 hours after node grafting, defined based on convergence of cells towards node/AME is not actually just timing of convergence and/or neurulation and that identity would be induced by the node much earlier. In this scenario, the AME would have retained the induction property of the node at the same time as acquiring signalling needed for convergence-extension/neurulation (maybe Hh signalling?).
- Is the AME having this function or is any maturing node derivative such as the anterior notochord (derived from node later) also able to gather cells around it (although not having retained CNS-inducing ability)?

Some issues in the paper may be due to understandable language barrier as some sections of the manuscript are not clearly written.

- There are substantial issues of grammar that needs ironing out. For example;

Title: Live imaging OF avian epiblast migration and interacting mesendoderm DURING brain development”. Even grammatically correct the title is too vague. I would suggest “Live imaging of avian epiblast and anterior mesendoderm grafting reveals the complexity of cell dynamics during early neural and head ectoderm organisation.”

Summary statement “Live imaging of anterior epiblast cell movements AT the brain-forming stages in avian embryos was newly performed, revealing their long-distance migrations and interaction with anterior mesendoderm to form brain precursors. “

What is ‘their’ referring to? In the sentence it refers to movements which does not make sense. Better version: “First high-resolution description of live imaging of anterior epiblast cells at the brain-forming stages in avian embryos revealing their long-distance migrations and interaction with anterior mesendoderm to form brain precursors.”

The whole of the paper needs this kind of grammatical editing

- The titles of each results sections need to be checked for meaning as they often lack a verb and do not give the take-home message of the section

---

First revision

Author response to reviewers' comments

We thank the editor and reviewers for their constructive criticisms of the previous manuscript, which helped minimize ambiguities. We have revised the manuscript extensively on consultation with their points raised.

Following major revisions were made:

1. (In response to reviewer 1) GFP-immunostained embryo section data were provided in new Figure S1(C), which shows the SN-EGFP labeling of epiblast-derived tissues and demonstrates the tTA-dependent labeling densities.
2. (In response to reviewer 1) Figure 2 was revised to include statistical data.
3. (In response to reviewer 2) New Figure S4 was drawn to demonstrate how the brain portion boundaries were precisely traced back from st. 9 to st. 4.
4. (In response to reviewer 2) In situ hybridization data were improved by their photo recording using a high color temperature setting.
5. (In response to reviewer 3) Previous Figure 5 was moved to Supplementary Information to highlight live imaging-based approaches.
6. (In response to reviewer 3 and the editor). Analysis of the timings of the activities of the node derivatives to elicit cell convergence, comparing the node and AME grafts, was reported in the new Figure S10. This analysis was only briefly mentioned in the previous manuscript. The analysis indicated that graft-derived gAME rather than the grafted node provokes the convergence of anterior epiblast cells.
7. The texts and figure panels were rechecked to enhance accuracies. Particularly, AME and its derivatives, namely PP and ANC, were explained in more detail.

Itemized responses the reviewers’ points: Reviewer 1

– What mechanism(s) do the authors assume to underlies the displacement of the epiblast cells?

We first considered two mechanistic models. The first model was repulsion to BMP and/or nodal

© 2022. Published by The Company of Biologists under the terms of the Creative Commons Attribution License (https://creativecommons.org/licenses/by/4.0/).

signaling because the anterior mesendoderm (AME) and its derivatives secrete their antagonists. We transplanted COS cells expressing these antagonists singly or in combinations in st. 4 embryos, but none recapitulated AME's cell gathering activity. These results have been mentioned in the third chapter of the Discussion section. The second model was an attraction by Shh. However, we have not successfully demonstrated the cell gathering activity of Shh using smoothened agonist (SAG). We are still pursuing this issue.

– Do the authors think that this is important in their interpretation of the results, for instance the observed mosaicism in the fate map?

The reviewer presumably refers to the overlaps in the distribution of FB, MB, and HB precursors. However, these overlaps do not represent precursor mosaicism in an embryo. They are derived from the superimposition of data from four embryos shown in Figure S4, which differ in the AP extent of the brain domain precursors. To clarify, we have added the following explanation in the main text. "The distribution of brain portion precursors at st. 5 in four embryos (Fig. S5) indicated substantial embryo-to-embryo variations; the anterior extent of the precursor distribution from the node varied in the range of 210–450 µm, while the AP order of the FB/MB/HB precursor arrangement was conserved. This variation presumably reflects embryo-dependent differences in the timing of lateral and axial cell migration (Fig. S3). Thus, the superimposition of the distribution of brain portion precursors of the four embryos in Figure 4(A) displays the overlapping regions of different brain portion precursors, reflecting the variability in the distribution among embryos (Fig. S4) but not a mosaicism in an embryo."

– How do the authors know that only cells in the epiblast are labelled? Is it possible that some of the labelled cells that move to the grafted node are actually mesenchymal cells? Are there any data on to show what is the case such as sections of induced structures?

The DNA was injected into the space between the vitelline membrane and the epiblast and electroporated toward the epiblast. We prepared cross sections of the SN-EGFP–labeled embryos, as shown in the new Figure S1(C) panels. The epiblast-derived tissues, that is, the neural plate and head ectoderm, were labeled at high rates (>10%) in these sections, whereas endoderm or head mesenchyme were not labeled to a significant extent.

– It seems that the differences in the displacements of the epiblast cells are small and likely not statistically significant, using an appropriate nonparametric test. I would suggest to put in standard deviations and significance of differences in Fig 2B.

The data statistics were given in Figure S3, but as the reviewer has suggested, we have presented the standard deviations and P values in the main Figure 3. The differences in the cell migration rates in the regions "a" and "c" were statistically significant ($P < 0.01$) in the Mann–Whitney U test.

– Is there any evidence that the rather complex multi-vector supernova system provides any better results than electroporation with a GFP expression vector, which can also be readily observed after 3 hrs.

Supernova labeling has two significant advantages in tracking cells over many hours in embryonic development: (1) the labeled cells remain scattered over the period, facilitating tracking of individual labeled cells and (2) fluorescence intensity remains within a range over the period, allowing time-lapse recording using a single brightness range. The following panels compare the SN-EGFP and N2-mCherry fluorescence in the same co-electroporated embryo using brightness settings to detect fluorescence in 3 h in both channels. These demonstrate the above two advantages of SN-EGFP labeling.

© 2022. Published by The Company of Biologists under the terms of the Creative Commons Attribution License (https://creativecommons.org/licenses/by/4.0/).

Figure for reviewers 1

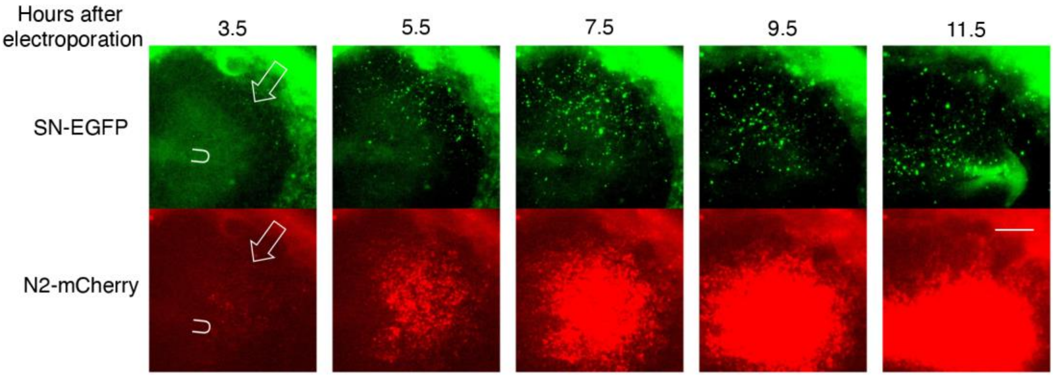

- Is there any evidence for the supposed amplification loop working? This is all based on transient expression and it does not appear that titration with tetracycline is used to control the level of expression, if so it is not mentioned in the methods.

Without including the tTA vector in the electroporation cocktail, very few cells, less than ten, were labeled by SN-EGFP in the epiblast of an embryo. This low rate of SN-EGFP-labeling depended on the TRE leakiness. A high cell labeling density sufficient to analyze epiblast cell migration was achieved using the tTA vector at 0.1 µg/µl. The labeled cell density increased further by using the tTA vector at 0.4 µg/µl while maintaining other components (presented in new Figure S1(C)). The subsequent tTA feed-forward loop was demonstrated in Luo et al. (2016) Supplementary Figure 3. Also, although the cell labeling rate was low in the electroporated chicken embryos without exogenous tTA, the labeled cells gained brightness comparable to the cases with exogenous tTA, indicating that the tTA feed-forward loop via vector (c) was in operation. Both exogenous tTA and feed-forwarding tTA likely contribute to the full activation of vector (c). The legend to Figure S1 was rephrased to indicate the above points.

Carefully check figures and compare figure panels with text and legend (no panel fig 5Bf) mentioned in the last sentence of p10.
We have revised this point and rechecked other parts.

Reviewer 2

However, as very clearly shown (eg in Movies 3 and 4), st 4 nodes will give rise to both AME and posterior notochord, and notochord promotes a posterior convergence of epiblast cells to the midline (Movie 4 and Supp Fig 7 panel c). In other words, epiblast cells converge towards node derived tissue (AME and notochord) – but those that track anteriorly become brain and those that track posteriorly become more posterior neural tissue. If this is the case, then it seems to me that an important novel point made by this paper is that AME goes anteriorly, notochord goes posteriorly, and that their differentiation/extension in some way actively promotes epiblast convergence – and determines whether epiblast cells will go in an anterior or posterior direction. It is still hard to conclude that the driving force is entirely the AME and arguably the focus of the paper should be set within this broader context.

A critical difference exists between the AME and the posterior notochord (PNC), which was demonstrated by grafting st. 4 nodes in the anterior and posterior epiblasts, as shown in Figures S7 and S8. Grafting nodes in the anterior epiblast resulted in the graft-derived (g)AME extension only, which resulted in cell gathering of proximal epiblast cells around the gAME, which in turn, resulted in secondary brain tissue development. By contrast, grafting nodes in the posterior epiblast caused only the PNC development without gathering cells around. To highlight these points, we added the following sentences in the text: “These observations indicated that (1) the node develops both the AME and PNC only at the original node position; (2) node grafts at anterolateral sites develop only AME tissue, whereas node grafts at posterolateral sites develop only PNC tissue.”

The Fig. S7(c) case does not involve any cell gathering. The host epiblast cells underlain by

© 2022. Published by The Company of Biologists under the terms of the Creative Commons Attribution License (https://creativecommons.org/licenses/by/4.0/).

the graft-derived posterior notochord developed only into the narrow strip of floor plate-like cells.

Besides, our unpublished data using SN-EGFP labeling at st. 3 and other studies have indicated midline convergence of the posterior epiblast to form the paraxial mesoderm occurred during st. 3 before the posterior notochord extension. This observation confirms that the posterior epiblast cells converge toward the midline, presumably the primitive streak, but not to the PNC.

Moreover, as briefly mentioned in the Results section: “Posterior epiblast cell migration at the postnode level in region (c) has not been characterized previously but likely contributes to the anterior spinal cord development, which will be reported elsewhere.”

– I still do not see that the authors can definitively back-track cells to the neural tube, or to different brain regions (Figs 3 and 4).

We have prepared new Figure S4 (appended below) to show how the st. 9 brain portion boundaries can be traced back to the st. 6 neural plate stage.

Figure S4. Tracking back the brain portion boundaries from st. 9 to st. 6 using SN- EGFP-labeled cells. A. Immediately after the termination of SN-EGFP recording, the labeled embryos were fixed and hybridized in situ for the analysis of *Otx2* (expressed in the FB and MB, orange) and *Gbx2* (expressed in the HB, purple) expression. The FB/MB boundary was determined as the posteriormost position of lateral expansion characteristic of the FB. B. The hybridized embryo images were aligned to the last frame of the bright field/SN-EGFP composite image. In the embryo shown in Figure 3, the last frame was the 90th at st. 9 with an imaging interval of 10 min. This embryo showed ventral curling of the FB region, which occasionally occurs in culture, whereas the hybridized specimen was mounted flat, with extension of the FB portion. The two embryo images were aligned in register using the somite positions and other morphological features as guides. These boundaries were marked on the SN-EGFP image or the 90th frame. C. These boundaries (HB/spinal cord, blue; HB/MB, cyan; and MB/FB magenta) were tracked back using SN-EGFP fluorescence image frames to earlier stages guided by landmark labeled cells encircled in the panels eventually to st. 6, as described in the Materials and Methods section. Note that the panel sequences show that ventral curling of the FB portion started after st. 7. In this figure, the frame excerpts are arranged so that the boundaries can be followed in these still images. In the actual procedure, however, positional changes of the landmark labeled cells were followed in all frames using a movie to accurately track the brain portion boundaries. The same procedure was used for all four embryos at st. 6 shown in Figure S5. The bar, 500 μm.

© 2022. Published by The Company of Biologists under the terms of the Creative Commons Attribution License (https://creativecommons.org/licenses/by/4.0/).

Figure S4

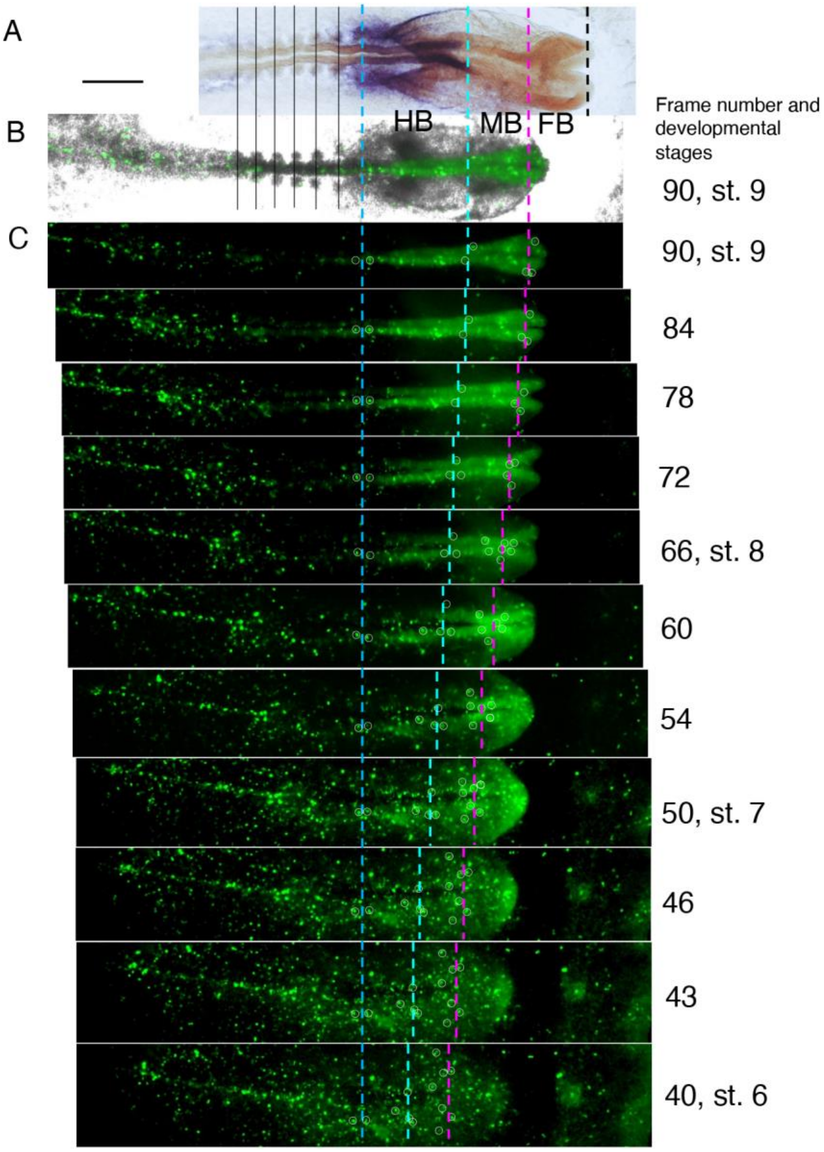

– I would strongly encourage the authors to either provide additional in situ images - the Otx2 image is still difficult to detect, over general background.

We have identified the cause of the yellowish background of the in situ hybridization images. It was caused by the low-color-temperature illumination used to capture these images. We have replaced all in situ hybridization images with high-color-temperature versions, which clearly show the orange Otx2 signals. An example is shown below.

© 2022. Published by The Company of Biologists under the terms of the Creative Commons Attribution License (https://creativecommons.org/licenses/by/4.0/).

Figure for reviewers 2

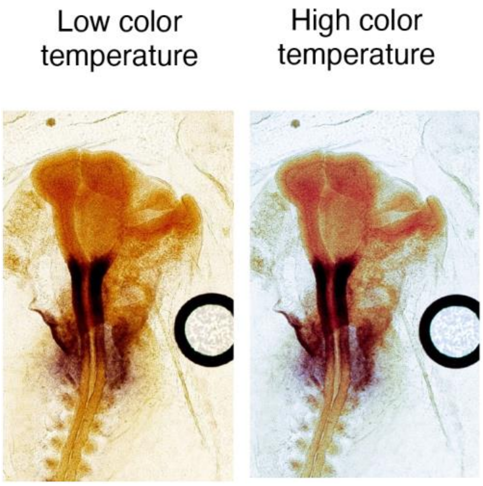

– Despite their assertions, by HH9, a number of markers are being upregulated in different brain regions. If the authors do not have the resource to examine different brain regions after grafts, then they should be more speculative about the interpretation of their results.

We believe that the initial regional specificity of the epiblast summarized in Figure 7 only grossly determines the secondary brain portions. Indeed, in the discussion section, we have stated, “However, these brain portion potentials are likely macroscopic and tuned via cell–cell interactions in the cell community.” No pan- forebrain or pan-midbrain markers are available. We believe it is fair to judge the Otx2-positive neural tissue with lateral bulging at st. 9 as the forebrain and Otx2-positive, posterior-positioned narrower neural tube as the midbrain, as far as we clearly state the criterion of the judgments. (The brain subdomain markers depend on the external signal inputs and various interactions within the brain tissues, mostly in the subsequent steps, which would complicate the data interpretation.)

Reviewer 3

– Is the variability in the position of the anterior forebrain border from the node (now shown as ranging from 200 to 450 microns) due to very dynamic movements making each stage 4 embryo slightly different from each other, or caused by a more 'stable' variability from embryo to embryo? If the latter, the various neural plate territories would differ in size from embryo to embryo during neural plate stage. Please clarify.

The variability in the position of the anterior forebrain border from the node reflects differences in dynamic movements among embryos. In the Results section, we have added the following sentences: “However, variations existed among embryos and even between embryo sides concerning the time axis- directed cell convergence began [e.g., the cell migration in the embryo left side in Figure 2(A) was ~1 h earlier than the right side; Fig. S3]. This kind of variation in the cell dynamics presumably contributed to embryo-dependent variations in the brain portion precursor distributions”

– The results in Figure 5 and S6, S7 are showing that the ectoderm has restricted competence imposed by its already acquired AP identity (expected and already published in zebrafish and chick). It could all go to supplementary figures.

We have accepted the reviewer’s suggestion to highlight imaging-based analyses and moved previous Figure 5 to supplementary Figures (Figure S8).

– The finding that it is the AME cell population emerging from the node that is triggering secondary ‘brain’ formation is interesting and novel. However, the anterior mesendoderm is in a normal embryo only located under the forebrain and part of midbrain anlage.

© 2022. Published by The Company of Biologists under the terms of the Creative Commons Attribution License (https://creativecommons.org/licenses/by/4.0/).

We found some misunderstanding concerning the use of AME. The reviewer refers to the prechordal plate (PP) as AME, whereas we have referred to the precursor for both PP and anterior notochord (ANC) as AME. At the beginning of the Introduction section, we have added: “The node (Hensen’s node) is formed at the anterior streak end around the beginning of st. 4; then, at st. 5, a part of the node develops into the anteriorly extending process, referred to as anterior mesendoderm (AME) in this study.” This clarification will resolve some concerns raised by the reviewer.

– When placed artificially in a posterior ectodermal position it induces hindbrain and even spinal cord.

The AME does not induce hindbrain at posterior epiblast levels, and never the spinal cord.

– Why is this posterior induction AME-specific instead of triggered by any part of the maturing grafted node? Is the AME the only part that retain node abilities?

As discussed below, the nodes do not elicit epiblast gathering.

– The authors need to test whether the timing of ‘brain’ induction happening 3 hours after node grafting, defined based on convergence of cells towards node/AME is not actually just timing of convergence and/or neurulation and that identity would be induced by the node much earlier. In this scenario, the AME would have retained the induction property of the node at the same time as acquiring signalling needed for convergence-extension/neurulation (maybe Hh signalling?).

We appreciate the reviewer and editor for raising the issue of timing regarding the activity of AME to elicit the surrounding epiblast convergence. We have reanalyzed the series of data presented in Figure 5 in the sequence of events that occurs on the grafted st. 4 nodes and st. 5 AMEs in early st. 4 chicken host embryos.

The results are schematically presented in the new Figure S10 appended below and summarized in the following new text in the Results section.

“As summarized in Figure S10, the local convergence of anterior epiblast cells onto the AME and subsequent neural tube development proceeded on the schedule of gAME development, occurring 4–5 h earlier when starting from AME grafts than node grafts. Analysis of the event timing indicated the following important points. (1) When the SN-EGFP labels became detectable at late st. 4, all grafted st. 5 AME tissues had elongated and already gathered proximal epiblast cells (N=7), indicating the competence of st. 4 epiblast cells to respond to the gAME. (2) Many grafted nodes remained without AME extension when SN- EGFP fluorescence became detectable. Despite the competence of the host epiblast at this stage, these node grafts did not gather proximal epiblast cells (e.g., Movie 6, N=7), indicating that the nodes lack the activity to elicit local epiblast convergence.”

Figure S10. Schematic representation of the sequence of events after the grafting of st. 4 nodes (N = 13) and st. 5 AMEs (N = 7) in early st. 4 chicken host embryos on the time scale of the host developmental stages and the hours after grafting. The host embryos were electroporated with SN-EGFP vectors, resulting in fluorescence signals around the end of st. 4 and starting the “SN-EGFP- detectable time zone.” The grafts were from mCherry-expressing transgenic quail embryos; their characteristic morphological changes are shown in magenta in fluorescence images, which are excerpts of data in Figure 5(A) (Movie 5) for the nodes and in Figure 5(C) (Movie 7) for the AME tissues. The green asterisks indicate the first moments that the cell-gathering activity of the grafted node/AME (gAME) was detected. A and P indicate the anterior and posterior sides of the grafts. Bar, 500 µm.
The grafted nodes maintained their original state with clear tissue demarcation for ~3 h and then started to extend the gAMEs, which was the first moment the cell- gathering activity of the grafted tissue could be detected. In many specimens (N=7), node grafts were still in the original state when entering the SN-EGFP-detectable time zone and did not show activity to gather cells around. Toward the end of st. 5 (~10 h after grafting), the gAME elongated, and was wider on the anterior side, and elicited convergence of proximal epiblast cells. Then, during st. 6, the AME differentiated into the wide and short PP and thin and long ANC.

© 2022. Published by The Company of Biologists under the terms of the Creative Commons Attribution License (https://creativecommons.org/licenses/by/4.0/).

The AME grafts were isolated from the st. 5 donor embryos during their elongation. When the SN-EGFP became detectable (green asterisk), surrounding epiblast cells had already gathered. This observation indicated that st. 4 epiblast cells have the potential to respond to and gather around gAME tissues. Subsequent developmental steps of the gAME proceeded on the graft-autonomous schedule,
~5 h earlier than the development of the node graft or host node. Approximately 9 h after AME grafting, when the host embryos were still close to the end of st. 5 when the neural plates were still forming, the epiblast cells that had gathered around the gAME tissue started neural tube development (Movies 7-8).
These observations indicate that the epiblast cells have a wide time window to respond to gAME tissues but not to nodes and develop into neural tissues following the time course of gAME development.

© 2022. Published by The Company of Biologists under the terms of the Creative Commons Attribution License (https://creativecommons.org/licenses/by/4.0/).

Figure S10

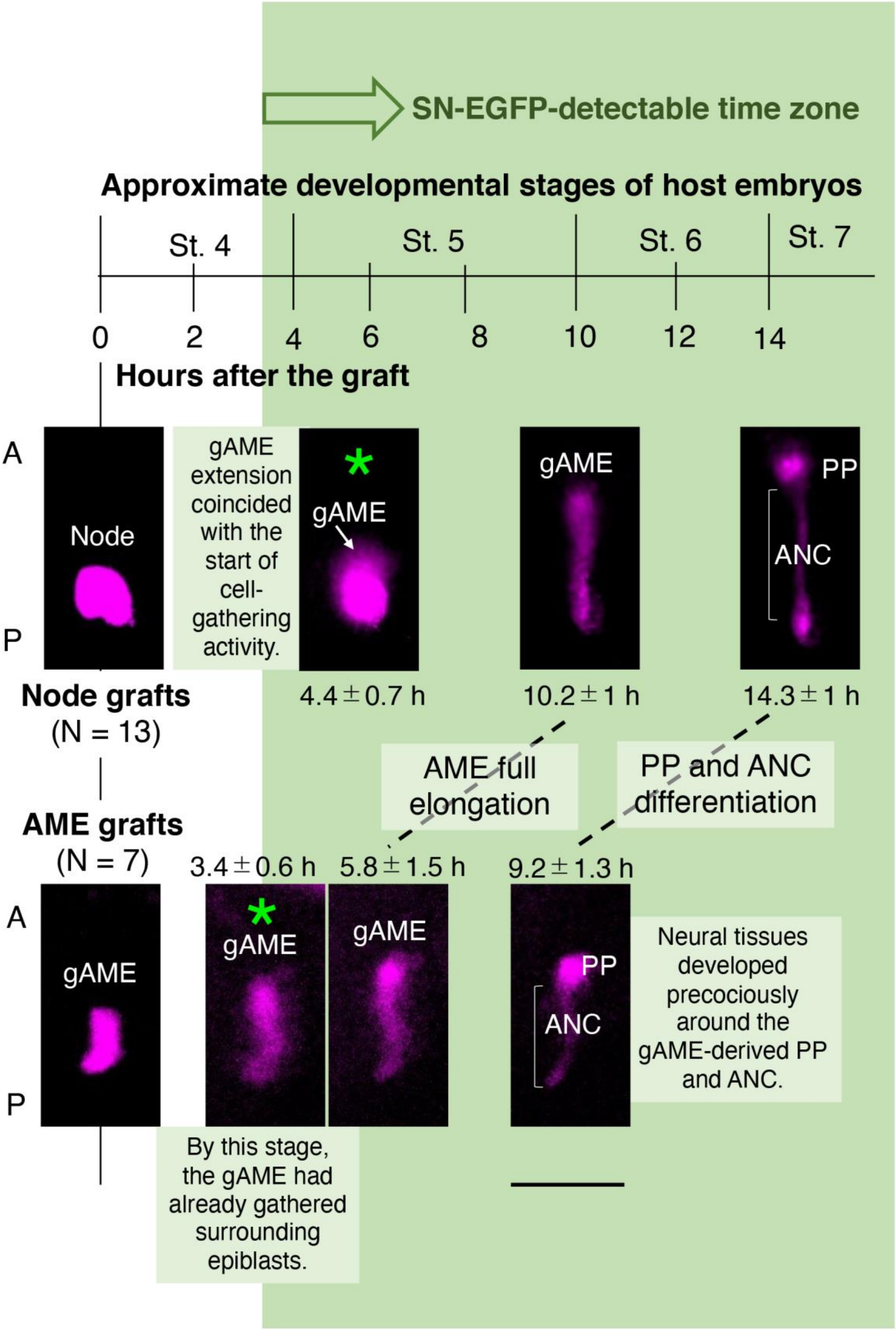

– Is the AME having this function or is any maturing node derivative such as the anterior notochord (derived from node later) also able to gather cells around it (although not having

© 2022. Published by The Company of Biologists under the terms of the Creative Commons Attribution License (https://creativecommons.org/licenses/by/4.0/).

retained CNS-inducing ability)?

This point was raised on the assumption that the node can elicit cell gathering, and the question was how the activity is carried over to later structures. However, as indicated above, the node does not possess such activity.

Some issues in the paper may be due to understandable language barrier as some sections of the manuscript are not clearly written.

– There are substantial issues of grammar that needs ironing out. For example; Title: Live imaging OF avian epiblast migration and interacting mesendoderm DURING brain development". Even grammatically correct the title is too vague. I would suggest "Live imaging of avian epiblast and anterior mesendoderm grafting reveals the complexity of cell dynamics during early neural and head ectoderm organisation." Summary statement "Live imaging of anterior epiblast cell movements AT the brainforming stages in avian embryos was newly performed, revealing their long- distance migrations and interaction with anterior mesendoderm to form brain precursors. "What is 'their' referring to? In the sentence it refers to movements which does not make sense. Better version: "First high-resolution description of live imaging of anterior epiblast cells at the brain-forming stages in avian embryos, revealing their long-distance migrations and interaction with anterior mesendoderm to form brain precursors."

We have improved the title and summary statement based on the reviewer's suggestions.

– The whole of the paper needs this kind of grammatical editing.

We have attempted to improve the text further.

– The titles of each results sections need to be checked for meaning as they often lack a verb and do not give the take-home message of the section

The titles of the results sections are now in the form of sentences.

Second decision letter

MS ID#: DEVELOP/2021/199999

MS TITLE: Live imaging of avian epiblast and anterior mesendoderm grafting reveals the complexity of cell dynamics during early brain development

AUTHORS: Koya Yoshihi, Kagayaki Kato, Hideaki Iida, Machiko Teramoto, Akihito Kawamura, Yusaku Watanabe, Mitsuo Nunome, Mikiharu Nakano, Yoichi Matsuda, Yuki Sato, Hidenobu Mizuno, Takuji Iwasato, Yasuo Ishii, and Hisato Kondoh

I have now received all the referees reports on the above manuscript, and have reached a decision. The referees' comments are appended below, or you can access them online: please go to BenchPress and click on the 'Manuscripts with Decisions' queue in the Author Area.

The overall evaluation is positive and we would like to publish a revised manuscript in Development, provided that the referees' comments can be satisfactorily addressed. The referees recognise that the revisions to your study strengthen the conclusions. Both Referee 2 and 3 indicate that the interpretation arising from the Otx2 labelling and what region of the nervous system this represents needs to appropriately caveated. In addition, Referee 3 suggests that cell tracing from data in Fig S11 would provide insight into the cell movements associated with grafts of the type shown in Figure 5.Please attend to all of the reviewers' comments in your revised manuscript and detail them in your point-by-point response. If you do not agree with any of their criticisms or suggestions explain clearly why this is so.

© 2022. Published by The Company of Biologists under the terms of the Creative Commons Attribution License (https://creativecommons.org/licenses/by/4.0/).

We are aware that you may currently be unable to access the lab to undertake experimental revisions. If it would be helpful, we encourage you to contact us to discuss your revision in greater detail. Please send us a point-by-point response indicating where you are able to address concerns raised (either experimentally or by changes to the text) and where you will not be able to do so within the normal timeframe of a revision. We will then provide further guidance. Please also note that we are happy to extend revision timeframes as necessary.

Reviewer 1

Advance summary and potential significance to field

see my previous review

Comments for the author

The authors have addressed most of the points that I raised on methodology and statistics in a satisfactory manner. The mechanism underlying the observed tissue deformations/movements and signals that control these remain to be resolved.

Reviewer 2

Advance summary and potential significance to field

The authors have gone some way towards addressing two of my concerns - indicating in more detail how the back-tracking was performed, and providing more detail on the different outcomes of grafts.

Further, the quality of the images is generally much improved.

There is still, however, little evidence for mechanism.

Comments for the author

I remain unconvinced by the Otx2 labelling - and would still prefer a more tentative/speculative conclusion about the interpretation of their results.

Reviewer 3

Advance summary and potential significance to field

I am satisfied by the improvements made to the manuscript. However, I would ask for further revision of the results in Fig 5 and S11. I would also suggest to edit the text to shorten it, avoiding repetitions and unnecessary details.

Comments for the author

- The results of quail grafts in Fig 5B and D show clear recruitment along an axis created between the anterior graft and a point inside the endogenous axis. The tracing shown in Figure S11 do not include this type of recruitment and only shows two embryo tracings (early and late stage 4 AME grafts), with the axis of the cell recruitment being the axis of the AME tissue. Figure S11 tracing should give us information on cell movements of the graft types shown in Figure 5. Tracing will be very informative in explaining how brain structure forming around the grafts are connected to the main CNS
- The labelling of the brain parts formed by the grafts have to stay rigorous.
When no marker is used, no FB, MB, HB should be used (Fig 5, S8 and S11). When markers are used, avoid to label Otx+ cells FB or MB according to the authors'
expectation (Fig. 6 and 7). The red tissue should always be labelled FM and blue HB with FM meaning Forebrain and/or Midbrain identity.

© 2022. Published by The Company of Biologists under the terms of the Creative Commons Attribution License (https://creativecommons.org/licenses/by/4.0/).

Second revision

<u>Author response to reviewers' comments</u>

We have revised the manuscript, following the points raised by the reviewers.

1. According to the suggestions of reviewers 2 and 3, the assignment of the anterior brain portions to the secondary brain based on in situ hybridization was changed. When the portion expressed *Otx2*, it was designated as FB/MB. Besides, when we further speculated the secondary brain's development into FB (anterior, with lateral bulging) or MB (posterior, thinner) based on their morphological features, we parenthesized these designations with the note that they were tentatively assigned on a morphological basis. This amendment was implemented throughout the texts, including main/supplementary Figures.
2. According to the suggestion of reviewer 3, Figure S11 was renewed using the newly performed cell trajectory analysis data. Analyses showed that the histogenesis of the primary and secondary brains was responsible for the progressive approach of the two brain tissues.
3. Figure 5 dealing with data related to Figure S11 was also simplified by moving some items to Figure S11, also clarifying the geometrical relationship between the primary and secondary neural plate areas.
4. Some observation details of biological significance yet not necessarily central to the main issue of this study were moved to supplementary data.
5. The text was re-edited to minimize repetitions, e.g., moving a paragraph in the Results section to the Discussion section.

Alterations in texts and figure legends to accommodate these changes are color-marked in a supplementary material for reviewers.

Specific responses to the reviewers:

Reviewer 2:

– I remain unconvinced by the Otx2 labelling - and would still prefer a more tentative/speculative conclusion about the interpretation of their results.

Response:
In the revised manuscript, Otx2-expressing secondary brain portions were designated as FB/MB. When we further speculated the secondary brain's development into FB (anterior, with lateral bulging) or MB (posterior, thinner) based on their morphological features, we parenthesized these designations with the note that they were tentatively assigned on a morphological basis. This amendment was implemented throughout the texts, including main/supplementary Figures.

Reviewer 3

– The results of quail grafts in Fig 5B and D show clear recruitment along an axis created between the anterior graft and a point inside the endogenous axis. The tracing shown in Figure S11 do not include this type of recruitment and only shows two embryo tracings (early and late stage 4 AME grafts), with the axis of the cell recruitment being the axis of the AME tissue. Figure S11 tracing should give us information on cell movements of the graft types shown in Figure 5. Tracing will be very informative in explaining how brain structure forming around the grafts are connected to the main CNS.

Response
Figure S11 was renewed using the newly performed cell trajectory analysis data. Analyses showed that the histogenesis of the primary and secondary brains was responsible for the progressive approach of the two brain tissues.

Corresponding paragraph in the revised Results section reads:

"When the epiblast gathering on gAME leading to the secondary neural plate development (encircled by broken lines) occurred next to the forming primary neural plate (inverted U in dotted lines) (N=14)[Fig. 5(A)(C)], the secondary brain eventually fused along its length to the primary

© 2022. Published by The Company of Biologists under the terms of the Creative Commons Attribution License (https://creativecommons.org/licenses/by/4.0/).

brain. In contrast, when these neural plates were remote, they developed into two separate anterior heads fusing at the secondary brain posterior end (N=12) [Fig. 5(B)(D)]. In all cases of secondary brain development, the primary and secondary brain axes pulled toward each other (Fig. S11), which caused the primary brain axis to form an angle to the immediately posterior trunk axis at stage 8 [θ in Fig. S11 (A), 15.6 ± 6.5 (SD) degrees, N=26].

To investigate the mutually pulling interplay between two forming brain tissues, short tracks of SN-EGFP-labeled cells on and between these tissues were analyzed similarly to Figure 2 around the three axes, i.e., the primary and secondary brain axes and an axis in the interposing head ectoderm positioned at the later developing brain- separating head ectoderm. The analysis shown in Figure S11(B)(C), using the same embryo data as in Figure 5(B)(D), indicated that two forming brains pulled each other solely by the action of neural tube histogenesis from respective neural plates. The folding of the two neural plates into the narrow tubes and recruitment of proximal head ectoderm precursors into the covering cell layer accounted for progressive proximation of the primary and secondary brains. The intervening region of the head ectoderm precursor area remained still during the development of the neural tubes at both ends, whereas, subsequently, a part of the head ectoderm partitioned the two head tissues (Fig. S11). Nevertheless, the posterior end of the secondary brain sustained a neural tissue connection to the primary brain, presumably because it is the axial level where the supply of the epiblast to the brain precursor through lateral cell migration was the most abundant [Figs. 1(B)(D), 2]."

The new Figure S11 is appended below.

Figure S11. Histogenesis of primary and secondary brains is responsible for the progressive proximation of the two brain tissues. Bars, 500 μm.
Definition of tissue axes used in the analysis (a) and secondary brain- dependent bending of the primary head axis relative to that of the trunk (b). (a) H, host (primary) brain axis; G, graft-dependent (secondary) brain axis; B, the axis of boundary head ectoderm partitioning the primary and secondary heads; T, the axis of the anterior trunk, with which the H axis made the angle θ. (b) Distribution of the H axis bending angle θ measured in 13 embryos of each group at st. 8; without graft, with node grafts, and with AME grafts. Without graft, a slight H axis bending occurred with an average of 3 ± 3 (SD) degrees occurring randomly with the situs. In contrast, the graft-dependent bending had the vertex facing the grafts, with an average angle of 16.4 ± 7.2 degrees with node grafts, 14.8 ± 6.0 degrees with AME grafts, and 15.6 ± 6.5 degrees with all node/AME grafts ($P < 0.001$ compared to data of embryos without graft using the Mann–Whitney U test).
(B) and (C) Analysis of trajectories of SN-EGFP-labeled cells around axes H, B, and G, during the brain histogenesis stages.
ix) An embryo with the node graft, the same as that used in Figure 5(B). (a)-(d) Bright-field images at stages 6^{+}, 7, 8, and 9. The original position of the embryo axis and the fully extended lateral level of the graft-derived AME at st. 6^{+} are indicated using broken and dotted lines, respectively, which were 1,120 μm apart. The primary brain was progressively displaced toward the secondary brain during st. 6^{+} (a) to st. 8 (c) by 450 μm. (a')-(d') and (a'')-(d'') Randomly colored trajectories of SN-EGFP-labeled cells over a duration of 75 min (3 frames for an interval of 25 min) drawn relative to one of the three axes, H, B, or G, with the most recent positions being indicated in white to show the direction of trajectories. Single white dots indicate the epiblast positions that remained relative to the axis chosen over 75 min. (a', a'', st. 6^{+}) In (a'), the area (i) covered the primary neural plate, where the cells showed anteromedial cell convergence around the H axis (44 ± 15 μm/h), which pulled surrounding cell sheets and caused passive cell movements in the area (ii) toward the H axis (47 ± 12 μm/h). (a") Consistently, cell movements in the corresponding area (iii) were negligible when measured using the B axis in the head ectoderm precursors. (b', b'', st. 7) As the neural tube developed, the cell convergence toward the H axis became more extensive in the area (iv) of (b') (50 ± 15 μm/h). In contrast, trajectories around the G axis in the area (v) of (b'') indicated a moderate axial convergence (26 ± 5 μm/h), presumably reflecting the smaller scale of secondary neural tube histogenesis.
(c', c'', st. 8) Trajectories relative to the H and B axes indicate an off-peak stage concerning the axial cell convergence in the neural tubes and the approaching of the two tubes. Mutual pulling of primary and secondary brain tissues at their posterior ends created bending of the H axis relative to the T axis, which was reflected in the leftward trajectories in the area (vi) in (c') (41 ± 13 μm/h). At this stage, the primary and secondary heads were partitioned by the head ectoderm

© 2022. Published by The Company of Biologists under the terms of the Creative Commons Attribution License (https://creativecommons.org/licenses/by/4.0/).

penetrating along the B axis [see (A)(a), B axis], confirmed by the axis-aligned cell migrations (vii, 29 ± 7 μm/h). (d', d'', st. 9) This embryo developed a particularly thick neural tissue bridge connecting the two posterior brain tissues [arrowhead in (d)]. This tissue bridge presumably enhanced traction between the two brains and created a large θ (~40 degrees) at st. 9, which was observed in 3 of 13 node-grafted embryos. This traction was reflected by the post-node lateral trajectories (viii, 52 ± 13 μm/h; ix, 51 ± 9 μm/h). These observations indicate that the primary and secondary brains approached during the stages 6^{+} to 8, as the consequence of 3D histogenesis of the neural tubes and associated head ectoderm displacements. Indeed, assuming that an initially 400 μm-wide primary neural plate [Fig. 3(B)] folded into a 160 μm-wide neural tube at st. 8, and that head ectoderm strips of 160 μm wide at both sides moved to cover the neural tube [Fig. S1(C)], the 280 μm distance shortening between the primary and secondary brain axes is predicted. The occurrence of the same events in a 240 μm-wide secondary neural plate would add 170 μm to the distance shortening, thereby accounting for the observed axis distance shortening of 450 μm from 1,120 μm (B)(a) to 670 μm (B)(c).

x) An embryo with the AME graft, the same as that used in Figure 5(D). (a)(b) Bright-field images at st. 7 and 8, when the unstructured secondary neural plate was reorganized into the symmetrical brain tissue (Movie 8). The tissue reorganization made the secondary brain align with the primary brain, which was observed in the 9/13 cases of AME graft-elicited secondary brain development. This tissue reorganization occurred additionally to the neural tube formation- dependent proximation of brain axes depicted in (B). (a')(b') Trajectories of SN- EGFP-labeled cells over 60 min (6 frames of 10 min intervals) around the H and B axes are shown. At st. 7, cell movements in the forming secondary brain toward the H axis were extensive (xi, 59 ± 19 μm/h), with the rates comparable to the axis-directed movements in the primary brain (x, 58 ± 15 μm/h). The tissue reorganization in the secondary brain continued to st. 9 (xii, 46 ± 13 μm/h).

© 2022. Published by The Company of Biologists under the terms of the Creative Commons Attribution License (https://creativecommons.org/licenses/by/4.0/).

Figure S11

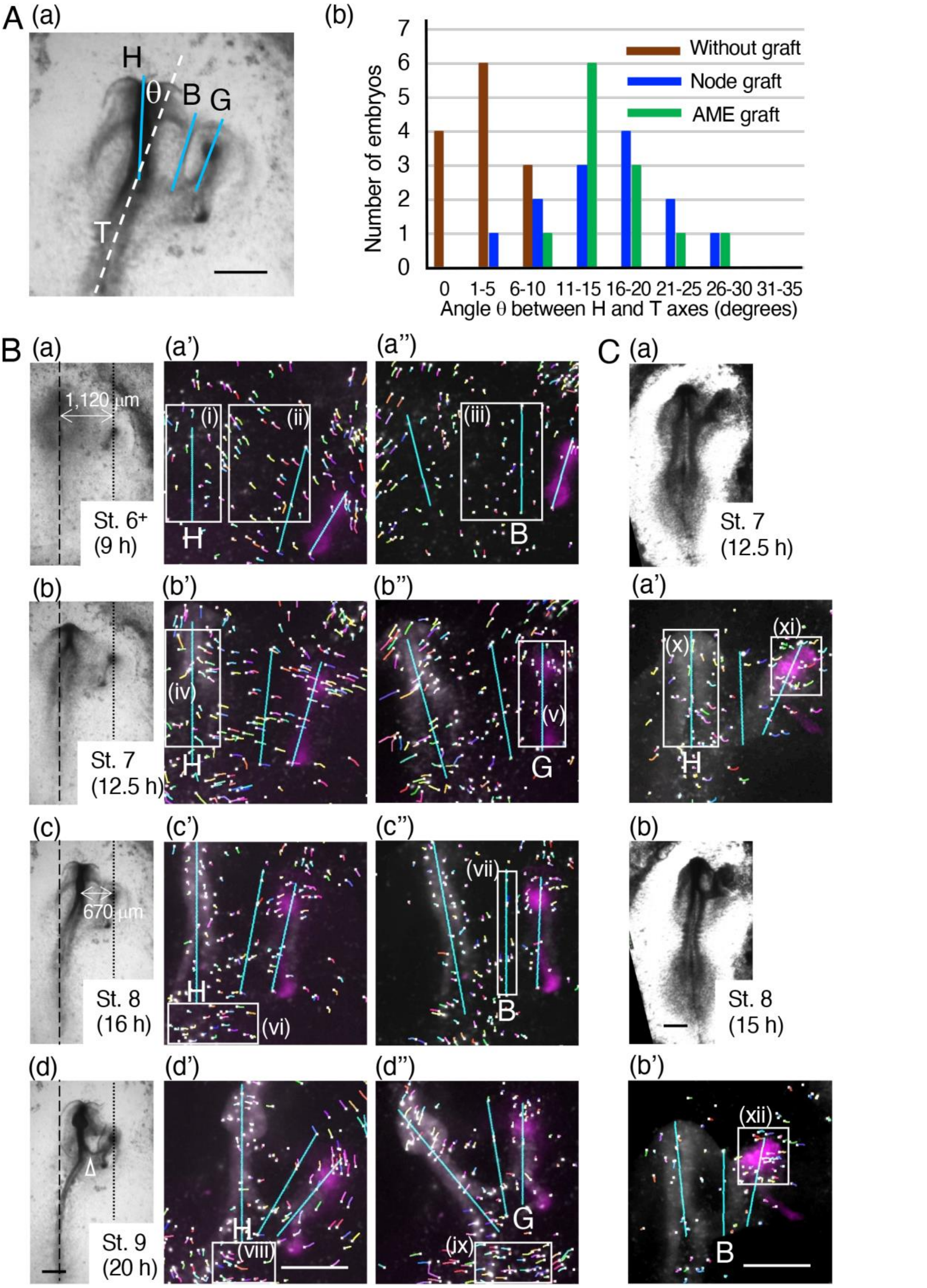

– The labelling of the brain parts formed by the grafts have to stay rigorous. When no marker is used, no FB, MB, HB should be used (Fig 5, S8 and S11). When markers are used, avoid to label

© 2022. Published by The Company of Biologists under the terms of the Creative Commons Attribution License (https://creativecommons.org/licenses/by/4.0/).

Otx+ cells FB or MB according to the authors' expectation (Fig. 6 and 7). The red tissue should always be labelled FM and blue HB with FM meaning Forebrain and/or Midbrain identity.

Response: (repeating the response to reviewer 2)
In the revised manuscript, Otx2-expressing secondary brain portions were designated as FB/MB. When we further speculated the secondary brain's development into FB (anterior, with lateral bulging) or MB (posterior, thinner) based on their morphological features, we parenthesized these designations with the note that they were tentatively assigned on a morphological basis.

– I would also suggest to edit the text to shorten it, avoiding repetitions and unnecessary details.

Response
We deleted following paragraphs from the main text. Additionally, we modified many details to avoid repetitions.

Overall, the data in Figures 5-6 and S8 indicate the following model: (1) The head precursors consisting of the anterior epiblast cells are bipotent in terms of being able to develop into the brain or head ectoderm. (2) Once the AME tissue develops, regardless of the host or graft origin, the AME-proximal anterior epiblast cells converge on the AME; the centrally positioned cells develop into the brain tissues, while those more distally positioned develop into the head ectoderm.
(3) The brain portions in the secondary brain tissues reflect the original regionality of the convergent anterior epiblast cells.

However, these brain portion potentials are likely macroscopic and tuned via cell-cell interactions in the cell community. Indeed, the MB/HB boundary is sharpened by mutually repressive interactions between the transcription factors OTX2 and GBX2 (Katahira et al., 2000; Martinez- Barbera et al., 2001).

The node grafts in the area opaca locally provide the cells with area pellucida traits (Pera et al., 1998; Pinho et al., 2011), and node grafting in the lateral area opaca gave rise to MB/HB portions of host origin (Storey et al., 1992), given within the L5 antigen-expressing zone (Streit et al., 1997). In contrast, node grafting at the anterior margin of the area pellucida elicited the development of the secondary FB portion, in addition to the MB and HB (Diaz and Schoenwolf, 1991; Knoetgen et al., 2000).

To test this model, we placed quail AME grafts anterior to the FB precursors [Fig. 7(B)]. The grafts within the FB potential domain elicited secondary FB portion development only. However, with AME grafting at a more anterior position encompassing three zones in a rare embryo with a large anterior area pellucida [Fig. 7(A), (B)(c)], all FB, MB, and HB portions developed in the secondary brain [Fig. 7(C)(c)], as predicted.

Third decision letter

MS ID#: DEVELOP/2021/199999

MS TITLE: Live imaging of avian epiblast and anterior mesendoderm grafting reveals the complexity of cell dynamics during early brain development

AUTHORS: Koya Yoshihi, Kagayaki Kato, Hideaki Iida, Machiko Teramoto, Akihito Kawamura, Yusaku Watanabe, Mitsuo Nunome, Mikiharu Nakano, Yoichi Matsuda, Yuki Sato, Hidenobu Mizuno, Takuji Iwasato, Yasuo Ishii, and Hisato Kondoh

I have now received all the referees reports on the above manuscript, and have reached a decision. The referees' comments are appended below, or you can access them online: please go to BenchPress and click on the 'Manuscripts with Decisions' queue in the Author Area.

© 2022. Published by The Company of Biologists under the terms of the Creative Commons Attribution License (https://creativecommons.org/licenses/by/4.0/).

The overall evaluation is positive and we would like to publish a revised manuscript in Development. As you will see, Referee 3 has a couple of minor issues that I would advise you attend to in your revised manuscript. Please detail these in your point-by-point response. If you do not agree with any of their criticisms or suggestions explain clearly why this is so.

We are aware that you may currently be unable to access the lab to undertake experimental revisions. If it would be helpful, we encourage you to contact us to discuss your revision in greater detail. Please send us a point-by-point response indicating where you are able to address concerns raised (either experimentally or by changes to the text) and where you will not be able to do so within the normal timeframe of a revision. We will then provide further guidance. Please also note that we are happy to extend revision timeframes as necessary.

Reviewer 3

Advance summary and potential significance to field

I am now satisfied by the modifications done on this second revision.

Comments for the author

I would advise the authors to put the description and interpretation of movements written in Fig S11 legend as a paragraph in the main text describing the results of Figure 5 and simplify the legend to simply what is needed to understand the figure, labels tracing and symbols.
I don't understand the sentence "Trajectories relative to the H and B axes indicate an off-peak stage concerning the axial cell convergence in the neural tubes and the approaching of the two tubes." in that S11 legend.

Third revision

Author response to reviewers' comments

We have revised the manuscript, according to the suggestions by reviewer 3, which we appreciate.

1. “I would advise the authors to put the description and interpretation of movements written in Fig S11 legend as a paragraph in the main text describing the results of Figure 5 and simplify the legend to simply what is needed to understand the figure, labels tracing and symbols.”
Response: We rewrote paragraphs in the chapter explaining the results of Figure 5 to include the interpretation of cell movements reported in Figure S11. We also simplified the legend to Figure S11 and compressed it to two thirds of its original length. The PDF file of the Supplementary Materials for reviewers includes the rewritten chapter with alterations shown in red, along with the revised Figure S11 and its legend.

2. “I don't understand the sentence "Trajectories relative to the H and B axes indicate an off-peak stage concerning the axial cell convergence in the neural tubes and the approaching of the two tubes." in that S11 legend.”
Response: The sentence was rephrased to: “Cell movements in and around the neural tubes diminished at st. 8.”

Supplementary Materials for Reviewers

Live imaging of anterior epiblast migration after node/AME grafting indicates that head precursor gathering on the AMEs is the first step of brain tissue development The above observations indicated that the nodes grafted in the anterior embryo region developed into the AME (Fig. S7) and that only under these conditions did the secondary brain tissues develop (Fig. S8). To investigate the link between these two events, we grafted mCherry-expressing quail node in the anterior region of SN-EGFP-labeled chicken host and live-recorded their developmental changes (N=13, Movies 5,6).

© 2022. Published by The Company of Biologists under the terms of the Creative Commons Attribution License (https://creativecommons.org/licenses/by/4.0/).

The case of quail node grafting in an anterior-lateral host position (N=5) is shown in Figure 6(A) and Movie 5. Snapshots of brightfield images indicating cell density changes (darker representing a higher density) [Fig. 5(A)(a)-(d)] and fluorescence images [Fig. 5(A)(a')-(d')] are compared. The grafted node [Fig. 5 (A)(a)(a')] anteriorly extended to form gAME tissue synchronously with hAME [Fig. 5(A)(b)(b')], which further differentiated into the PP and ANC (overlain by SN-EGFP-labeled cells) in 16 h [Fig. 5(A)(c)(c')]. The gAME¤proximal anterior epiblast cells converged around the elongating gAME, whereas those positioned more to the right did so around the hAME [Fig. 5(A)(b)(b')]; they individually formed neural plates and developed into brain tissues [Fig. 5(A)(c)(c')]. The gAME-centered secondary brain tissue eventually fused to the host brain [Fig. 5(A)(d)(d')].

Figure 5(B) and Movie 6 present the case of node grafting at the host node AP level at a remote position (N=8), where only the fluorescence image is shown. The gAME elongated 4–10 h after node grafting, while the surrounding anterior epiblast converged around it [Fig. 5(B)(b)], similar to Figure 5(A)(b)(b'). The gAME-centered secondary brain tissues [Fig. 5(B)(c)] united to the primary brain at their posterior parts [Fig. 5(B)(d)].

Movies (N=7) analogous to Movie 6, where the grafted nodes stayed without AME extension when the SN-EGFP-labeled cells became detectable, the node grafts did not show cell-gathering activities. Thus, the node graft-derived gAME rather than the grafted node provoked the convergence of anterior epiblast cells to initiate secondary brain development.

To confirm this conclusion, the isolated AMEs of st. 5 transgenic quail embryos were grafted at various host embryo positions [Fig. 5(C)(D), Movies 7,8] (N=7). The gAMEs quickly elongated anteriorly in 6 h and subsequently developed into the PP and ANC [Figs. 5(C)(b),(D)(b), S10], 4-5 h earlier than when starting from the node graft. The surrounding anterior epiblast cells converged on the gAME [Fig. 5(C)(b),(D)(b)], and developed in secondary neural plates [Fig. 5(C)(c),(D)(c)], while the host embryos were still at late st. 5 (Fig. S10, Movies 7,8). The secondary neural plates were structured into brain tissues after st. 7 of host development [~12 h from st. 4; Movies 7,8; Fig. 5(C)(c),(D)(c)], which was eventually fused to the host brain along the entire length of the secondary brain [Fig. 5(C)(d)] or at the posterior end [Fig. 5(D)(d)], similar to the case after node grafting [Fig. 5(A)(d'),(B)(d)].

As summarized in Figure S10, the local convergence of anterior epiblast cells onto the AME and subsequent neural plate formation proceeded according to the gAME development schedule, occurring 4-5 h earlier when starting from AME grafts compared to node grafts. An analysis of the event timing indicated the following. (1) By late st. 4, when SN-EGFP-labeled epiblast cells became detectable, all grafted st. 5 AME tissues had elongated and gathered proximal epiblast cells (N=7), indicating the competence of st. 4 epiblast cells to respond to gAME. (2) At the same developmental stage, many grafted nodes persisted without AME extension; they did not gather epiblast cells (e.g., Movie 6, N=7) despite the competence of the host epiblast, indicating that the nodes lack the activity to elicit local epiblast convergence.

When the epiblast gathering on gAME leading to the secondary neural plate development (encircled by broken lines) occurred next to the forming primary neural plate (inverted U in dotted lines) (N=14)[Fig. 5(A)(C)], these brains eventually fused along their lengths. In contrast, when these neural plates were remote, they developed into two separate anterior heads that fused at the secondary brain posterior end (N=12) [Fig. 5(B)(D)].

During the secondary brain development from the neural plate (st. 6) to the neural tube (st. 8), the primary and secondary brain axes approached each other (Fig. 5). This process was analyzed using embryos developing two separate brains (Fig. S11). In these embryos, at st. 8 (N=26), the primary brain axis (H) formed an angle of 15.6 ± 6.5 (SD) degrees to the immediately posterior trunk axis (T) with the vertex facing the secondary brain [Fig. S11(A)]. This axis bending occurred because the H axis was pulled toward the secondary brain axis (G) [Fig. S11(B)(a)-(c)]. The short tracks of SN-EGFP- labeled cells relative to one of the axes G, H, and B [the axis of brain-separating head ectoderm; Fig S11(A)(a)] were analyzed using the embryo data from Figure 5(B)(D) and Movies 6,8 [Fig. S11(B)(a')(a'')-(c')(c'')]. The analysis indicated that folding the two neural plates into narrower neural tubes and bringing the proximal head ectoderm precursors in the overlying cell layer caused the progressive proximation of the primary and secondary brain axes. On

© 2022. Published by The Company of Biologists under the terms of the Creative Commons Attribution License (https://creativecommons.org/licenses/by/4.0/).

the assumption that an initially 400 μm-wide primary neural plate at st. 6 [Fig. 3(B)] is folded into a 160 μm-wide neural tube at st. 8, and that head ectoderm strips of 160 μm wide at both sides move to cover the neural tube [Fig. S1(C)], a shortening distance of 280 μm between the primary and secondary brain axes was predicted. The occurrence of the same events in a 240 μm-wide secondary neural plate added 170 μm to the distance shortening, thereby accounting for the observed axis distance shortening by 450 μm, from 1,120 μm to 670 μm [Fig. S11(B)(a)(c)]. The region of head ectoderm precursor intervening the two approaching heads remained still during the process [Fig. S11(B)(a’’)(b’’)]. In the AME graft-elicited secondary brain development, structuring the brain tissue at st. 7 also contributed to the brain axes proximation [Fig. S11(C)].

To address the question of whether anterior epiblast cells gathering on gAMEs involved enhanced cell proliferation, node-grafted embryos were labeled using 5-ethynyl- 2′-deoxyuridine (EdU) (Fig. S12). gAMEs did not alter the proliferation rate of proximal epiblast cells that contributed to secondary brain development.

Figure S11. Histogenesis of primary and secondary brains is responsible for the progressive proximation of the two brain tissues. Bars, 500 μm.
(A) Definition of tissue axes used in the analysis (a) and secondary brain- dependent bending of the primary head axis relative to that of the trunk (b). (a) H, host (primary) brain axis; G, graft-dependent (secondary) brain axis; B, the axis of boundary head ectoderm partitioning the primary and secondary heads; T, the axis of the anterior trunk, with which the H axis made the angle θ. (b) Distribution of the H axis bending angle θ measured in 13 embryos of each group at st. 8. Without graft, the bending was on either side. **, $P < 0.001$ using the Mann– Whitney U test.
(B) and (C) Analyses of trajectories of SN-EGFP-labeled cells around axes H, B, and G, during the brain histogenesis stages. (i) to (xii) The areas where short tracks of SN-EGFP-labeled cells provide information concerning cell dynamics in neural tube formation and head ectoderm precursors covering or intervening the two neural tubes.
(B) An embryo with the node graft in Figure 5(B). (a)-(d) Bright-field images at stages 6+, 7, 8, and 9. The original positions of the primary and secondary brain axes at st. 6+, which were 1,120 μm apart, are indicated using broken and dotted lines, respectively. These axes became closer by 450 μm at st. 8. (a’)-(d’) and (a’’)-(d’’) Short tracks of SN-EGFP-labeled cells over a duration of 75 min drawn relative to one of the three axes, H, B, or G. The most recent cell positions are indicated in white; single white dots represent cells that remained in their position.
(i) The primary neural plate at st. 6+, where the cells showed anteromedial cell convergence around the H axis (44 ± 15 μm/h). (ii) The juxtaposed cell sheet pulled toward the H axis (47 ± 12 μm/h). (iii) The cell movements relative to the B axis in the corresponding area were negligible. (iv) As the neural tube developed, the cell convergence toward the H axis became more extensive (50 ± 15 μm/h). (v) Around the G axis, the axial convergence was moderate (26 ± 5 μm/h), reflecting the smaller scale of secondary neural tube histogenesis. (vi) Cell movements in and around the neural tubes diminished at st. 8, except for their posterior ends (41 ± 13 μm/h), which pulled each other. (vii) The head ectoderm penetrating through the B axis (29 ± 7 μm/h) partitioned the primary and secondary heads. The two heads in this embryo pulled each other further at st. 9 (viii, 52 ± 13 μm/h; ix, 51 ± 9 μm/h) along a thick neural tissue bridge [arrowhead in (d)], creating a large θ (~40 degrees), which was observed in 3 out of the 13 node-grafted embryos.
(C) An embryo with the AME graft in Figure 5(D). (a)(b) Bright-field images at st. 7 and 8, when the unstructured secondary neural plate was reorganized into the symmetrical brain tissue (Movie 8). The tissue reorganization aligned the secondary brain with the primary brain, which was observed in 9/13 cases of AME graft-elicited secondary brain development. (a’)(b’) Trajectories of SN-EGFP-labeled cells over 60 min around the H and B axes. At st. 7, H axis-directed cell movements in the primary brain (x, 58 ± 15 μm/h) and H axis-directed cell movements in the forming secondary brain (xi, 59 ± 19 μm/h) were both extensive.
(xii) The tissue reorganization in the secondary brain continued to st. 8 (46 ± 13 μm/h).

© 2022. Published by The Company of Biologists under the terms of the Creative Commons Attribution License (https://creativecommons.org/licenses/by/4.0/).

Figure S11

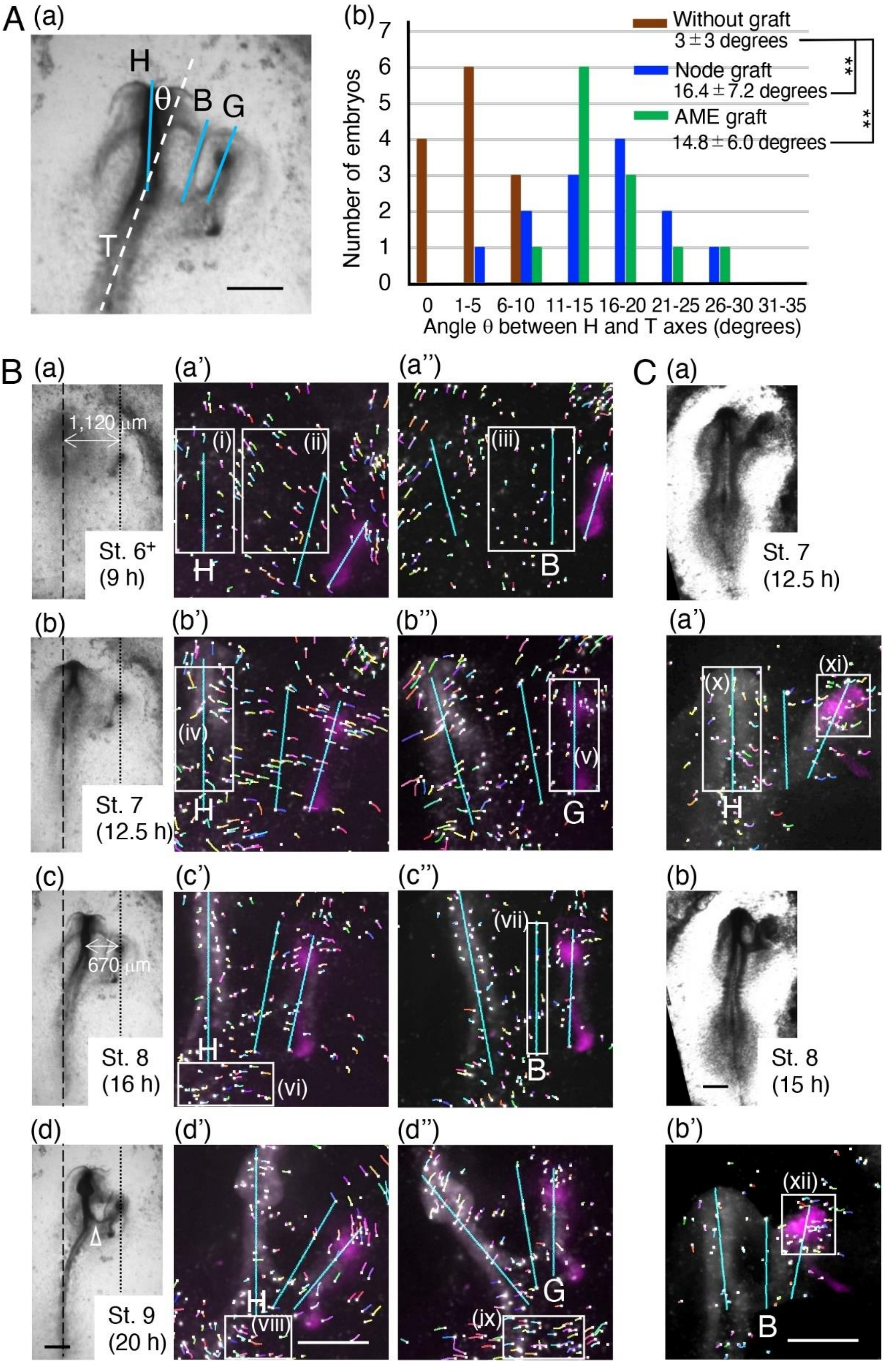

© 2022. Published by The Company of Biologists under the terms of the Creative Commons Attribution License (https://creativecommons.org/licenses/by/4.0/).

Fourth decision letter

MS ID#: DEVELOP/2021/199999

MS TITLE: Live imaging of avian epiblast and anterior mesendoderm grafting reveals the complexity of cell dynamics during early brain development

AUTHORS: Koya Yoshihi, Kagayaki Kato, Hideaki Iida, Machiko Teramoto, Akihito Kawamura, Yusaku Watanabe, Mitsuo Nunome, Mikiharu Nakano, Yoichi Matsuda, Yuki Sato, Hidenobu Mizuno, Takuji Iwasato, Yasuo Ishii, and Hisato Kondoh
ARTICLE TYPE: Research Article

I am happy to tell you that your manuscript has been accepted for publication in Development, pending our standard ethics checks.

© 2022. Published by The Company of Biologists under the terms of the Creative Commons Attribution License (https://creativecommons.org/licenses/by/4.0/).
